# Nuts and seeds consumption and risk of cardiovascular disease, type 2 diabetes and their risk factors: a systematic review and meta-analysis

**DOI:** 10.29219/fnr.v67.8961

**Published:** 2023-02-14

**Authors:** Erik Kristoffer Arnesen, Birna Thorisdottir, Linnea Bärebring, Fredrik Söderlund, Bright I. Nwaru, Ulrike Spielau, Jutta Dierkes, Alfons Ramel, Christel Lamberg-Allardt, Agneta Åkesson

**Affiliations:** 1 Department of Nutrition, Institute of Basic Medical Sciences, University of Oslo, Oslo, Norway; 2 Health Science Institute, University of Iceland, Reykjavik, Iceland; 3 Department of Internal Medicine and Clinical Nutrition, Institute of Medicine, Sahlgrenska Academy, University of Gothenburg, Gothenburg, Sweden; 4 Unit of Cardiovascular and Nutritional Epidemiology, Institute of Environmental Medicine, Karolinska Institutet, Stockholm, Sweden; 5 Krefting Research Centre, Institute of Medicine, University of Gothenburg, Gothenburg, Sweden; 6 Centre for Nutrition, Department of Clinical Medicine, University of Bergen, Bergen, Norway; 7 Mohn Nutrition Research Laboratory, Department of Clinical Science, University of Bergen, Bergen, Norway; 8 Department of Medical Biochemistry and Pharmacology, Haukeland University Hospital, Bergen, Norway; 9 Faculty of Food Science and Nutrition, University of Iceland, Reykjavik, Iceland; 10 Department of Food and Nutrition, University of Helsinki, Helsinki, Finland

**Keywords:** nuts, cardiovascular disease, atherosclerosis, diabetes mellitus type 2, systematic review, meta-analysis

## Abstract

**Objectives:**

We aimed to systematically review studies and evaluate the strength of the evidence on nuts/seeds consumption and cardiometabolic diseases and their risk factors among adults.

**Methods:**

A protocol was pre-registered in PROSPERO (CRD42021270554). We searched MEDLINE, Embase, Cochrane Central Register of Controlled Trials and Scopus up to September 20, 2021 for prospective cohort studies and ≥12-week randomized controlled trials (RCTs). Main outcomes were cardiovascular disease (CVD), coronary heart disease (CHD), stroke and type 2 diabetes (T2D), secondary total-/low density lipoprotein (LDL)-cholesterol, blood pressure and glycaemic markers. Data extraction and risk of bias (RoB) assessments (using RoB 2.0 and RoB-NObS) were performed in duplicate. Effect sizes were pooled using random-effects meta-analyses and expressed as relative risk (RR) or weighted mean differences with 95% confidence intervals (CI); heterogeneity quantified as *I*^2^. One-stage dose-response analyses assessed the linear and non-linear associations with CVD, CHD, stroke and T2D. The strength of evidence was classified per the World Cancer Research Fund criteria.

**Results:**

After screening 23,244 references, we included 42 papers from cohort studies (28 unique cohorts, 1,890,573 participants) and 18 RCTs (2,266 participants). In the cohorts, mainly populations with low consumption, high versus low total nuts/seeds consumption was inversely associated with total CVD (RR 0.81; 95% CI 0.75, 0.86; *I*^2^ = 67%), CVD mortality (0.77; 0.72, 0.82; *I*^2^ = 59.3%), CHD (0.82; 0.76, 0.89; *I*^2^ = 64%), CHD mortality (0.75; 0.65, 0.87; *I*^2^ = 66.9%) and non-fatal CHD (0.85; 0.75, 0.96; *I*^2^ = 62.2%). According to the non-linear dose-response analyses, consumption of 30 g/day of total nuts/seeds was associated with RRs of similar magnitude. For stroke and T2D the summary RR for high versus low intake was 0.91 (95% CI 0.85, 0.97; *I*^2^ = 24.8%) and 0.95 (0.75, 1.21; *I*^2^ = 82.2%). Intake of nuts (median ~50 g/day) lowered total (−0.15 mmol/L; −0.22, −0.08; *I*^2^ = 31.2%) and LDL-cholesterol (−0.13 mmol/L; −0.21, −0.05; *I*^2^ = 68.6%), but not blood pressure. Findings on fasting glucose, HbA1c and insulin resistance were conflicting. The results were robust to sensitivity and subgroup analyses. We rated the associations between nuts/seeds and both CVD and CHD as *probable*. There was limited but *suggestive* evidence for no association with stroke. No conclusion could be made for T2D.

**Conclusion:**

There is a probable relationship between consumption of nuts/seeds and lower risk of CVD, mostly driven by CHD, possibly in part through effects on blood lipids. More research on stroke and T2D may affect the conclusions. The evidence of specific nuts should be further investigated.

## Popular scientific summary

- Frequent consumption of nuts has previously been associated with lower risk of cardiovascular disease (CVD) and coronary heart disease (CHD).
- Consumption of total nuts/seeds is associated with lower risk of CVD and CHD in a dose-dependent manner.
- Smaller or unclear associations were found for risk of stroke and type 2 diabetes.
- Nuts modestly lowered blood lipids, but had no effect on blood pressure in randomized controlled trials.
- The favourable associations with CVD and CHD are probably causal.

The inclusion of nuts in official food-based dietary guidelines is relatively recent, despite a Food and Agricultural Organization/World Health Organization statement from 2003 on a probable association between unsalted nuts and reduced risk of cardiovascular disease (CVD) (1). The 2012 Nordic Nutrition Recommendations (NNR) explicitly recommended an increased consumption of nuts and seeds, while this recommendation was true only for 19% of all national food-based dietary guidelines reviewed from 1986 to 2017 (2). While some dietary guidelines include them simply as a source of protein or unsaturated fatty acids, nuts and seeds are good sources of many biologically active components, such as polyunsaturated fatty acids (PUFA), micronutrients (e.g. vitamin E, minerals), dietary fibre, polyphenols, flavonoids and phytosterols, that have various, potentially beneficial properties for cardiometabolic risk factors. Consequently, nuts and seeds are important parts of healthy dietary patterns and eating plans such as the healthy Nordic diet, the Dietary Approaches to Stop Hypertension (DASH), Mediterranean-style and plant-based/vegetarian dietary patterns (3, 4), and in clinical CVD prevention guidelines (5, 6). Moreover, the Global Burden of Disease study ranked low consumption of nuts and seeds as a major dietary contributor to deaths and the overall disease burden on the global scale (7, 8). Still, the mean intakes at population-level are marginal and far from current recommendations, especially in Europe (8, 9).

Interest in nuts for prevention of cardiometabolic disease emerged in 1992 after Fraser et al. reported a lower risk of fatal coronary heart disease (CHD) and myocardial infarction (MI) among frequent nut consumers (>4 servings per week) in the Adventist Health Study (10). This was followed by intervention studies that found significant reductions in total and LDL-cholesterol from walnuts or almonds (11, 12) and other large prospective cohort studies from the USA (13, 14). The US Food and Drug Association approved a qualified health claim regarding nuts (42 g/day) for reduced risk of heart disease in 2003, while a health claim related to walnuts and improved endothelium-dependent vasodilation is approved in the European Union (15).

During the past decade, a substantial number of systematic reviews (SR) and meta-analyses have been published on nuts and various endpoints, including findings from observational and intervention studies (16–23). Among the most recent, Becerra-Tomas et al. performed a SR with meta-analyses commissioned by the European Association for the Study of Diabetes (EASD) on nut consumption (excluding seeds) and the risk of CVD incidence or mortality (17). They included 19 prospective studies, published between 1992 and 2018. Compared to the lowest category, the highest consumption category of nuts was associated with lower incidence of CVD, CHD and atrial fibrillation, and with lower mortality from CVD, CHD and stroke (relative risk (RR) reductions ranging from −23 to −15%), but not with stroke incidence nor heart failure. In 2021, the same authors reported no significant association between nut consumption and type 2 diabetes (T2D) in an SR and meta-analysis of prospective and cross-sectional studies (18).

Since those SRs were conducted, several new, large-scale cohort studies have been published. Furthermore, despite a relatively large amount of trials and prospective studies, SRs on nuts have so far graded the certainty in the evidence as ‘low’ or ‘very low’ for several outcomes (17–19, 24, 25), implying that further research may change the confidence and effect estimates (26). Thus, consumption of nuts and seeds in relation to CVD and T2D was considered a prioritized subject for a *de novo* systematic review by the NNR 2022 Committee (27, 28). An initial scoping review by the Committee in 2020 identified new data since 2011 that were considered to have the potential to change the NNR food-based dietary guidance (FBDG) related to nuts and seeds in relation to CVD, T2D and risk factors.

The aim of this systematic review was to examine the evidence for an association between consumption of nuts and seeds and the incidence of or mortality from CVD and T2D, and the effects of nuts and seeds on intermediate cardiometabolic risk factors. For this SR, we included both nuts and seeds as they are grouped in several dietary guidelines and have similar nutritional characteristics (29).

## Methods

This systematic review followed the guidelines developed for the NNR 2022 (30, 31) and the Preferred Reporting Items for SR and Meta-Analyses (32, 33). A protocol was pre-registered online on PROSPERO (https://www.crd.york.ac.uk/prospero) with review ID CRD42021270554.

A focused research question was developed by the NNR 2022 Committee, defining the population/participants, intervention/exposure, control, outcome, timeframe, study design and settings (PI/ECOTSS), in an iterative process with the review authors. The funding source for NNR 2022 was the Nordic Council of Ministers and governmental food and health authorities of Norway, Finland, Sweden, Denmark, and Iceland (27).

### Eligibility criteria

The inclusion and exclusion criteria are outlined in the PI/ECOTSS statement (Table 1). We included original research articles with a prospective cohort design (i.e. cohort, case-cohort or nested case-control studies) and randomized controlled trials (RCTs) involving generally non-pregnant healthy adults (>18 years of age) from the general population (including people with elevated serum lipids, blood pressure, obesity, metabolic syndrome, impaired glucose tolerance or insulin resistance). Studies on secondary prevention, that is, established CVD or T2D as well as weight loss trials, were excluded. There were no restrictions concerning publication language, sample size or risk of bias (RoB).

**Table 1:** Eligibility criteria for population/participants, intervention/exposure, control, outcome, timeframe, study design and settings (PI/ECOTSS)

Population	Intervention or Exposure	Comparators	Outcomes	Timing	Setting	Study design
Adults, general population	Nuts and seeds intake, including peanuts	Dose-response (per serving/day) or high versus low or no intake	Fatal or non-fatal atherosclerotic cardiovascular disease (including coronary artery disease, myocardial infarction, ischemic stroke, CVD mortality), type 2 diabetes incidence and mortality. RCTs only: Changes in atherogenic serum lipids, blood pressure, fasting glycaemia (glucose, HbA1c), insulin and insulin resistance/insulin sensitivity.	Minimum 12 months follow-up in cohort studies. Minimum 12-week intervention in intervention studies.	Relevant for the general population in the Nordic and Baltic countries. Not weight-loss studies.	RCTs (for risk factors), prospective cohort studies

The exposure of interest was consumption of total or individual types of edible nuts and seeds based on culinary practice rather than a strict botanical definition (e.g. almonds, flaxseeds, peanuts, sunflower seeds, walnuts etc. were eligible), but not betel nuts, coconuts, cola nuts or ‘soy nuts’/roasted soybeans. As the focus was on nuts/seeds consumed as a food, nut or seed oils or extracts were excluded, as were nuts/seeds grinded and consumed as a ‘supplement’, added to beverages, bread etc. However, nut spreads (‘butter’) was included as most studies included them in the definition of total nuts. Studies based on dietary patterns containing nuts (e.g. Mediterranean diets), multifactorial interventions, or studies combining nuts/seeds with for example, fruits or legumes, were excluded if they did not provide specific quantitative analyses of nuts and outcomes. We did include studies reporting substitution analyses of nuts and seeds replacing other food sources, such as red meat.

The following primary and secondary outcomes were considered: 1) incidence and mortality of atherosclerotic CVD (including coronary artery disease (coronary/ischaemic heart disease), MI, total and ischemic stroke, total CVD as a composite outcome) and T2D; 2) changes in atherogenic serum lipids [primarily total cholesterol (TC) and LDL-cholesterol (LDL-C)], blood pressure (systolic and diastolic), fasting glycaemia (glucose, glycated haemoglobin A1c (HbA1c)), insulin and insulin resistance/insulin sensitivity.

For the cardiometabolic risk factors, only randomized controlled parallel or crossover trials with a minimum 12-week intervention period were included. This cut-off was chosen because our interest was in ‘chronic’ effects relevant to primary/primordial prevention rather than purely mechanistic or therapeutic effects. For the same reasoning, we also excluded trials aiming for weight-loss and calorie restriction.

When more than one publication on an outcome was available for the same study/cohort, we included the one with the most participants/cases, the longest follow-up period or the one with most detailed data relevant to our research question.

### Information sources and search strategy

A research librarian at the medical library at the University of Oslo, Oslo, Norway, performed a comprehensive literature search of MEDLINE (Ovid), Embase (Ovid), Cochrane Central Register of Controlled Trials, and Scopus, for publications up to September 20, 2021.

The search strategy (Supplementary file) was developed in collaboration with the authors, led by EKA, BT and AÅ, and was peer-reviewed by research librarians at Karolinska Institute, Stockholm, Sweden. There were no date or language limitations in the search strategy. ‘Grey literature’ or conference abstract searches or were not performed, as they would not have allowed for thorough RoB assessments (30).

### Selection and data collection process

Four of the SR authors (EKA, BT, CLA, FS) screened and selected studies for inclusion/exclusion, working independently. Screening of titles and abstracts was performed with the web-based *Rayyan* (https://rayyan.qcri.org) before full-text article screening. Reference lists from included articles and previous SRs were also scrutinized for potentially eligible studies. Disagreements about inclusion/exclusions were resolved until consensus together with a senior team member.

Data from full-text papers were extracted in standardized extraction forms by three reviewers (EKA, FS and BN) working independently, and harmonized by EKA. We extracted information regarding study design, participant characteristics and settings, interventions/exposures (i.e. type of nuts), endpoints, number of cases per endpoint, analytic approaches and results (unadjusted and adjusted estimates). Nutrition-specific elements, such as intake levels (‘dose’) and dietary assessment methods, were also extracted. Correspondence by e-mail with the primary research authors was attempted to retrieve data considered necessary for meta-analyses. We received additional data from four studies (34–37).

### Study risk of bias assessment

Risk of bias was appraised in duplicate by several reviewers working independently before a final harmonization.

We used the Cochrane Risk of bias 2.0 tool for RCTs, which assesses selection bias (arising from the randomization process), performance bias (deviation from the intended interventions), detection bias, attrition bias, and selective reporting bias (38). For crossover RCTs, also period and carryover effects were considered. Each domain and the summary RoB were judged as either low, ‘some concern’ or high RoB, according to the RoB 2.0 algorithms.

For observational studies, the assessment was based on the ‘Risk of Bias for Nutrition Observational Studies’ (RoB-NObS) tool developed by the Nutrition Evidence Systematic Review (NESR). RoB-NObS in turn builds on the ROBINS-I and the causal inference framework (consistency, positivity, exchangeability), based on a ‘target trial’, meaning that the studies are assessed against a hypothetical high-quality, randomized trial with little confounding and other sources of bias (39, 40). The domains appraised with RoB-NObS are confounding, selection of participants, classification of interventions/exposures, deviations from intended interventions/exposures, missing data, measurement of outcomes, and selection of the reported result. An overall RoB was judged as low, moderate, serious or critical.

### Synthesis methods

In accordance with the protocol, studies were pooled in meta-analyses if there were at least five studies reporting the same exposure and type of outcome, to be able to reliably assess between-study heterogeneity (41). Quantitative syntheses were performed for overall CVD/CVD mortality; CHD/CHD mortality; total stroke/stroke mortality; ischaemic stroke; T2D, and among the cardiometabolic risk factors total cholesterol (total-C), LDL-cholesterol (LDL-C) and blood pressure. Subgroup and sensitivity analyses were performed if at least 10 studies were included in the meta-analysis. If a cohort study reported results for separate subgroups (e.g. by sex) with similar exposures and outcomes, the results were first meta-analysed with a fixed-effect model for the main analyses (42–44). Estimates for non-fatal and fatal events within studies were pooled for the meta-analyses on total CVD, CHD and stroke events. Separate results for ischaemic and haemorrhagic stroke were also pooled for the meta-analysis on total stroke.

The cohort studies were meta-analysed by a random-effects model, accounting for both within- and between-study variances estimated with the restricted maximum-likelihood (REML) method. A random-effects analysis does not assume one true effect but estimates the mean of a distribution of effects. In one instance (45), odds ratios were converted to risk ratios (46), otherwise hazard ratios (HR) and relative risk (RR) were considered equivalent (47) and expressed as RRs. HR/RRs and their 95% confidence intervals (CI) or standard errors were log-transformed and summarized to assess the highest versus lowest consumption categories and dose-response relationships. Studies only reporting linear effect estimates (e.g. per serving/day) were excluded from the ‘high versus low’ meta-analyses but were included in the dose-response analyses. All analyses were performed with Stata/SE version 17.0 (StataCorp LLC, College Station, Texas, USA).

Linear dose-response analyses were performed based on the method by Greenland and Longnecker (48) and Orsini et al. (49) to estimate associations up to 30 g/day [approximately one ‘handful’ (50)] intake of total nuts (or 10 g/day intake of specific nuts), with the covariances estimated by the Greenland and Longnecker method (48, 49), which were then pooled in the random-effects meta-analyses as described above.

In addition to the log RRs and 95% CI per intake category, the dose-response analyses required the doses per category and the distribution of person-years and cases within each study. We used the mean or median grams of nuts per category, if reported. If the nut intake in each category was expressed as a range, we defined the intake as the midpoint of the range. If the upper and/or lower intake category was open-ended, we assumed the intake range had the same width as that of the adjacent category. When nut consumption was expressed as servings or frequency, we assumed that one serving equalled 28 g (1 oz), if not otherwise specified. When the doses were reported as % of total energy intake (E%), the corresponding g/day were estimate with energy values according to the Norwegian food composition table (e.g. walnuts = 6.8 kcal/g) (51). We considered the lowest consumption category as the reference in each study; if a study used a different category as reference, the effect estimates and 95% CIs were recalculated as per Hamling et al. (52). When studies had already reported a linear dose-response trend, with CI or standard error, this was used directly. Missing numbers of cases or person-years per category was estimated according to Greenland (53) or Aune (54).

Nonlinear dose-response trend analyses were conducted with a one-stage mixed-effects approach (55, 56) using the *drmeta* program in Stata, modelled with restricted cubic splines with three knots fixed at the 10th, 50th and 90th percentiles of exposure (57). Departure from linearity was examined by Wald-type χ^2^ tests against the null-hypothesis that the coefficient of the second spline equalled 0.

Effects of nuts and seeds interventions on total-C, LDL-C and systolic blood pressure (SBP) were also examined in random-effects REML meta-analyses to estimate weighted mean differences and 95% CI between nut consumption and control. Mean differences and their standard deviations (SD) between the intervention and control group at follow-up were the primary effects of interest. If differences at follow-up were not reported, change differences were used as measures of net differences, preferably differences in change from ANCOVA analyses or mixed models adjusting for baseline, if reported (58–62). SDs in change were calculated from other measures of variance if not directly reported (60). For cross-over trials, we used results from paired analyses accounting for intra-individual correlation as reported, or calculated SDs with a correlation coefficient of 0.6, which is a conservative estimate (63, 64). Total-C and LDL-C reported in mg/dL was converted into mmol/L by dividing mg/ dL with 38.67.

For intervention studies with more than one intervention arm (e.g. with different doses), only one comparator was included in the meta-analyses. If different doses were used, we chose the intervention dose closest to 30 g, that is, the recommended intake. If both office and ambulatory blood pressure was reported, we included only office blood pressure, as this was the most used method.

For all meta-analyses, we assessed homogeneity between studies using the Cochran Q test (with *P* > 0.1 as a significance threshold), and used the *I*^2^ statistic (range 0–100) to quantify inconsistency, that is, the total variability explained by between-study heterogeneity. An *I*^2^ of ≥50% was considered to indicate ‘substantial’ and ≥75% ‘considerable’ heterogeneity (59). We also visually assessed Galbraith plots and excluded one study at the time to identify outliers and explore potential influences on the overall effect estimate. Heterogeneity was further explored post hoc in subgroup analyses and random-effects meta-regression analyses if there were at least 10 studies per exposure-outcome pair. Both clinical and methodological sources of heterogeneity were examined, that is, type of nuts, geographic region (Europe, USA, Asia, Australia or multinational), overall study RoB, study duration/follow-up time, and mean age at baseline. For the cohort studies, we also performed subgroup analyses according to adjustment for blood lipids or blood pressure/hypertension, which may be mediators of the associations. We also considered patient characteristics (e.g. metabolic syndrome) and baseline level of total-C, LDL-C or SBP in the RCTs as sources of heterogeneity.

To assess small study effects, visual inspection of funnel plots and Egger’s regression tests (significance level *P* > 0.1) were evaluated if there were at least 10 effect estimates (65). If applicable, the Duval and Tweedie trim-and-fill method was used to impute potentially missing studies due to publication bias.

### Certainty assessment

An overall strength of evidence was judged per endpoint mainly based on RoB, inconsistency/heterogeneity and precision of the evidence (see 30, 31). This was only done for the primary outcomes, that is, CVD/CHD/IHD and T2D. We categorized the strength of evidence in line with the World Cancer Research Fund’s grading: ‘Convincing’, ‘Probable’, ‘Limited – suggestive’, ‘Limited – no conclusion’, ‘Substantial effects unlikely’. A *convincing* body of evidence implied that it was strong enough to support a causal relationship or lack of a relationship (30), and required that several conditions were met, such as evidence from more than one study type. The evidence for a causal relationship was considered as *probable* when there was evidence from at least two independent cohort studies, no unexplained heterogeneity between or within study types, high-quality studies (low RoB) to confidentially exclude possible random or systematic errors, and evidence for biological plausibility. If there was evidence for an association or effect from at least two independent cohort studies, a consistent direction of the effect and evidence for biological plausibility, the evidence was considered *limited – suggestive.* The evidence was considered *limited – no conclusion* if it was so limited that no firm conclusion could be made. On the other hand, if there was strong enough evidence to support that there is a convincing absence of a causal relationship, we considered that any substantial effects were *unlikely*.

## Results

**Fig. 1:**
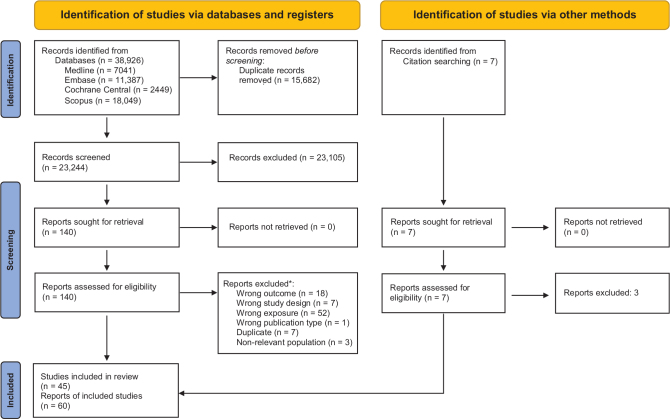
Study selection flowchart. Source: Page et al. (32). *Some had more than 1 reason for exclusion.

The systematic literature search identified a total of 23,244 references after duplicates were removed, out of which 140 were further assessed after the initial screening of titles and abstracts (Table 2 and Fig. 1). From reference lists of the papers assessed in full-text, another 7 were found eligible for full-text assessment (66–72), resulting in 147 references assessed in full-text. A list of papers excluded after full-text assessment, and reasons for exclusion, is provided in Supplementary Table 1. Finally, 60 papers were extracted and included in qualitative assessments.

**Table 2:** Documentation of literature search

Database	Number of retrieved references
MEDLINE (Ovid)	7,041
Embase (Ovid)	11,387
Cochrane Central Register of Controlled Trials (Wiley)	2,449
Scopus (Elsevier)	18,049
Number of references before deduplication:	38,926
Number of references after deduplication:	23,244

### Study characteristics

The 60 included papers represented 46 unique studies, of which 40 (27 individual cohorts) were prospective cohort studies (10, 35–37, 42–45, 68, 70–100), two (one cohort) were case-cohort studies (101, 102) and 18 were RCTs (34, 103–119). Characteristics of the cohort studies are described briefly in Table 3 and Supplementary Table 1, and the RCTs are described in Table 4.

**Table 3:** Selected characteristics of the included cohort studies^1^

Name of cohort (reference)	Author (year)	Country Sex Age	Sample size	Exposures Mean/median intake (if reported)	Outcomes measured	Follow-up time (years)	Overall RoB
Adventist Health Study (10)	Fraser (1992)	USA 62.6% female Mean 52.5 years	26,473	Nuts	CHD	6	Serious
ARIC (35, 85)	Haring (2014)	USA 55.8% female 45–64 years	12,066	Nuts and peanut butter Median 0.2 servings/day	CHD	22	Moderate
	Haring (2015)	USA 55.9% female 45–64 years	11,601	Nuts and peanut butter Median 0.21 servings/day	Stroke	22.7	Moderate
Blue Mountains Eye study (80)	Gopinath (2015)	Australia 55.9% female Mean 65.4 years	2,893	Nuts	CVD mortality IHD mortality Stroke mortality	15	Serious
EPIC (36, 95, 101, 102)	Buijsse et al. (2015)	8 European countries 62.7% female Mean 52.2 years	14,939	Nuts and seeds Median 0.6 g/day	T2D	12.3	Serious
	Ibsen (2020)	8 European countries 62% female Median 53	15,450	Nuts versus red/processed meat	T2D	12.3	Serious
	Perez-Cornago (2021)	10 European countries 71.4% female Mean 51.2 years	490,311	Nuts and seeds Median 0.775 g/day	IHD	12.6	Serious
	Tong (2020)	Multinational (Europe) 66.5% female Mean 51.2 years	418,329	Nuts and seeds Median 0.8 g/day	Stroke	12.7	Serious
EPIC-Potsdam (71, 82)	von Ruesten (2013)	Germany 61.3% female 35–65 years	23,531	Nuts Median 0.8 g/day	CVD T2D	8	Serious
	di Giuseppe (2015)	Germany 59% female Mean 51 years	26,285	Nuts Median 0.82 g/day	Stroke	8.3	Serious
Golestan Cohort Study (81)	Eslamparast (2017)	Iran 57.5% female Mean 52 years	49,112	Nuts Mean 3.5 g/day in men, 2.6 g/day in women	CVD mortality	7	Serious
HPFS (73)	Al-Shaar (2020)	USA 100% male 40–75 years	43,272	Nuts versus red/processed meat	CHD	≤30	Serious
Iowa Women’s Health Study (68, 78)	Blomhoff (2006)	USA 100% female 55–69 years	31,788	Nuts and peanut butter Mean 2.37 servings/week	CVD mortality CHD mortality	15	Serious
	Parker (2003)		35,988	Nuts and peanut butter	T2D	12	Serious
Isfahan Cohort Study (93)	Mohammadifard (2020)	Iran 51.2% female Mean 50.7 years	5,432	Nuts	CVD mortality	13	Moderate
Japan Public Health Center-based Prospective Study (87)	Ikehara (2021)	Japan 54% female 45–74 years	74,793	Peanuts	CVD IHD Stroke	14.8	Serious
Linxian Nutrition Intervention Trial (99)	Wang (2016)	China 54.8% female 40–69 years	2,445	Nuts Median 0.3 servings/month	CHD mortality Stroke mortality	26	Serious
Million Veterans Program (89)	Ivey (2021)	USA 10% female Mean 64 years	149,827	Nuts and peanut butter	CVD mortality CAD Stroke	3.5	Serious
Moli-Sani study (79)	Bonaccio (2015)	Italy 53% female Mean 54.7 years	19,386	Nuts	CVD mortality	4.3	Serious
Netherlands Cohort Study (97, 98)	van den Brandt (2015)	The Netherlands 52.8% female 55–69 years	3,693	Nuts and peanut butter Mean 8.1 g nuts/1.4 g peanut butter in men, 4.4 g nuts/1.2 g peanut butter in women	CVD mortality IHD mortality Stroke mortality	10	Moderate
	van den Brandt (2019)		3,202	Nuts versus meat	CVD mortality	10	Moderate
NHS (76, 94)	Bernstein (2010)	USA 100% female 30–55 years	84,136	Nuts	CHD	≤26	Moderate
	Pan (2013)	USA 100% female 35–77 years	137,956	Nuts Mean 3.36 g/day	T2D	10	Serious
NHS & HPFS (42, 77, 92, 100)	Bernstein (2012)	USA 66% female 30–75 years	127,160	Nuts	Stroke	≤26	Serious
	Guasch-Ferré (2017)	USA 80% female 25–75 years	210,836	Nuts and peanut butter	CVD CHD Stroke	≤32	Serious
	Liu (2021)	USA 71.8% female 30–75 years	93,340	Walnuts	CVD mortality	≤20	Moderate
	Wurtz (2021)	USA 81.4% female 25–75 years	148,853	Nuts and peanut butter versus red/processed meat	T2D	4-year periods	Serious
NIH-AARP study (75)	Amba (2019)	USA 43.6% female Median 61.9 years	374,101	Nuts Median 2.9 g/day	CVD mortality T2D mortality	15.5	Serious
Physicians’ Health Study (74, 83, 86, 90)	Albert (2002)	USA 100% male 40–84 years	21,454	Nuts	CHD	17	Serious
	Djoussé (2010)		21,078	Nuts and peanut butter	Stroke	21.1	Serious
	Kochar (2010)		20,224	Nuts	T2D	19.2	Serious
	Hshieh (2015)		20,742	Nuts and peanut butter Median 1 serving/week	CVD mortality CHD mortality Stroke mortality	9.6	Serious
PREDIMED (84)	Guasch-Ferré (2013)	Spain 58% female Mean 67 years	7,216	Nuts	CVD mortality	4.8	Moderate
PURE (96)	De Souza (2020)	16 countries 57.6% female 35–70 years	85,713	Nuts Mean 6.4 g/day	CVD MI Stroke	9.5	Moderate
SCCS/SMHS/SWHS (43)	Luu (2015)	USA & China 56.6% female 40–79 years	206,029	Nuts and peanut butter, only peanuts in SMHS/SWHS SCCS: Mean 12.25 g/day, SMHS/SWHS: 2 g/day	CVD mortality IHD mortality Stroke mortality T2D mortality	5.4–12.2	Moderate
Cohort of Swedish Men/Swedish Mammography Cohort (91)	Larsson (2018)	Sweden 46.4% female Mean 57.7 years	61,364	Nuts	MI Ischaemic stroke	17	Moderate
SWHS (70)	Villegas (2008)	China 100% female 40–70 years	64,191	Peanuts Mean 1.5 g/day	T2D	4.6	Moderate
Takayama Study (44)	Yamakawa (2021)	Japan 54% female ≥35 years	29,079	Nuts Mean 1.8 g/day in men, 1.5 g/day in women	CVD mortality	13.7 in men, 14.4 in women	Moderate
Tehran Lipid and Glucose Study (45)	Asghari (2017)	Iran 53.6% female Mean 40.1 years	1,984	Nuts and seeds Median 1.19 servings/week	T2D	6.2	Serious
Women’s Health Initiative (37, 72)	Sun (2021)	USA 100% female 50–79 years	102,521	Nuts and seeds Median 0.2 oz-equivalents/day	CVD mortality	18	Moderate
	Yaemsiri (2012)		87,025	Nuts	Ischaemic stroke	7.6	Moderate
Women’s Health Study (88)	Imran (2021)	USA 100% female Mean 54.6 years	39,167	Nuts	CVD mortality	19	Moderate

1 Abbreviations: CHD, coronary heart disease; CVD, cardiovascular disease; HPFS, Health-Professionals’ Follow-up Study; IHD, ischaemic heart disease; MI, myocardial infarction; NHS, Nurses’ Health Study; RoB, risk of bias; SMHS/SWHS, Shanghai Men’s/Women’s Health Study; T2D, type 2 diabetes; PURE, Prospective Urban and Rural Epidemiology study.

**Table 4:** Characteristics of randomized controlled trials^1^

Author (year) (reference)	Design	Country	Population	Outcome(s)	Intervention	Intervention dose	Control	Sample size (analysed)	Duration	Overall RoB	Funding
Al Abdrabalnabi (2020) (103)	Parallel	USA & Spain	WAHA study; age 62–79 years; normotensive or mild hypertension, low CVD risk	Blood pressure, HDL-C, TG, glucose	Walnuts	15 E% (i.e., 30, 45 or 60 g/day)	No walnuts.	I: 319 C: 306	2 years	Some concerns	Industry
Barbour (2015) (104)	Crossover	Australia	Healthy, overweight; mean age 65 years	Lipids, glucose, insulin sensitivity	Peanuts	15–20 E% (i.e. 84 g/day for men, 56 g/day for women)	No nuts	61	12 weeks	Some concerns	Mixed
Bashan & Bakman (2018) (105)	Parallel	Turkey	Adults with dyslipidaemia; mean age 41 years	Lipids	Walnuts + AHA dietary guidelines	‘One handful’ (40–50 g)	AHA dietary guidelines	I: 73 C: 72	12 weeks	High	Agency
Casas-Agustench (2011) (106)	Parallel	Spain	Adults with metabolic syndrome, mean age 52 years	Lipids, glucose, insulin resistance	Walnuts, hazelnuts and almonds + prudent diet	30 g/day	No nuts/peanuts + prudent diet	I: 25 C: 25	12 weeks	Some concerns	Mixed
Coates (2020) (107)	Parallel	Australia	Adults with overweight/obesity; 50–80 years	Blood pressure, lipids, glucose insulin resistance	Almonds	15 E% 6 days/week	Carbohydrate-rich snack foods; no nuts	I: 63 C: 64	12 weeks	Low	Industry
Gulati (2014) (34)	Parallel	India	Adults with metabolic syndrome; mean age 42.5 years	Lipids, glucose, HbA1c	Pistachio nuts	20 E%	Dietary guidelines	I: 30 C: 30	24 weeks	Some concerns	Industry
Hernandez-Alonso (2014) (108)	Crossover	Spain	Adults with prediabetes; age 25–65 years	Blood pressure, lipids, glucose, insulin resistance	Pistachio nuts + isocaloric individual diet	2 oz (57 g)/day	Other fatty foods, mostly olive oil + isocaloric individualized diet	54	16 weeks	Some concerns	Industry
Hunter (2021) (109)	Parallel	USA	Healthy adults; BMI ≥27 kg/m^2^; mean age 37 years	Lipids, glucose, HbA1c, insulin resistance	Almonds for breakfast and snack + avoid other nuts	1.5 oz (42 g)/day	Habitual breakfast and snack	I: 69 C: 65	6 months	Low	Industry
Hwang (2019) (110)	Crossover	Korea	Adults with metabolic syndrome; mean age 39.4 years	Blood pressure, lipids, glucose, HbA1c	Walnuts	45 g/day	White bread	84	16 weeks	High	Industry
Kasliwal (2015) (111)	Parallel	India	Adults with dyslipidaemia	Blood pressure, lipids, gluose	Pistachio nuts + TLC diet	40 g shelled	No nuts + TLC diet	I: 21 C: 21	12 weeks	High	NI
Liu (2018) (112)	Parallel	Korea	Young, healthy adults; age 20–39 years, BMI 17–30 kg/m	Blood pressure, lipids	Almonds, as snacks or pre-meal	56 g/day	High-carbohydrate foods, isocaloric	I: 57 C: 28	20 weeks	High	Industry
Madan (2021) (113)	Parallel	India	Adolescents and young adults with metabolic dysfunction (i.e. prediabetes or insulin resistance); age 16–25 years	Lipids, glucose, insulin resistance	Almonds	20 E%/56 g/day	Isocaloric Indian savory snacks	I: 107 C: 112	12 weeks	High	Industry
Njike (2015) (114)	Crossover	USA	Adults at high risk for diabetes (e.g. metabolic syndrome); mean age 54.9 years	Blood pressure, lipids, glucose, HbA1c	Walnuts with or without calorie-adjustment	56 g/day	No walnuts	97	6 months	Some concerns	Industry
Tey (2013) (115)	Parallel	New Zealand	Healthy, BMI ≥25 kg/m^2^, mean age 42.5 years	Blood pressure, lipids	Hazelnuts	30 g and 60 g	Habitual diet, no nuts	I 30 g: 33 I 60 g: 37 C: 37	12 weeks	Some concerns	NI
Torabian (2010) (116)	Crossover	USA	Healthy adults with normal to moderately high serum cholesterol; mean age 54 years	Lipids	Walnuts	12 E% (28–64 g/day)	Habitual diet	87	6 months	Some concerns	Industry
Wang, D (2021) (117)	Parallel	China	Subjects with or at risk for metabolic syndrome; mean age 46.2 years	Blood pressure, lipids, glucose	Peanuts	20 E%/56 g/day	White rice bars, isocaloric	I: 109 C: 100	12 weeks	Low	Mixed
Wang, J (2021) (118)	Parallel	USA	Healthy, BMI 27–35 kg/m; age 30–68 years.	Blood pressure, lipids	Mixed nuts (almonds, cashew nuts, hazelnuts, macadamia, pecan, pistachio, walnuts)	1.5 oz/d	Pretzel snack	I: 56 C: 39	24 weeks (12 weeks hypo-caloric, 12 weeks eucaloric)	High	Industry
Wang, X (2012) (119)	Parallel	China	Subjects with metabolic syndrome; age 25–65 years	Blood pressure, lipids, glucose	Pistachio nuts + AHA Step I diet	Recommended serving: 42 g/day Higher serving: 70 g/day	No pistachio nuts + AHA Step I diet	I 42 g: 27 I 70 g: 29 C: 30	12 weeks	Some concerns	Industry

1 Abbreviations: AHA, American Heart Association; C, control group; HbA1c, glyacted haemoglobin; HDL-C, high-density lipoprotein cholesterol; I, intervention group; TLC, therapeutic lifestyle changes.

Among the cohort studies, published between 1992 and 2021 (Table 3 and Supplementary Table 2), the total number of participants per study ranged from 1984 to 490,311 (median 35,988), providing 1,890,573 participants in the individual cohorts. Follow-up time ranged from 3.5 years up to 32 years. There were six reports including different exposures/outcomes from the Health Professionals’ Follow-up Study/Nurses’ Health studies (HPFS/NHS) (42, 73, 76, 77, 94, 100), four from the European EPIC study (36, 95, 101, 102), four from the Physicians’ Health Study (74, 83, 86, 90) and two each from the Atherosclerosis Risk in Communities (ARIC) (35, 85), EPIC-Potsdam (71, 82), the Netherlands Cohort (97, 98), Iowa Women’s Health (68, 78) and the Women’s Health Initiative studies (37, 72).

Five cohorts (in eight publications) included only females (37, 68, 70, 72, 76, 78, 88, 94) and two (in five publications) included only males (73, 74, 83, 86, 90). Most cohorts were from the US (10, 35, 37, 42, 43, 68, 72, 73, 75–78, 83, 85, 86, 88–90, 92, 94, 100), followed by Asia (43–45, 70, 81, 87, 93, 99) and Europe (36, 71, 79, 82, 84, 91, 95, 97, 98, 101, 102), while one cohort was Australian (80) and 1 included 16 countries from all 5 continents (96). The nuts or seeds examined were usually unspecified (here designated ‘total nuts’, including seeds and nut butter) while peanuts and/or walnuts were separately reported in seven studies (42, 44, 70, 81, 87, 92, 94, 97). Seeds were explicitly grouped with nuts in only four studies (36, 37, 45, 75, 95, 101, 102). Intake was assessed by food frequency questionnaires in all studies, and only at baseline in all but six cohorts (35, 42, 70, 73, 76, 77, 84, 85, 92–94, 100). Most studies adjusted for intake of other food groups while six also adjusted for predefined diet quality scores (79, 80, 84, 88, 89, 100). Several studies adjusted for risk factors that are possibly mediators of the associations between nut consumption and CVD, including hypercholesterolaemia and hypertension. The populations had in general a low consumption; we calculated the intake of total nuts as median 4 g/day. All meta-analyses had total nuts and seeds (i.e. unspecified) as exposure. Due to few studies of individual types of nuts/seeds, only peanuts were assessed separately, in one meta-analysis of CVD. All cohort studies reported public funding, but a few were also funded by industry grants (76, 84, 94, 96).

The RCTs, all published between 2010 and 2021, analysed between 42 and 625 participants each, for a total N of 2,336 (study characteristics in Table 4). No RCT reported disease incidence. The RCTs used almonds (*n* = 4), hazelnuts (*n* = 1) peanuts (*n* = 2), pistachio nuts (*n* = 4) and walnuts (*n* = 5) while two trials used a mixed nuts intervention. Median duration was 12 weeks (range from 12 weeks and 2 years). Thirteen trials had a parallel design while five were crossover trials [of the latter, Njike et al. (114) used a Latin square design with both a parallel and cross-over design]. The trials were performed in Australia (104, 107), New Zealand (115), China (117, 119), India (34, 111, 113), Korea (110, 112), Spain (106, 108), Turkey (105), and the US (109, 114, 116, 118), while one study included both Spanish and US participants (103). According to the authors’ descriptions, the participants were generally healthy (103, 104, 107, 109, 112, 115, 116) or were diagnosed having dyslipidemia (105, 111), the metabolic syndrome (34, 106, 110, 114, 117, 119) or prediabetes (108, 113). Mean BMI ranged from 22.3 to 33.4 kg/m^2^ (median 30 kg/m^2^) and was ≥30 kg/m^2^ in nine trials (34, 104–107, 109, 113–115).

In one study, the intervention group was instructed to substitute nuts for other foods, while others were only given general dietary advice or maintained their habitual diet. The control groups were usually only instructed to avoid nuts and otherwise follow similar dietary guidelines as the intervention groups. In some trials, the control groups were provided with iso-caloric carbohydrate-rich or savory snacks (107, 112, 113, 117, 118), while two replaced nuts with either fat-rich foods (108) or with white bread as control (110). Due to our eligibility criteria, no groups were on hypocaloric diets, but in one study with four arms, two of the groups were instructed to adjust calorie intake while the others consumed their diets ad libitum (114). Changes in total energy intake was often not reported, but increased significantly in the nut groups in five trials (103, 104, 109, 115, 116). Where reported, there were no significant effects on mean body weight between the intervention and control groups (34, 105–109, 111–115, 117–119) except for a 0.5 [standard error (SEM) 0.2] kg increase in the peanut versus control group in one trial (104). Most RCTs were funded by industry while three had mixed funding (104, 106, 117), one reported only public funding (105) and one had no information (115).

### Risk of bias assessment

**Fig. 2:**
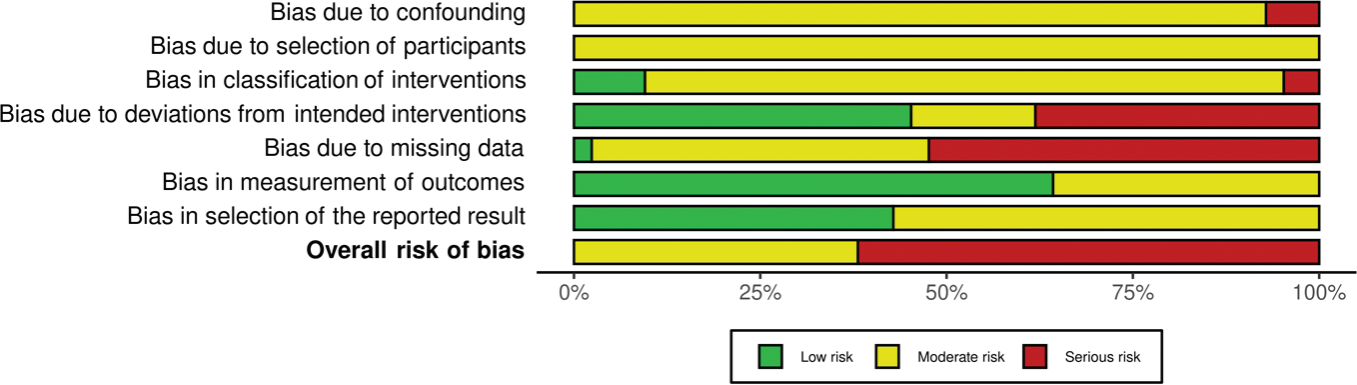
Summary risk of bias per domain in cohort studies.

**Fig. 3:**
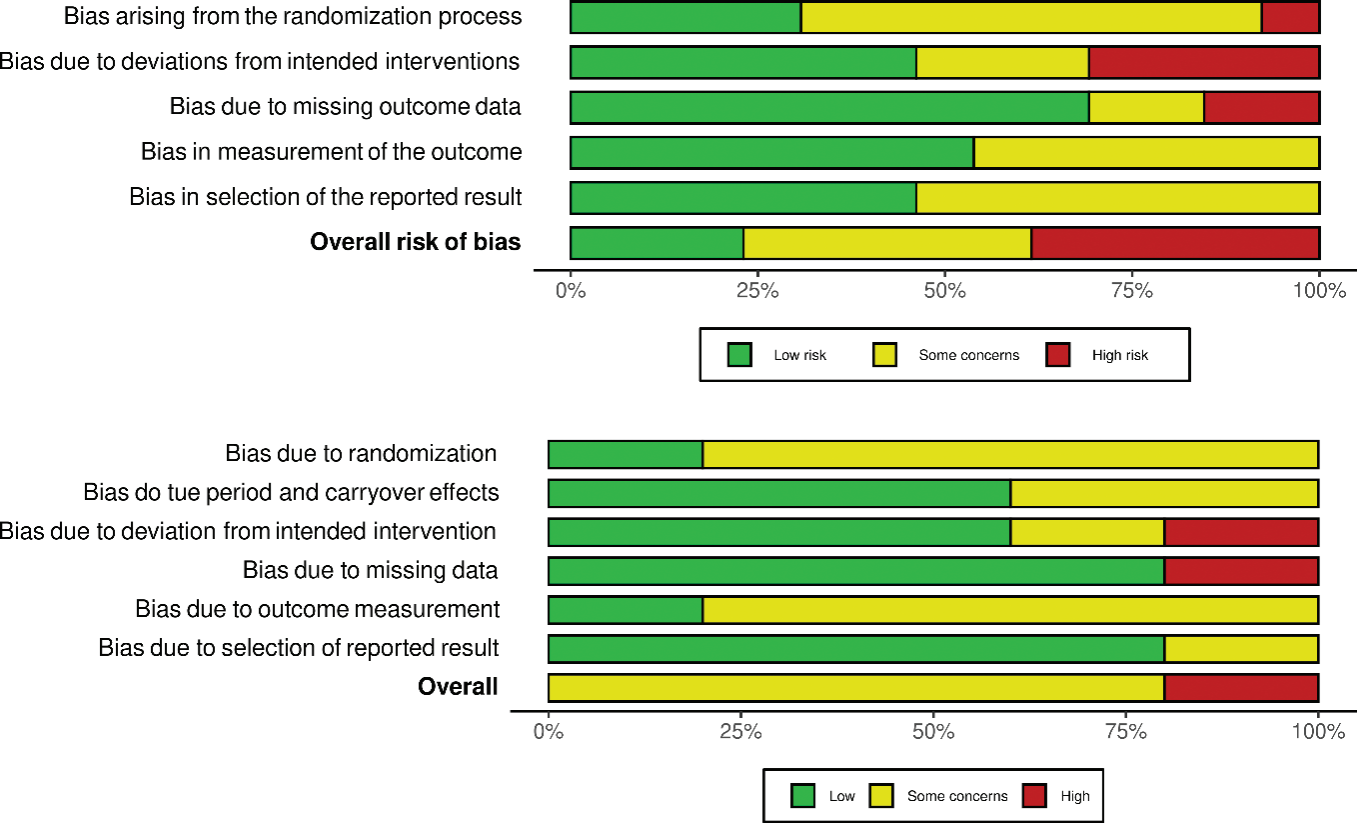
Summary risk of bias per domain in randomized controlled trials. Top: parallell studies, bottom: crossover studies.

The summary RoB assessments of the included studies are shown in Figs. 2 and 3, and on study level in Supplementary Figs. 1 and 2. Due to the possibility of confounding and selection bias, no cohort study had a low RoB in all domains, and hence no low overall RoB. None had critical RoB in any domain.

Among the RCTs, most had a low RoB in the domains of missing outcome data, deviations from the intended interventions and selective reporting. Only three were rated with a low overall RoB. The most frequent reason for rating RoB as ‘high’ was due high rates of drop-outs while missing intention-to-treat (ITT) analysis (110–112, 118).

### Synthesis of results

**Fig. 4:**
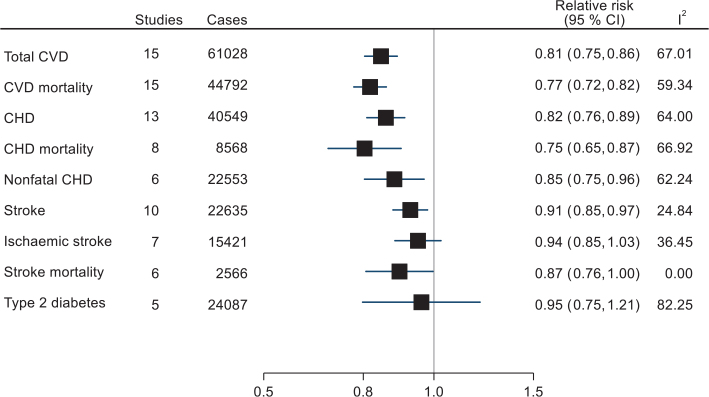
Summary forest plot of pooled relative risk estimates for associations between high versus low total nuts/seeds consumption and risk of cardiometabolic disease. Meta-analyses were performed with random-effects restricted maximum likelihood models. *I*^2^ = heterogeneity (%).

Of the cohort studies, 23 (32 reports) were included in at least one meta-analysis on CVD, CVD mortality, CHD, CHD mortality, stroke, stroke mortality, ischaemic stroke and T2D (10, 35–37, 42–45, 68, 71, 74, 75, 78, 80–91, 93–97, 99, 101). The total number of participants per meta-analysis was 1,295,163 for overall CVD, 1,186,541 for CHD, 1,081,742 for stroke and 211,091 for T2D. Two reports involved a sub-cohort of the multinational EPIC study, and were therefore excluded from meta-analyses that already included the overall EPIC cohort reporting on the same outcome (71, 82). All study results are presented in detail Supplementary Tables 3–6. Figure 4 is a summary forest plot of all the outcomes meta-analysed where high versus low consumption were compared. Inverse associations were reported for all the outcomes, while the upper CI included 1.00 for stroke mortality, ischemic stroke and T2D. The forest plots of study-specific and overall effect sizes per outcome are shown in Supplementary Fig. 3A-I.

All 18 RCTs were included in meta-analyses of total and LDL-C, systolic and diastolic blood pressure (DBP). Results for fasting glucose, HbA1c and HOMA-IR are presented in Supplementary Tables 9–11.

### Total nuts/seeds and cardiovascular disease

The meta-analysis of high versus low consumption of total nuts and total CVD included 15 studies (61,028 incidents or deaths) (Table 5 and supplementary Fig. 3A). The summary RR was significantly lower in the high versus low consumers; RR 0.81 (95% CI 0.75, 0.86, *P* < 0.0001), although there was substantial heterogeneity (*I*^2^ = 67%, *p*_heterogeneity_ < 0.0001). Excluding one study at the time did not appreciably modify the result (RR range from 0.79 to 0.83) (Supplementary Fig. 4A). Subgroup analyses (shown in Supplementary Table 12) revealed only minor non-significant differences by region, sex, duration of follow-up, RoB and adjustment for cholesterol/hypercholesterolaemia or hypertension. Mean/median age was not associated with the effect size.

**Table 5:** Summary results from meta-analysis of cohort studies^1^

Outcome	*N* studies	Median follow-up time (years)	*N* cases	Relative risk (95% CI) High versus low consumption	Heterogeneity (*I*^2^, *P*)
Total nuts					
CVD	15	11.2	61,028	0.81 (0.75, 0.86)	67.0%, *P* < 0.001
CVD mortality	15	11.2	44,792	0.77 (0.72, 0.82)	59.3%, *P* < 0.001
CHD	13	15	40,549	0.82 (0.76, 0.89)	64.0%, *P* < 0.01
CHD mortality	8	15	8,568	0.75 (0.65, 0.87)	66.9%, *P* < 0.01
Nonfatal CHD	6	13.25	22,553	0.85 (0.75, 0.96)	62.2%, *P* < 0.01
Stroke	10	12.7	22,635	0.91 (0.85, 0.97)	24.8%, *P* = 0.349
Ischaemic stroke	7	17	15,421	0.94 (0.85, 1.03)	36.5%, *P* = 0.247
Stroke mortality	6	9.8	2,566	0.87 (0.76, 1.00)	0.0%, *P* = 0.599
T2D	5	12	24,087	0.95 (0.75, 1.21)	82.25%, *P* = 0.003
Peanuts					
CVD	5	14	25,834	0.83 (0.79, 0.88)	36.0%, *P* = 0.23

1 Abbreviations: CHD, coronary heart disease; CVD, cardiovascular disease; T2D, type 2 diabetes.

**Fig. 5:**
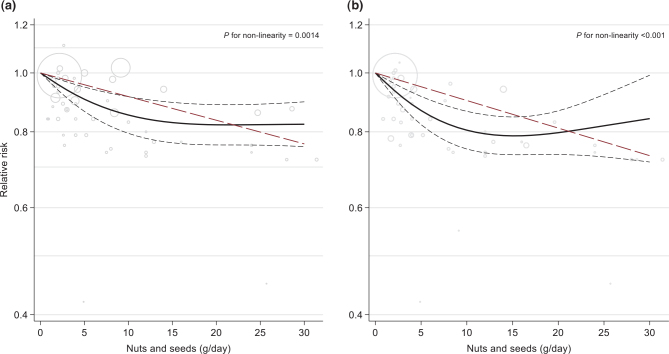
Linear (red, dashed line) and non-linear dose-response (black lines with confidence intervals) association between total nuts and seeds consumption and risk of total cardiovascular disease (panel A; 16 studies) and cardiovascular disease mortaltiy (panel B; 15 studies) in cohort studies, with 0 g/day as reference. Circles show the effect estimates for each level of intake in the individual studies, weighted by the inverse of the standard errors. Vertical axes are log scaled.

In the dose-response analysis, one additional study was included (71). Assuming a linear dose-response, the RR for total CVD was 0.76 (95% CI 0.68, 0.86, *P* < 0.0001) per 30 g/day, while the non-linear analysis suggested a levelling off at 17 g/day (RR (95% CI) per 30 g/day = 0.82 (0.76, 0.90), *P* for non-linearity = 0.0014) (Fig. 5A).

Focusing on CVD deaths alone (15 studies with 44,792 cases), the corresponding RR comparing the high versus low consumption categories of total nuts was 0.77 (95% CI 0.72, 0.82, *P* < 0.0001), with high heterogeneity (*I*^2^ = 59.34%, *p*_heterogeneity_ < 0.0001). Omitting Sun et al. from this analysis removed most of the heterogeneity and yielded only a slightly different effect estimate; RR 0.74 (95% CI 0.71, 0.78, *I*^2^ = 16.07, *p*_heterogeneity_ = 0.36) (Supplementary Fig. 4B). Again, there were no significant subgroup differences or modification by age (Supplementary Table 13). The RR per 30 g/day was 0.73 (95% CI 0.67, 0.80, *P* < 0.0001) in the linear dose-response analysis (15 studies), and 0.84 (0.71, 0.99) per the non-linear analysis (*P* for non-linearity < 0.001) (Fig. 5B).

One study was not included in the meta-analyses as the results were only reported for nut consumers (defined as ≥2 servings/month) versus non-consumers (79). Nut consumers had a HR for CVD mortality of 0.87 (95% CI: 0.57, 1.32) during a median follow-up time of 4.3 years.

### Total nuts/seeds and coronary heart disease

**Fig. 6:**
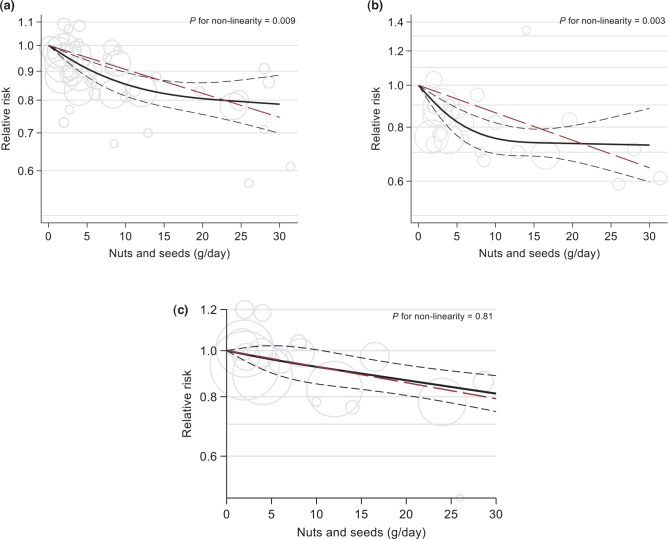
Linear (red, dashed line) and non-linear dose-response (black lines with confidence intervals) association between total nuts and seeds consumption and risk of total coronary heart disase (panel A; 14 studies), coronary heart disease mortality (panel B; 9 studies) and nonfatal coronary heart disase (panel C; 6 studies) in cohort studies, with 0 g/day as reference. Circles show the effect estimates for each level of intake in the individual studies, weighted by the inverse of the standard errors. Vertical axes are log scaled.

Incidence or deaths from CHD was reported in 18 publications (10, 35, 42, 43, 73, 74, 76, 78, 80, 86, 87, 89, 91, 93, 95–97, 99). Of these, 13 studies (encompassing 40,549 events) were included in a meta-analysis, suggesting a significantly reduced risk associated with high versus low nut consumption (RR 0.82, 95% CI 0.76, 0.89, *P* < 0.0001; (*I*^2^ = 64.0%, *p*_heterogeneity_ = 0.0014) (Table 5 and supplementary Fig. 3C). Some heterogeneity was explained by Fraser et al. (10), but the summary RR and significance level were practically unchanged by excluding this in a sensitivity analysis (RR = 0.84, 95% CI 0.79, 0.90) (Supplementary Fig. 4C). The inverse estimate was more pronounced in studies from the US (RR 0.76, 95% CI 0.67, 0.85), but there were few studies from other regions. Studies with <10 years of follow-up did show a stronger association (RR 0.71, 95% CI 0.59, 0.85) than those with ≥10 years of follow-up (*P* = 0.04) but there was no significant linear association between years of follow-up and the effect size. Other subgroup differences were not found (Supplementary Table 14). In dose-response analysis of 14 studies (40,904 events), the summary RR (95% CI) per 30 g/day was 0.75 (0.68, 0.82) in the linear and 0.79 (0.70, 0.89) in the non-linear assessment (Fig. 6A).

For CHD mortality (8 studies with 8,568 deaths) there was a stronger association between high versus low nut consumption, RR 0.75 (95% CI 0.65, 0.87, *P* < 0.001; *I*^2^ = 66.92, *p*_heterogeneity_ = 0.008) (Table 5 and Supplementary Fig. 3D). Excluding the study by Larsson et al. (91), which showed an opposite trend, had little effect on the summary effect estimate (RR 0.72, 95% CI 0.64, 0.93) (Supplementary Fig. 4D). One study that was not included in the meta-analysis found a HR of 0.74 (95% CI 0.38, 1.45) between consumption of ≥2 servings/month versus no consumption (79). A dose-response analysis of nine studies found a pooled RR of 0.64 (95% CI 0.58, 0.72) per 30 g/day in the linear analysis, but there was evidence (*P* = 0.003) of a non-linear association, indicating a flattening at intakes ≥18 g/day (RR 0.73, 95% CI 0.60–0.88) (Fig. 6B).

Restricting the outcome to only non-fatal CHD events (six studies with in total 22,553 events) also showed a significant association (high vs. low RR = 0.85, 95% CI 0.75, 0.96, *P* = 0.009; *I*^2^ = 62.24, *p*_heterogeneity_ = 0.008). The RR per 30 g/day was 0.79 (95% CI 0.70, 0.89), and this association did not appear to be non-linear (*P* for non-linearity = 0.8095) (Fig. 6C).

### Total nuts/seeds and stroke

For stroke (10 studies with 22,635 incident events or deaths), high versus low consumption of total nuts was associated with a small reduction in total stroke (summary RR 0.91, 95% CI 0.85, 0.97, *P* = 0.007) (Table 5 and Supplementary Fig. 3F). There was no significant heterogeneity (*I*^2^ = 24.84%, *P* = 0.349), but in a sensitivity analysis excluding Tong et al., the largest study, the 95% CI would include 1 (Supplementary Fig. 4F). There were no significant differences regarding follow-up time, region, sex, RoB or adjustment for risk factors (Supplementary Table 15). The association was marginally stronger for stroke mortality (six studies; RR 0.87, 95% CI 0.76, 1.00, *P* = 0.044; *I*^2^ = 0%, *p*_hetereogeneity_ = 0.60) (Supplementary Figs. 3G and 4G). In the only study that was not included in the meta-analysis, there was no association for stroke mortality (HR 0.98, 95% CI0.36–2.66 per ≥2 servings/month versus none). For ischaemic stroke, the summary result was not significant (summary RR 0.94, 95% CI 0.85, 1.03, *P* = 0.171; *I*^2^ = 36.45, *p*_heterogeneity_ = 0.247) in seven studies (Supplementary Figs. 3H and 4H).

**Fig. 7:**
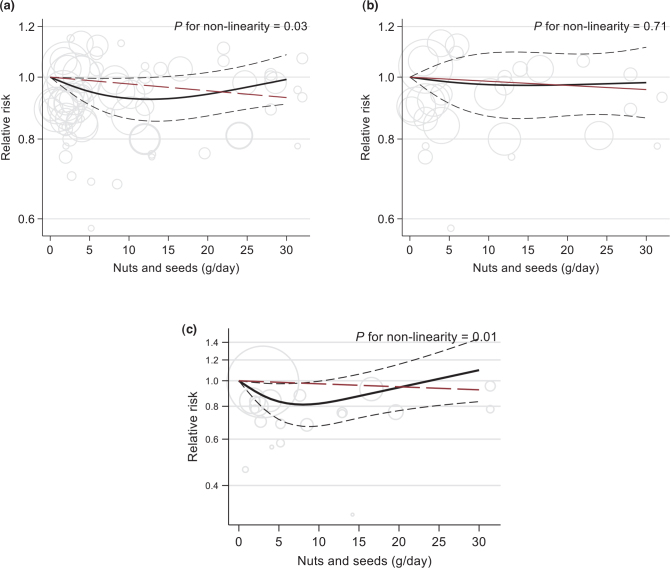
Linear (red, dashed line) and non-linear dose-response (black lines with confidence intervals) association between total nuts and seeds consumption and risk of total stroke (panel A; 11 studies), ischaemic stroke (panel B; 8 studies) and stroke mortality (panel C; 6 studies) in cohort studies, with 0 g/day as reference. Circles show the effect estimates for each level of intake in the individual studies, weighted by the inverse of the standard errors. Vertical axes are log scaled.

Among 11 studies included in dose-response analyses, there was a non-significant dose-response relationship between total nuts and stroke [per 30 g/day, RR 0.93 (95% CI 0.83–1.04, *P* = 0.19)]. The non-linear analysis indicated a somewhat U-shaped association, with a nadir between 11 and 14 g/day and a null association at 30 g/day (RR 0.99, 95% CI 0.91, 1.08) (Fig. 7A). Similar lack of any dose-response association was found for ischaemic stroke (RR 0.96, 95% CI 0.82, 1.13 per 30 g/day, *P* = 0.664) (Fig. 7B), while for stroke mortality the association appeared to be non-linear and J-shaped (*P* for non-linearity = 0.01) with a lower RR up to 7–9 g/day (RR 0.81), but the CI were wide (RR per 30 g/day = 1.10 (0.83, 1.45)) (Fig. 7C).

### Total nuts/seeds and type 2 diabetes

**Fig. 8:**
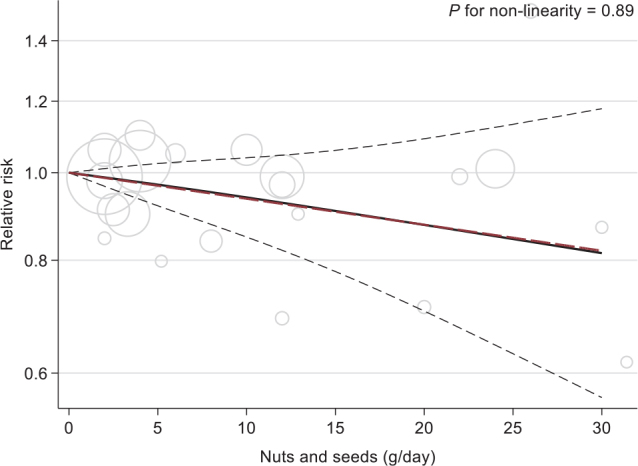
Linear (red, dashed line) and non-linear dose-response (black lines with confidence intervals) association between total nuts and seeds consumption and risk of type 2 diabetes in cohort studies (five studies), with 0 g/day as reference. Circles show the effect estimates for each level of intake in the individual studies, weighted by the inverse of the standard errors. Vertical axis is log scaled.

For high versus low consumption of total nuts, five studies of T2D (24,389 incident cases) were pooled (45, 68, 90, 94, 101). The summary RR between the high versus low intake categories was 0.95 (95% CI 0.75, 1.21, *P* = 0.69), and the heterogeneity was considerable (*I*^2^ = 82.25%, *p*_heterogeneity_ = 0.003) (Table 5 and Supplementary Fig. 3I). In particular, the study by Parker et al. (68) found a harmful association, and excluding this would yield a RR of 0.88 (95% CI 0.75, 1.03; *I*^2^ = 55.63, *p*_hetereogeneity_ = 0.08) (Supplementary Fig. 4I). There was no significant dose-response association and no evidence of non-linearity (Fig. 8).

Two additional studies assessed T2D mortality alone. Neither Luu et al. nor Amba et al. found any overall association with nut/peanut consumption (43, 75).

### Types of nuts or seeds

Associations between peanut consumption and overall CVD were reported in five cohorts with 27,512 cases (42–44, 87, 97) (Supplementary Fig. 5A). The summary RR for CVD was 0.83 (95% CI: 0.79, 0.88) in the highest category (median 4.4 g/day), with a modest degree of heterogeneity (*I*^2^ = 35.99%, *P* = 0.23). Considering the limited range of consumption, the dose-response analysis (*P* for non-linearity = 0.0228) indicated a significant risk reduction up to ~3 g/day (RR 0.84, 95% CI: 0.77, 0.92), and no significant association from ≥8 g/day (Supplementary Fig. 5B).

Due to few studies, no other meta-analyses of specific nuts or seeds were performed. However, four studies reported associations between peanut consumption and risk of CHD and stroke (42, 43, 87, 97), of which three reported a significantly reduced risk of CHD in the highest consumption category (42, 43, 97), while all four found a significantly reduced risk (from 10 to 29% reduction) of stroke. Villegas et al. (70) found a lower risk of T2D with higher intakes of peanuts, but two other studies found no significant associations (43, 94).

Walnuts were reported in only two cohorts for CVD, that is, the HPFS/NHS (42, 92) and PREvención con DIeta MEDiterránea trial (PREDIMED) (84) and only in the HPFS/NHS for CHD, stroke and T2D (42, 94). For all endpoints, walnut intake was significantly, inversely associated with risk.

Regarding peanut butter, no studies reported any significant associations with overall CVD (42, 75, 97), CHD (42, 97), stroke (42, 89, 97) or T2D (68), although several other studies included peanut butter in the definition of total nuts. No studies reported separate results for seeds alone.

### Replacement of other foods with nuts

**Fig. 9:**
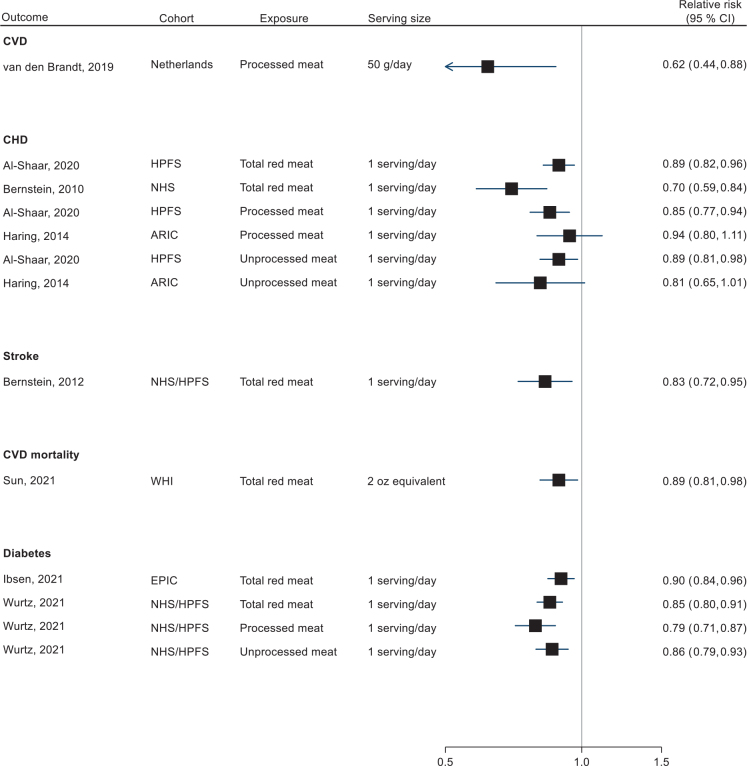
Associations of nuts versus meat consumption and cardiometablic endpoints from substitution models in cohort studies.

Hypothetical substitutions of nuts for other dietary protein sources (mainly red meat) were reported in eight reports from five different cohorts (35, 37, 73, 76, 77, 98, 100, 102). The results for replacing total/processed red meat with nuts is illustrated without meta-analysis in Fig. 9.

Replacing 1 serving/day of total red meat with nuts was associated with a lower risk of CHD and stroke in the HPFS and NHS (73, 76, 77), CVD mortality in the Women’s Health Initiative cohort study (37) and T2D in the EPIC-InterAct and the HPFS/NHS (100, 102). Regarding processed meat, van den Brandt also found a 38% lower risk of CVD per 50 g/day replacement (98).

Replacement of both processed and unprocessed meat with nuts was associated with a lower risk of CHD in the HPFS (by 15 and 11% per serving/day, respectively) (73), but not in ARIC (35). There were also no significant associations between replacement of other protein sources with nuts and CHD in the ARIC study (35), while Sun et al. found a significantly lower risk of CVD mortality per 2 oz-equivalent of nuts and seeds compared with both eggs (HR = 0.44), dairy products (HR = 0.81) and legumes (HR = 0.70) (37).

### Effects of nuts on blood lipids

In total, 17 RCTs on TC and 16 RCTs of LDL-cholesterol (LDL-C) were included in meta-analyses, with 1,710 and 1,602 participants, respectively. One of these studies included two comparisons, one with and one without energy-controlled diets.

**Fig. 10:**
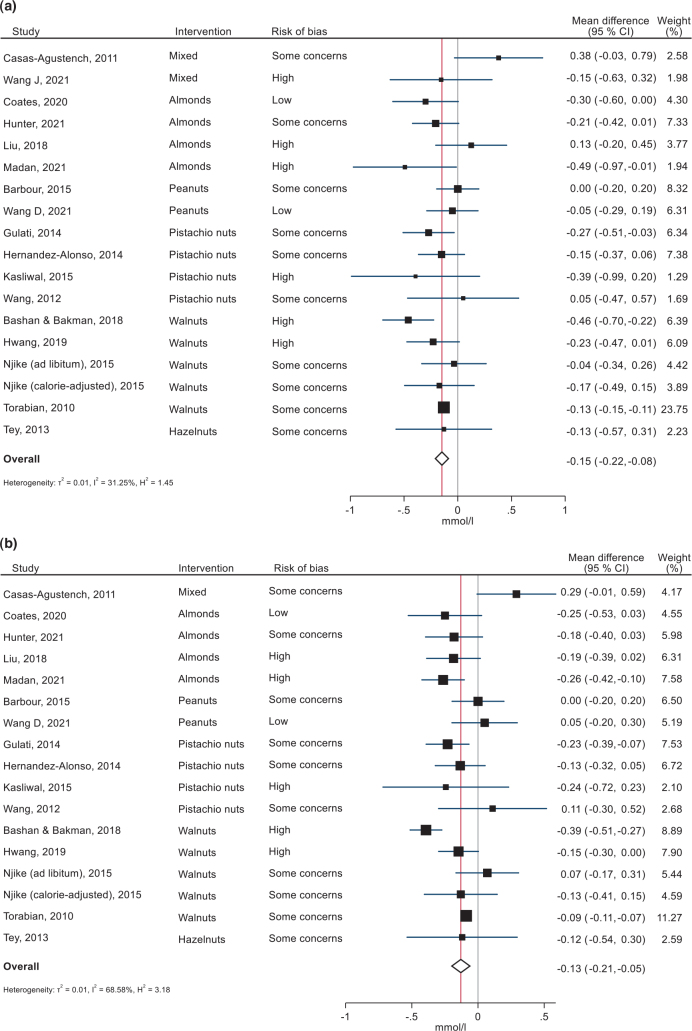
Forest plots of the effects of nut consumption on total cholesterol (A) and LDL-cholesterol (B) in randomized controlled trials, sorted by type of nut intervention. Effect sizes are weighted mean differences with 95% confidence intervals, in mmol/L. Analyses were performed with random-effects restricted maximum likelihood models.

Median TC level in the studies was 5.2 mmol/L (range 3.75–6.5 mmol/L). The summary mean difference at follow-up between the nut interventions and control groups was −0.15 (95% CI −0.22, −0.08, *P* < 0.0001) mmol/L (Fig. 10A). There was little heterogeneity between the studies (*I*^2^ = 31.25%, *P* = 0.086). Sensitivity analyses excluding one study one-by-one did not affect the overall effect estimate (Supplementary Fig. 4J). Excluding studies with a high overall RoB (105, 110–113, 118) changed the overall effect modestly (MD −0.13, 95% CI: −0.15, −0.11) but removed all between-study heterogeneity.

Subgroup analyses (Supplementary Table 16) indicated a significant effect of specifically pistachio and walnuts (mean difference 0.20 mmol/L for both). Only two studies used mixed nuts (106, 118), both finding no significant effects. The effect was larger in two studies including patients with dyslipidemia, with an overall mean difference of −0.45 (95% CI: −0.67, −0.23), but there were no significant association with baseline TC or differences between studies with a mean baseline TC above versus below the median. There was no significant modification by study duration (continuously or 12 vs. ≥12 weeks) nor by amount of nuts or age.

In the studies assessing LDL-C, median baseline LDL-C was 3.26 mmol/L. Nut consumption lowered LDL-C by 0.13 mmol/L (95 CI −0.21 to −0.05, *P* < 0.0001) compared to no nuts (Fig. 10B). Heterogeneity in the result was substantial (*I*^2^ = 68.58%, *P* < 0.001). Sensitivity analyses excluding one study one-by-one did not affect the overall effect estimate (Supplementary Fig. 4K).

The effect was significant for almonds (four studies), pistachios (four studies) and walnuts (five studies), but not for hazelnuts (one study), mixed nuts (one study) or peanuts (two studies); *P* for group differences = 0.01 (Supplementary Table 17). In particular, almond interventions were associated with a mean difference of −0.22 (−0.33, −0.12) mmol/L. Study duration, baseline LDL-C, mean BMI, age or the amount of nuts did not modify the effect or explain the heterogeneity. Studies with high RoB showed a larger effect (*P* for group differences = 0.02), excluding those reduced the mean difference to −0.09 (95% CI: −0.11, −0.07). The effect also varied by type of participants, being stronger in studies of subjects characterized with dyslipidaemia (−0.38 (−0.50, −0.27) mmol/L) and prediabetes (−0.21 (−0.33, −0.08) mmol/L), but those studies also had a high RoB.

### Effects of nuts on blood pressure

**Fig. 11:**
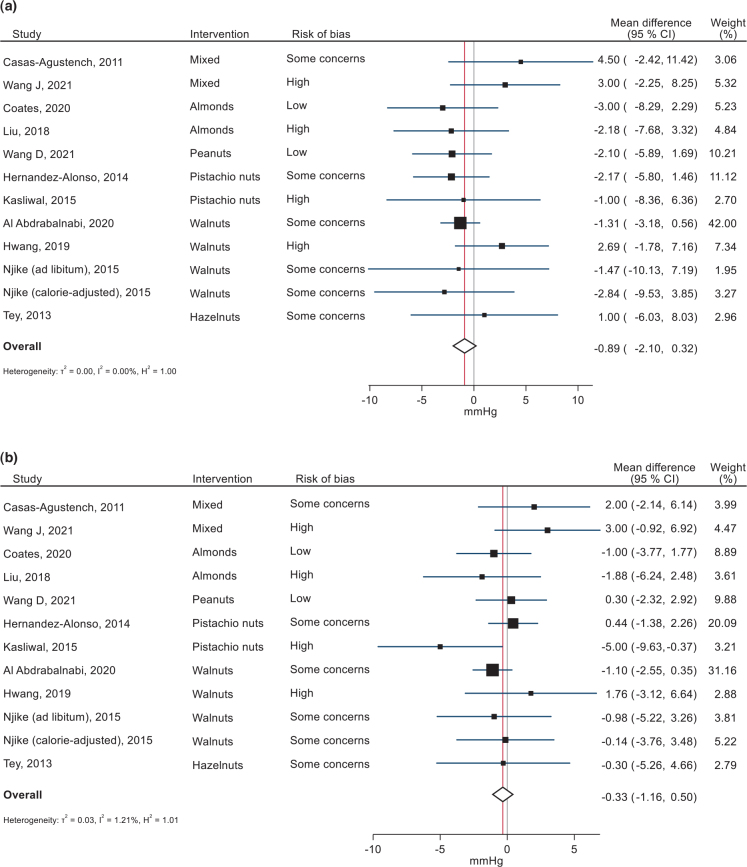
Forest plots of the effects of nut consumption on systolic (A) and diastolic (B) blood pressure in randomized controlled trials, sorted by type of nut intervention. Effect sizes are weighted mean differences with 95% confidence intervals, in mmHg. Analyses were performed with random-effects restricted maximum likelihood models.

Effects on systolic and DBP were assessed in 11 RCTs (103, 106–108, 110–112, 114, 115, 117, 118) including a total of 1,568 participants. Overall, nut consumption (range from 30 to 57 g/day) had no significant effect on either measure: −0.89 (95% CI: −2.10, 0.32) mmHg for SBP, −0.33 (−1.16, 0.50) mmHg for DBP (Fig. 11A and B). There was no significant heterogeneity in the results (*I*^2^ = 0%, *P* = 0.583 for SBP, *I*^2^ = 1.0%, *P* = 0.409 for DBP). The effect did not significantly differ by type of nut consumed, study duration, participant characteristics (including age and baseline SBP or DBP) or study RoB (Supplementary Tables 18 and 19). However, for SBP, higher doses were associated with larger effects, in favour of nuts. A post hoc analysis excluding trials using less than the median dose of nuts (54 g) did find a significant effect on SBP (six trials; MD −2.29 (95% CI −4.29, −0.29), *I*^2^ = 0% *p*_heterogeneity_ = 1.0). This was not the case for DBP.

### Effects on glycaemic indices

The results for fasting glucose, HbA1c and insulin resistance are presented in Supplementary Tables 9–11. We did not perform meta-analyses on these outcomes. Fasting glucose was assessed in 13 RCTs (34, 103, 104, 106–111, 114, 117, 119), and the results appeared mixed. Of the three studies of almonds, one found a significantly higher fasting glucose in the intervention group (113). Participants in this trial had insulin resistance, but not impaired fasting glucose. Two of four trials of pistachio nuts found a significant glucose lowering by nuts, while there was a small increase in one study of walnuts, but the other walnut studies also found opposite and null effects. Only one trial used mixed nuts (30 g/day), finding no effect (106). Six trials also reported effects on HbA1c (34, 108–110, 114), in which the mean baseline level was about 5.6%. In one almond trial with subjects with insulin resistance (113), a significant reduction in HbA1c was found, while the other trials reported no significant differences between the groups.

Finally, insulin resistance, as HOMA-IR, was reported in six trials (104, 106–109, 113). A significant effect was found in one study of pistachio nuts (56 g/day) among persons with prediabetes, where HOMA-IR was reduced by −0.69 (95% CI −1.07, −0.31) in the pistachio arm and increased by 0.97 (95% CI 0.49, 1.44) in the control arm (108). One study also found and effect with 30 g/day of mixed nuts among persons with metabolic syndrome, with a mean difference between the nut versus control group of −0.67 (95% CI −1.24, −0.11), adjusted for weight change (106).

### Publication bias

Based on inspection of funnel plots, shown in Supplementary Fig. 6A-H, and Egger’s test, we did not find evidence of publication bias in the form of small study-effects bias for total nuts and CVD (*P* = 0.216), CVD mortality (*P* = 0.115), CHD (*P* = 0.512) or stroke (*P* = 0.33). The tests were also insignificant in the meta-analyses of RCTs (total-C: *P* = 0.94, LDL-C: *P* = 0.2, SBP: *P* = 0.46, DBP: *P* = 0.74). Therefore, no adjustment for publication bias was approached.

### Certainty in the evidence

Table 6 shows a summary of findings on nuts and seeds consumption and risk of CVD, CHD, stroke and T2D, with the grading of the strength of evidence for these outcomes.

**Table 6:** Summary of outcomes and strength of evidence^1^

Outcome	Exposure/intervention	N studies	Association (direction per study)^2^	Meta-analysis results, high versus low consumption, RR (95% CI)	Heterogeneity (*I*^2^)^3^ (%)	RoB	Dose-response	Strength of evidence
CVD (37, 42–44, 71, 75, 78–81, 84, 86, 88, 89, 93, 96–98)	Total nuts	17	↑ 0 ↓ 10 ↔ 7	(15 studies): 0.81 (0.75, 0.86)	67.01	Moderate: 7 Serious: 8	Yes	Probable
CVD mortality (37, 42–44, 75, 78–81, 84, 86, 88, 89, 93, 96, 97)	Total nuts	16	↑ 0 ↓ 11 ↔ 5	(15 studies): 0.77 (0.72, 0.82)	59.34	Moderate: 8 Serious: 7	Yes	Probable
CHD (10, 35, 42, 43, 73, 74, 76, 78–80, 89, 91, 93, 95–97, 99)	Total nuts	15	↑ 0 ↓ 6 ↔ 9	(13 studies): 0.82 (0.76, 0.89)	64.00	Moderate: 6 Serious: 7	Yes	Probable
CHD mortality (10, 42, 43, 74, 76, 78–80, 91, 97, 99)	Total nuts	10	↑ 0 ↓ 6 ↔ 4	(8 studies): 0.75 (0.65, 0.87)	66.92	Moderate: 4 Serious: 4	Yes	Probable
Nonfatal CHD (10, 42, 74, 76, 89, 91, 96)	Total nuts	6	↑ 0 ↓ 3 ↔ 3	(6 studies): 0.85 (0.75, 0.96)	62.24	Moderate: 2 Serious: 4	Yes	Probable
Stroke (36, 42, 43, 72, 77, 82, 83, 85, 86, 89, 96, 97, 99)	Total nuts	12	↑ 0 ↓ 2 ↔ 10	(10 studies): 0.91 (0.85, 0.97)	24.84	Moderate: 5 Serious: 5	Non-linear	Suggestive, limited (no association)
Ischaemic stroke (36, 42, 43, 72, 83, 85, 89, 91)	Total nuts	8	↑ 0 ↓ 1 ↔ 7	(7 studies): 0.94 (0.85, 1.03)	36.45	Moderate: 3 Serious: 4	No	Suggestive, limited (no association)
Stroke mortality (42, 43, 79, 80, 82, 86, 97, 99)	Total nuts	8	↑ 0 ↓ 0 ↔ 8	(6 studies): 0.87 (0.76, 1.00)	0.00	Moderate: 2 Serious: 4	Non-linear	Suggestive, limited (no association)
T2D (45, 68, 71, 90, 94, 100–102)	Total nuts	6	↑ 1 ↓ 1 ↔ 3	(5 studies): 0.95 (0.75, 1.21)	82.25	Moderate: 0 Serious: 5	No	Suggestive, no conclusion

1 Abbreviations: CHD, coronary heart disease; CVD, cardiovascular disease; T2D, type 2 diabetes.

2 Arrows indicate higher risk (↑), lower risk (↓) or no association (↔)

We rated the association between nuts and seeds consumption and CVD and CHD as *probably* causal, based on consistent evidence from several cohort studies, in diverse settings, including a large number of participants giving relatively high precision. Although there were some inconsistencies between studies, the variations in results appeared to be more related to the magnitude rather than direction of associations. Moreover, the confidence was strengthened by the existence of a dose-response gradient. No study had low RoB, due to the inherent potential for confounding in observational studies. However, the lowering effects on blood lipids in the RCTs give partial mechanistic support for such associations, given the aetiology of CHD and the nutritional composition of nuts and seeds that may to some extent account for the effects. Still, the lack of RCTs on clinical outcomes precluded a judgment of a convincing effect.

Regarding stroke, there was *limited, suggestive* evidence for lack of a causal association. The meta-analyses did suggest a small inverse association between nuts/seeds and both total stroke and stroke mortality, and a potentially non-linear dose-response association, but the effect sizes may be too small to justify a recommendation and the null associations seemed to be generally consistent. However, the number of cases was often small in the studies showing non-significant associations, which may have increased the imprecision.

For T2D, the evidence was *limited* and *no conclusion* could be made, as the results of the cohorts were highly inconsistent and no dose-response relationship was detected. The results were also to some extent influenced by one study. There were also relatively few studies on this outcome, which hampered exploration of sources of heterogeneity, implying that the effect estimate and the certainty may change with further studies. Likewise, the RCTs on T2D biomarkers, although no meta-analyses could be performed, did not lend support in strengthening the certainty.

The evidence was too limited for any conclusion concerning individual types of nuts.

## Discussion

This comprehensive systematic review with meta-analyses of both observational and intervention studies adds to the evidence of lower risk of CVD in association with higher consumption of nuts/seeds based on prospective cohort studies mostly conducted in low-consuming populations. Comparing the highest with the lowest category of consumption, we found a 19% lower risk of overall CVD and a 23% lower risk of CVD mortality among high consumers. The associations appeared to be driven by reduced risk of CHD, especially CHD mortality (25% lower risk in high vs. low consumers). This was further supported by evidence for dose-response relationships, which appeared to be non-linear and especially related to increments in nut intakes well below 30 g/day. The curves appeared to level off at about 17–18 g/day, but this must be interpreted cautiously due to limited data at high intakes. Acknowledging the lack of RCTs of nut consumption and CVD/CHD, but lipid-lowering effects of nuts found in the included RCTs, we considered the evidence for a causal relationship as *probable.* In contrast, we are less confident in the effect of nut consumption on risk of stroke and T2D, for which the associations were smaller and largely insignificant. We did not find significant effects on blood pressure, while findings on fasting blood glucose, HbA1c and insulin resistance (HOMA-IR) appeared inconsistent when evaluated qualitatively. Of individual types of nuts, peanuts were associated with a significantly lower risk of CVD, while there was insufficient data to conclude on peanuts or other specific nuts/seeds and other outcomes than CVD. While it was not possible to separate nuts from seeds in the body of cohort studies, all RCTs were based on nuts alone.

No RCTs testing directly the effect of nuts on CVD endpoints fulfilled the eligibility criteria for this SR, but the multifactorial PREDIMED found a significant reduction in CVD in high-risk participants allocated to a Mediterranean diet supplemented with 30 g/day of nuts for 4.8 years, compared to the control group (120). The Mediterranean diet with nuts did not reduce the risk of T2D in a subgroup analysis of the trial (121). However, the design of the PREDIMED study precludes a clear interpretation of the separate effects of nuts from the Mediterranean diet (122). Still, this population was included in one of the cohort studies included in our SR, which found a strong, independent association between nut consumption at baseline and CVD mortality, although based on few events (84).

### Strengths and limitations of the systematic review

This systematic review was conducted with rigorous and transparent procedures, following current recommended principles and guidelines for SRs of nutritional research (31, 123). Strengths included a pre-registered protocol with a clear research question informed by a scoping review; pre-defined inclusion criteria methods; a comprehensive, peer-reviewed literature search strategy as well as hand-searches of reference lists; duplicate study selection, data extraction and RoB appraisal by two authors independently, including evaluation of possible publication bias. The certainty of the evidence was graded to facilitate translation of the findings into dietary guidelines. Dose-response relationships were explored to further inform the strength of evidence. Even though several SRs and meta-analyses on the subject have been published, we retrieved several recent papers with large sample sizes that has not previously been assessed. We were also able to include more studies in the non-linear dose-response analyses than previously, due to the use of the more efficient one-stage dose-response approach as opposed to the traditionally applied two-stage methods (55). Being based on the target experiment framework and focussing on internal validity, our risk-of-bias assessment of the cohort studies is more appropriate for interpreting causal associations, and for comparing evidence from RCTs and observational studies, than other often used summary score-based quality appraisal tools that are now advised against (33, 39). However, any assessment of the RoB involves subjective interpretations and is limited by the quality of the reporting.

We did not search trial registries or other sources of grey literature as the publications would not have been feasible for RoB assessment. Therefore, a more thorough investigation of publication bias was not possible. Nevertheless, our undertaken approach did not appear to be influenced by publication bias. Another weakness is the substantial to considerable heterogeneity in the results of some outcomes, which we could not immediately account for. Some heterogeneity would be expected, and the differences between the studies were mostly in the magnitude of the associations, not the direction. The overall findings were not sensitive to any one particular study and were broadly consistent across subgroups. Yet, our subgroup analyses and meta-regression analyses based on participant characteristics were compromised by the reliance upon study-level data, and are therefore fallible due to ecological bias (124).

Some previous SRs on CVD and T2D have included meta-analyses of individual types of nuts, which we did not, except for peanuts and CVD. However, these were based on very few studies, for example, three studies on peanuts and two on peanut butter and T2D (18), and one study on walnuts or peanut butter on CVD (17). The availability of evidence on specific nuts remains too limited for an informative meta-analysis, so we therefore reviewed them only narratively. This lack of specification of the exposure is a common weakness of the current literature. The exposure categories were also often poorly defined in the cohort studies and required some imputation and assumptions which makes the interpretation of the estimated dose-response relationships open to question. The intake assessments themselves were also likely to be affected by reporting errors but we could not appraise and compare the validity of the dietary assessment instruments used in the cohort studies. Although most stated that the methods were validated, they often referred to validation studies done in other cohorts or on nutrients/energy, as the cohorts were not originally designed to examine nut consumption per se. Moreover, few cohort studies with long follow-up assessed changes in intake over time.

As already mentioned, no RCTs reporting clinical endpoints were included, highlighting a gap in the literature as also pointed out by a 2015 Cochrane systematic review by Martin et al. (125). With only observational data available, there is inherently a concern for confounding by other lifestyle/dietary behaviours, perhaps most importantly other dietary factors. In most cohort studies, the effect estimates were adjusted for other foods, but only a few took the overall quality of the diet into account. Higher nut consumption is associated with improved total dietary quality (114, 126, 127), but that may in itself be partly a consequence of the nuts (128, 129). The risk of confounding biases was rated as moderate in almost all cohort studies. While confounding is still inevitable, the impact on the direction of the results is difficult to predict. Residual confounding can likely not explain why nut consumption was associated with lower risk of CVD and CHD, but not stroke or T2D. We are at least not aware of mechanisms that make CHD more susceptible to non-residual confounding than other outcomes regarding nut consumption.

Although the effect on blood lipids may be an important mediator of the associations, several cohort studies adjusted for hypercholesterolemia in the multivariable models, which may be a case of over-adjustment. We attempted to compare studies with and without this adjustment, finding no significant differences, but without individual participant data, such subgroup analyses have limitations. There is also evidence that frequent nut consumption is associated with lower weight gain and risk of overweight/obesity, partly due to effects on satiety and energy absorption (130, 131). Adjustments for BMI, as almost all cohort studies did, could be another example of over-adjustment possibly underestimating associations between nut consumption and cardiometabolic diseases (18).

The beneficial effects on total and LDL-C shown in RCTs might be interpreted as mechanistic support for the inverse association between nut consumption and CVD and CHD in the cohort studies, but these two lines of evidence have important differences and often pose different questions. Almost all RCTs tested the effects of one specific type of nuts, while almost all cohort studies assessed the intake of unspecified types of nuts with no data on processing or preparation. Second, the doses used in the RCTs were usually much higher than what is typically consumed and what may be realistically achievable for most consumers. The choice of comparators also differed between the RCTs, which may affect the result.

While the lipid-lowering effects of nuts in our meta-analyses were in the expected direction, this degree of reduction is too small to entirely account for the associations seen with CHD and total CVD. Even though the effect seemed clinically small it should be taken into consideration that CVD is mostly caused by accumulated risk factor exposures, and that large proportions of the decline in CHD and CHD mortality in previous decades have been attributed to even relatively modest population-level reductions in total-C (132–134). It is possible that the effects on blood lipids are greater when nuts primarily replace sources of saturated fats in the diet (135), a mediator that the included RCTs did not assess. Additionally, nuts and seeds may have effects on other important atherogenic lipid measures that we did not assess, such as apolipoprotein B (136).

By including only RCTs with a minimum 12 weeks of follow-up, we did not include a large number of shorter-term trials on risk factors. However, previous meta-analysis including short-term trials have found similar directions of effects (136–138). Further, we excluded trials of hypocaloric diets as the main intent of this SR was to inform dietary guidelines focusing on primary/primordial prevention, not clinical treatment guidelines.

In general, assessing only one single food group may be overly reductionist, hence a consideration of dietary patterns and replacement effects is recommended (123). A novel feature with our review in this regard is the exploration of substitution analyses. This was compromised by the sparse data, and we must note that we only included such analyses that were reported in the studies found through our literature search strategy, which was not developed specifically for studies on substitution analyses. Such substitution analyses are in any case hypothetical and not necessarily practically relevant (139).

### Comparison with previous findings

A complete account of previous SRs is outside the scope of this paper, but they are broadly in line with our findings, both directionally and quantitatively (17, 18, 25, 140–145). Several very large new studies have been published, motivating an updated synthesis. For instance, our results for CVD mortality alone covered about three times as many cases as the most recent previous meta-analysis (17). Interestingly, the summary results changed very little on inclusion of these recent studies. Bechthold et al. found no significant association with CHD nor stroke in a 2019 SR of cohort studies, but excluded all studies on CHD and stroke mortality (19).

As to the effects of nuts on blood lipids, a large number of reviews and meta-analyses have been published, usually focusing on one type of nuts. One network meta-analysis by Schwingshackl et al. ranked nuts highest of food groups for lowering total and LDL-C and other risk factors, such as blood pressure and glucose (24). Liu et al. further ranked pistachios and walnuts highest for total-C, while pistachios and almonds were most effective in lowering LDL-C (146). However, comparing our results with previous meta-analyses is difficult as most have included relatively short-term interventions and/or studies involving weight-loss. In one of the largest meta-analyses on nuts and blood lipids, Del Gobbo et al. found that nuts were associated with 4.8 mg/dL lower LDL-C and 4.7 mg/dL lower total-C (about 0.12 mmol/L) per serving/day in 61 trials (136). Even though the median duration of the trials in that meta-analysis was only 4 weeks, the results were similar to ours (136).

Perhaps unexpected, but also in line with our results, no statistically significant effects of nuts on blood pressure have been found in other recent meta-analyses. Some have found that only pistachios had an effect (16, 147, 148), but our meta-analyses included only two trials of pistachios on blood pressure.

Previous SRs have also found inconsistent and inconclusive association between nuts and T2D risk. While one meta-analysis from 2014 did find an inverse association (per 4 servings/week) (142), other, more recent SRs have failed to find an association between nut consumption and T2D (18, 25). While we did not perform meta-analyses of glycaemic markers, previous meta-analyses have generally found no effects. Beneficial effects on glucose have been found in patients with established T2D, which we excluded (21, 149). Tindall et al. found in a large meta-analysis including subjects both with and without diabetes a significant reduction in HOMA-IR (a mean difference of −0.23 in 19 studies), but no significant overall effects on glucose or HbA1c (23). Concerning specific nuts, lack of effects on either fasting glucose, HbA1c or HOMA-IR have been found with walnuts and almonds in previous meta-analyses (150, 151).

### Interpretation of findings

The mechanistic rationale for an effect of nuts and seeds on cardiometabolic risk factors, atherosclerosis and risk of CVD has been characterized in several reviews (152–155). Beneficial effects can be deduced from their contents and combinations of nutrients and bioactive substances. The cholesterol-lowering effects of nuts are in part attributed to the fatty acid composition, considering their low saturated fatty acid (SFA) content relative to PUFA (e.g. walnuts and seeds) and MUFA (e.g. hazelnuts, peanuts and almonds), although the fibre, micronutrients and polyphenols and other components may also be involved (22). Nuts and seeds are also among the few natural food sources of phytosterols, which reduce cholesterol absorption and increase excretion. It is thus interesting to note that we found larger effects on LDL-C in the RCTs of pistachio nuts and almonds, which have especially high concentrations of phytosterols among nuts (156). In a meta-regression, Del Gobbo et al. found that the total phytosterol dose from nuts was indeed inversely correlated with the reduction in LDL-C, but this was not independent of total nut dose (157). A range of other effects, for example, on vascular function, oxidative stress and inflammation, likely also play a part in the associations observed with CHD and overall CVD (78, 158–160).

Being a source of different minerals and certain amino acids, nuts could be expected to lower blood pressure. We found unclear, limited evidence for associations between nut consumption and stroke in cohort studies, and overall, no effects on blood pressure in RCTs. We considered this suggestive, but limited evidence for no direct causal relationship between nut consumption and stroke, although several questions can be raised. The number of stroke events in the cohort studies were lower than that of CHD, and the studies may therefore have been underpowered. One could also speculate that the seemingly small benefits on stroke may have been affected by added sodium in some nut products, which is difficult to account for with self-reported dietary data. Due to the usually low intakes observed, it seems unlikely that nuts were an important source of salt in these populations. This is also contradicted by some other prospective studies showing an inverse association between nuts and hypertension (161, 162). However, in some populations, high nut consumption could have been associated with a snacking eating pattern. Perhaps the type of nuts also is a more important factor with stroke, as there were significant, inverse associations with peanuts in four studies and with walnuts in one (42, 43, 87).

Indeed, the PREDIMED trial, which included a high-risk population, did find a large reduction in the risk of stroke in the group on a Mediterranean diet with nuts (50% were walnuts) versus the control group (HR 0.54, 95% CI .35, 0.82) (120). This underscores the need for evaluating nuts within the context of the dietary pattern. To this end, we assessed food substitution analyses in the cohort studies to consider the potential impact on risk associated with iso-caloric comparisons of other foods with nuts (35, 73, 77, 98, 102). These studies generally (statistically) interchanged protein foods, and suggested inverse associations with both CVD, CHD, stroke and T2D when nuts replaced meat. Previous SRs on nuts have not addressed this question. Hidayat et al. recently published a meta-analysis regarding replacement of red meat with other protein sources, and concluded that replacing red meat with nuts was associated with lower risk of CHD and all-cause mortality (163).

### Public health relevance and implications

Cardiovascular disease is a leading cause of death across the world. Ischaemic heart disease in particular is a major cause of premature deaths and disease burden despite large decreases in incidence and mortality in recent decades. The cases are largely preventable, and diet is the main attributable risk factor globally (164).

Based on aspects such as strength of associations, coherence, consistency, and plausibility, nuts/seeds was one of the food groups deemed to have at least probable or convincing evidence for protective associations with CVD and CHD, but not T2D, in a recent assessment of quality of the evidence for foods and nutrients and cardiometabolic disease (165). Our comprehensive, up to date SR corroborates this view, especially regarding inverse dose-response associations between nuts and seeds consumption and overall CVD and CHD, further reinforced by a lowering effect on LDL-C that support the hypothesis that part of the associations between nuts and CVD endpoints are due to effects on blood lipids.

The effects on LDL-C were not large, and more than the usually recommended ‘one handful’ [close to 30 g (50)] of nuts may be needed for a clinically significant reduction. As with all dietary exposures, small effects may still be relevant for population-level prevention, especially in a life-course perspective (166). With respect to public health it is also worth noting that by an increased nut consumption up to 30 g/day, the resulting decrease in CVD risk has recently been estimated to considerably outweigh the potential risk for liver cancer related to increased exposure to aflatoxin B (167).

## Conclusion

In a ‘nutshell’, higher consumption of nuts and seeds have a probable causal, inverse association with CVD and CHD, while the evidence is limited for associations with stroke and T2D. Our review also highlights the need for more high-quality, standardized research and adherence to reporting guidelines in order to better characterize the strength of the evidence. To advance the field, there should be more research done on specific types of nuts and seeds, consumption patterns and elucidation of mechanisms, preferably in large-scale clinical trials and individual participant meta-analyses.

## Supplementary Material

Nuts and seeds consumption and risk of cardiovascular disease, type 2 diabetes and their risk factors: a systematic review and meta-analysis

## Registration

PROSPERO registration number: CRD42021270554.
