# Supplementary material for: Nuts and seeds consumption and risk of cardiovascular disease, type 2 diabetes and their risk factors: a systematic review and meta-analysis
Source: Food Nutr Res. 2023 Feb 14;67:10.29219/fnr.v67.8961. doi: 10.29219/fnr.v67.8961 (PMC9930735; doi:10.29219/fnr.v67.8961)
Supplement: Nuts and seeds consumption and risk of cardiovascular disease, type 2 diabetes and their risk factors: a systematic review and meta-analysis [file FNR-67-8961-s001.docx]

Supplementary material

## Search strategy

All searches were performed on 20.09.2021 by academic librarian Hilde Strømme, University of Oslo Library of Medicine and Science. Search strategies were peer reviewed by Sabina Gillsund and Narcisa Hannerz at the Karolinska Institutet University Library.

**Ovid MEDLINE**

| 1 | Nuts/ | 3597 |
| --- | --- | --- |
| 2 | (nut or nuts or almond* or pistachio* or walnut* or cashew* or pecan* or macadamia* or hazelnut* or peanut* or chestnut* or coconut* or linseed* or flaxseed* or seed or seeds or (pine adj (nut* or kernel*)) or ((sunflower or pumpkin) adj (seed* or kernel*))).tw,kf. | 150388 |
| 3 | 1 or 2 | 150870 |
| 4 | exp Lipoproteins/ or exp Cholesterol/ or exp Triglycerides/ or exp Blood Pressure/ or exp Hypertension/ or Blood Glucose/ or Glycated Hemoglobin A/ or Insulin Resistance/ | 967493 |
| 5 | (lipoprotein* or apolipoprotein* or cholesterol* or triglyceride* or triacylglyc* or ((blood or diastolic or systolic or pulse or arterial or aortic or venous or vascular or portal) adj (pressure or tension)) or hypertension or (blood adj (glucose or sugar)) or (glyc* adj (hemoglobin or haemoglobin)) or HbA1c or (insulin adj (resistan* or sensitiv* or response))).tw,kf. | 1201514 |
| 6 | 4 or 5 | 1495703 |
| 7 | ((randomized controlled trial or controlled clinical trial).pt. or (randomized or placebo or randomly).ab. or trial.ti. or clinical trials as topic.sh.) not (exp animals/ not humans.sh.) | 1283553 |
| 8 | 3 and 6 and 7 | 1095 |
| 9 | Cardiovascular Diseases/ or Atherosclerosis/ or exp Myocardial Infarction/ or exp Stroke/ or exp Coronary Disease/ or exp Coronary Artery Bypass/ | 720652 |
| 10 | ((ACA or anterior cerebral artery or anterior cerebral circulation or anterior choroidal artery or brain or brain stem or brainstem or brain venous or cerebral or heart or heubner* artery or MCA or middle cerebral artery or myocardial or PCA or posterior cerebral artery or posterior choroidal artery or subcortical) adj2 infarct*).tw,kf. | 240313 |
| 11 | ((anterior cerebral artery or basilar or benedict or claude or coronary-subclavian steal or dorsolateral medullary or foville or lateral bulbar or lateral medullary or middle cerebral artery or millard-gublar or posterior cerebral artery or posterior inferior cerebellar artery or wallenberg* or weber) adj2 syndrome*).tw,kf. | 3544 |
| 12 | ((brain vascular or cerebrovascular) adj2 accident*).tw,kf. | 7713 |
| 13 | ((coronary artery or aortocoronary) adj2 bypass*).tw,kf. | 45884 |
| 14 | (coronary adj3 (aneurysm* or arterioscleros#s or artery anastomos#s or disease* or occlusion* or restenos#s or stenos#s or syndrome* or thrombos#s or vasospasm*)).tw,kf. | 222596 |
| 15 | (apoplex* or atherogenesis or atheroscleros#s or cardiogenic shock or heart attack* or middle cerebral artery thrombosis or stroke*).tw,kf. | 417131 |
| 16 | ((cardio* or cerebral* or cerebro* or coronary or CVD or heart* or myocardial) adj3 (death? or mortalit*)).tw,kf. | 94287 |
| 17 | exp "Diabetes Mellitus, Type 2"/ or exp Insulin Resistance/ or C-Peptide/ or Glucose Intolerance/ or Glycated Hemoglobin A/ or Blood Glucose/ or Hyperglycemia/ or Blood Pressure/ or Hypertension/ or Lipids/ or exp Triglycerides/ or exp Apolipoproteins/ or Cholesterol/ or Cholesterol, HDL/ or Cholesterol, LDL/ or Cholesterol, VLDL/ | 1052278 |
| 18 | (diabet* adj3 ("2" or "type II" or Adult-Onset or Non Insulin or NonInsulin)).tw,kf. | 181137 |
| 19 | (DM2 or NIDDM or IIDM or MODY or T2DM).tw,kf. | 35576 |
| 20 | (blood adj2 (glucose or sugar*)).tw,kf. | 94019 |
| 21 | (lipid* adj2 (blood or level or profile*)).tw,kf. | 56391 |
| 22 | (blood pressure or cardiometabolic syndrome* or C-peptide or cholesterol or HDL or LDL or VLDL or connecting peptide or diastolic pressure or dysmetabolic syndrome* or glucose intolerance* or HOMA-IR or hyperglycemia* or hypertension or insulin resistance or insulin sensitivity or metabolic syndrome* or metabolic cardiovascular syndrome* or pulse pressure or reaven syndrome X or systolic pressure).tw,kf. | 1042783 |
| 23 | ((glycated or glycosylated) adj2 (haemoglobin* or hemoglobin*)).tw,kf. | 24507 |
| 24 | (glycohemoglobin A or Hb A1 or HbA1 or Hb A1a-1 or Hb A1a-2 or Hb A1a+b or Hb A1b or Hb A1c or HbA1c or "hemoglobin A(1)" or hemoglobin A1C).tw,kf. | 47628 |
| 25 | (Apo-B or ApoA or ApoA-II or Apo A-V or Apo A1 or Apo A2 or Apo A5 or APOA5 or Apo AI or ApoB or ApoB48 or ApoC or Apo C or ApoC2 or Apo D or ApoD or ApoE or Apo E or APOE-epsilon* or ApoE2 or Apo E2 or Apo E3 or ApoE3 or Apo E4 or ApoE4 or ApoL or ApoL1 or apolipoprotein* or apoprotein* or enzactin or glycerol trioleate or proapolipoprotein* or triacetin or triacetyl-glycerol* or triacetylglycerol* or triacylglycerol* or trielaidin or triglyceride* or trioleate-glycerin or triolein or trioleoylglycerol or trioleyl glycerol*).tw,kf. | 194944 |
| 26 | or/9-25 | 2446638 |
| 27 | 3 and 26 | 10603 |
| 28 | 8 or 27 | 10612 |
| 29 | 28 not (exp "Animals"/ not (exp "Animals"/ and "Humans"/)) | 7151 |
| 30 | 29 not (editorial or interview or letter or comment or congress or legal case or meeting abstract).pt. | 7041 |

**Embase Classic+Embase (Ovid)**

| 1 | exp Nuts/ | 21334 |
| --- | --- | --- |
| 2 | (nut or nuts or almond* or pistachio* or walnut* or cashew* or pecan* or macadamia* or hazelnut* or peanut* or chestnut* or coconut* or linseed* or flaxseed* or seed or seeds or (pine adj (nut* or kernel*)) or ((sunflower or pumpkin) adj (seed* or kernel*))).tw,kw. | 178027 |
| 3 | 1 or 2 | 182982 |
| 4 | exp lipoprotein/ or exp cholesterol ester/ or exp triacylglycerol/ or exp blood pressure/ or exp elevated blood pressure/ or glucose blood level/ or hemoglobin A1c/ or exp insulin response/ | 2040612 |
| 5 | (lipoprotein* or apolipoprotein* or cholesterol* or triglyceride* or triacylglyc* or ((blood or diastolic or systolic or pulse or arterial or aortic or venous or vascular or portal) adj (pressure or tension)) or hypertension or (blood adj (glucose or sugar)) or (glyc* adj (hemoglobin or haemoglobin)) or HbA1c or (insulin adj (resistan* or sensitiv* or response))).tw,kw. | 1780676 |
| 6 | 4 or 5 | 2503096 |
| 7 | (Randomized controlled trial/ or Controlled clinical trial/ or random$.ti,ab. or randomization/ or intermethod comparison/ or placebo.ti,ab. or (compare or compared or comparison).ti. or ((evaluated or evaluate or evaluating or assessed or assess) and (compare or compared or comparing or comparison)).ab. or (open adj label).ti,ab. or ((double or single or doubly or singly) adj (blind or blinded or blindly)).ti,ab. or double blind procedure/ or parallel group$1.ti,ab. or (crossover or cross over).ti,ab. or ((assign$ or match or matched or allocation) adj5 (alternate or group$1 or intervention$1 or patient$1 or subject$1 or participant$1)).ti,ab. or (assigned or allocated).ti,ab. or (controlled adj7 (study or design or trial)).ti,ab. or (volunteer or volunteers).ti,ab. or human experiment/ or trial.ti.) not (((random$ adj sampl$ adj7 (cross section$ or questionnaire$1 or survey$ or database$1)).ti,ab. not (comparative study/ or controlled study/ or randomi?ed controlled.ti,ab. or randomly assigned.ti,ab.)) or (Cross-sectional study/ not (randomized controlled trial/ or controlled clinical study/ or controlled study/ or randomi?ed controlled.ti,ab. or control group$1.ti,ab.)) or (((case adj control$) and random$) not randomi?ed controlled).ti,ab. or (Systematic review not (trial or study)).ti. or (nonrandom$ not random$).ti,ab. or Random field$.ti,ab. or (random cluster adj3 sampl$).ti,ab. or ((review.ab. and review.pt.) not trial.ti.) or (we searched.ab. and (review.ti. or review.pt.)) or update review.ab. or (databases adj4 searched).ab. or ((rat or rats or mouse or mice or swine or porcine or murine or sheep or lambs or pigs or piglets or rabbit or rabbits or cat or cats or dog or dogs or cattle or bovine or monkey or monkeys or trout or marmoset$1).ti. and animal experiment/) or (Animal experiment/ not (human experiment/ or human/))) | 4974907 |
| 8 | 3 and 6 and 7 | 2718 |
| 9 | Cardiovascular Diseases/ or Atherosclerosis/ or exp Myocardial Infarction/ or exp Stroke/ or exp Coronary Disease/ or exp Coronary Artery Bypass/ or exp "Diabetes Mellitus, Type 2"/ or exp Insulin Resistance/ or C-Peptide/ or Glucose Intolerance/ or Glycated Hemoglobin A/ or Blood Glucose/ or Hyperglycemia/ or Blood Pressure/ or Hypertension/ or Lipids/ or exp Triglycerides/ or exp Apolipoproteins/ or Cholesterol/ or Cholesterol, HDL/ or Cholesterol, LDL/ or Cholesterol, VLDL/ | 2763872 |
| 10 | ((ACA or anterior cerebral artery or anterior cerebral circulation or anterior choroidal artery or brain or brain stem or brainstem or brain venous or cerebral or heart or heubner* artery or MCA or middle cerebral artery or myocardial or PCA or posterior cerebral artery or posterior choroidal artery or subcortical) adj2 infarct*).tw,kw. | 353096 |
| 11 | ((anterior cerebral artery or basilar or benedict or claude or coronary-subclavian steal or dorsolateral medullary or foville or lateral bulbar or lateral medullary or middle cerebral artery or millard-gublar or posterior cerebral artery or posterior inferior cerebellar artery or wallenberg* or weber) adj2 syndrome*).tw,kw. | 4870 |
| 12 | ((brain vascular or cerebrovascular) adj2 accident*).tw,kw. | 12200 |
| 13 | ((coronary artery or aortocoronary) adj2 bypass*).tw,kw. | 60105 |
| 14 | (coronary adj3 (aneurysm* or arterioscleros#s or artery anastomos#s or disease* or occlusion* or restenos#s or stenos#s or syndrome* or thrombos#s or vasospasm*)).tw,kw. | 327743 |
| 15 | (apoplex* or atherogenesis or atheroscleros#s or cardiogenic shock or heart attack* or middle cerebral artery thrombosis or stroke*).tw,kw. | 660409 |
| 16 | ((cardio* or cerebral* or cerebro* or coronary or CVD or heart* or myocardial) adj3 (death? or mortalit*)).tw,kw. | 154218 |
| 17 | non insulin dependent diabetes mellitus/ or insulin resistance/ or c peptide/ or glucose intolerance/ or hemoglobin a1c/ or glucose blood level/ or hyperglycemia/ or blood pressure/ or hypertension/ or exp lipid blood level/ or exp apolipoprotein/ or high density lipoprotein cholesterol/ or low density lipoprotein cholesterol/ or very low density lipoprotein cholesterol/ | 1726925 |
| 18 | (diabet* adj3 ("2" or "type II" or Adult-Onset or Non Insulin or NonInsulin)).tw,kw. | 275133 |
| 19 | (DM2 or NIDDM or IIDM or MODY or T2DM).tw,kw. | 58490 |
| 20 | (blood adj2 (glucose or sugar*)).tw,kw. | 148972 |
| 21 | (lipid* adj2 (blood or level or profile*)).tw,kw. | 80705 |
| 22 | (blood pressure or cardiometabolic syndrome* or C-peptide or cholesterol or HDL or LDL or VLDL or connecting peptide or diastolic pressure or dysmetabolic syndrome* or glucose intolerance* or HOMA-IR or hyperglycemia* or hypertension or insulin resistance or insulin sensitivity or metabolic syndrome* or metabolic cardiovascular syndrome* or pulse pressure or reaven syndrome X or systolic pressure).tw,kw. | 1563277 |
| 23 | ((glycated or glycosylated) adj2 (haemoglobin* or hemoglobin*)).tw,kw. | 32282 |
| 24 | (glycohemoglobin A or Hb A1 or HbA1 or Hb A1a-1 or Hb A1a-2 or Hb A1a+b or Hb A1b or Hb A1c or HbA1c or "hemoglobin A(1)" or hemoglobin A1C).tw,kw. | 95462 |
| 25 | (Apo-B or ApoA or ApoA-II or Apo A-V or Apo A1 or Apo A2 or Apo A5 or APOA5 or Apo AI or ApoB or ApoB48 or ApoC or Apo C or ApoC2 or Apo D or ApoD or ApoE or Apo E or APOE-epsilon* or ApoE2 or Apo E2 or Apo E3 or ApoE3 or Apo E4 or ApoE4 or ApoL or ApoL1 or apolipoprotein* or apoprotein* or enzactin or glycerol trioleate or proapolipoprotein* or triacetin or triacetyl-glycerol* or triacetylglycerol* or triacylglycerol* or trielaidin or triglyceride* or trioleate-glycerin or triolein or trioleoylglycerol or trioleyl glycerol*).tw,kw. | 274542 |
| 26 | or/9-20 | 3276679 |
| 27 | 3 and 26 | 14695 |
| 28 | 8 or 27 | 15088 |
| 29 | 28 not ("animal"/ not "human"/) | 14241 |
| 30 | 29 not (Conference abstract or Conference paper or Conference review or Editorial or Letter or Note or Short survey).pt. | 11387 |

**Cochrane Central Register of Controlled Trials**

| #1 | [mh ^Nuts] | 267 |
| --- | --- | --- |
| #2 | (nut OR nuts OR almond* OR pistachio* OR walnut* OR cashew* OR pecan* OR macadamia* OR hazelnut* OR peanut* OR chestnut* OR coconut* OR linseed* OR flaxseed* OR seed OR seeds OR (pine NEXT (nut* OR kernel*)) OR ((sunflower OR pumpkin) NEXT (seed* OR kernel*))):ti,ab,kw | 7289 |
| #3 | #1 OR #2 | 7289 |
| #4 | [mh Lipoproteins] OR [mh Cholesterol] OR [mh Triglycerides] OR [mh "Blood Pressure"] OR [mh Hypertension] OR [mh ^"Blood Glucose"] OR [mh ^"Glycated Hemoglobin A"] OR [mh ^"Insulin Resistance"] | 67665 |
| #5 | (lipoprotein* OR apolipoprotein* OR cholesterol* OR triglyceride* OR triacylglyc* OR ((blood OR diastolic OR systolic OR pulse OR arterial OR aortic OR venous OR vascular OR portal ) NEXT (pressure OR tension )) OR hypertension OR (blood NEXT (glucose OR sugar )) OR (glyc* NEXT (hemoglobin OR haemoglobin )) OR HbA1c OR (insulin NEXT (resistan* OR sensitiv* OR response ))):ti,ab,kw | 210125 |
| #6 | ([mh ^"Cardiovascular Diseases"] OR [mh ^Atherosclerosis] OR [mh "Myocardial Infarction"] OR [mh Stroke] OR [mh "Coronary Disease"] OR [mh "Coronary Artery Bypass"]) | 45167 |
| #7 | ((ACA OR "anterior cerebral artery" OR "anterior cerebral circulation" OR "anterior choroidal artery" OR brain OR "brain stem" OR brainstem OR "brain venous" OR cerebral OR heart OR (heubner* NEXT artery) OR MCA OR "middle cerebral artery" OR myocardial OR PCA OR "posterior cerebral artery" OR "posterior choroidal artery" OR subcortical) NEAR/2 infarct*):ti,ab,kw | 40581 |
| #8 | (("anterior cerebral artery" OR basilar OR benedict OR claude OR "coronary-subclavian steal" OR "dorsolateral medullary" OR foville OR "lateral bulbar" OR "lateral medullary" OR "middle cerebral artery" OR millard-gublar OR "posterior cerebral artery" OR "posterior inferior cerebellar artery" OR wallenberg* OR weber) NEAR/2 syndrome*):ti,ab,kw | 48 |
| #9 | (("brain vascular" OR cerebrovascular) NEAR/2 accident*):ti,ab,kw | 14095 |
| #10 | (("coronary artery" OR aortocoronary) NEAR/2 bypass*):ti,ab,kw | 12403 |
| #11 | (coronary NEAR/3 (aneurysm* OR arterioscleros?s OR "artery anastomos")):ti,ab,kw | 190 |
| #12 | (apoplex* OR atherogenesis OR atheroscleros?s OR "cardiogenic shock" OR ("heart" NEXT attack*) OR "middle cerebral artery thrombosis" OR stroke*):ti,ab,kw | 70564 |
| #13 | ((cardio* OR cerebral* OR cerebro* OR coronary OR CVD OR heart* OR myocardial) NEAR/3 (death? OR mortalit*)):ti,ab,kw | 23741 |
| #14 | [mh "Diabetes Mellitus, Type 2"] OR [mh "Insulin Resistance"] OR [mh ^"C-Peptide"] OR [mh ^"Glucose Intolerance"] OR [mh ^"Glycated Hemoglobin A"] OR [mh ^"Blood Glucose"] OR [mh ^Hyperglycemia] OR [mh ^"Blood Pressure"] OR [mh ^Hypertension] OR [mh ^Lipids] OR [mh Triglycerides] OR [mh Apolipoproteins] OR [mh ^Cholesterol] OR [mh ^"Cholesterol, HDL"] OR [mh ^"Cholesterol, LDL"] OR [mh ^"Cholesterol, VLDL"] | 78691 |
| #15 | (diabet* NEAR/3 (2 OR "type II" OR "Adult-Onset" OR "Non Insulin" OR NonInsulin)):ti,ab,kw | 47031 |
| #16 | (DM2 OR NIDDM OR IIDM OR MODY OR T2DM):ti,ab,kw | 8314 |
| #17 | (blood NEAR/2 (glucose OR sugar*)):ti,ab,kw | 42834 |
| #18 | (lipid* NEAR/2 (blood OR level OR profile*)):ti,ab,kw | 20292 |
| #19 | ("blood pressure" OR (cardiometabolic NEXT syndrome*) OR "C-peptide" OR cholesterol OR HDL OR LDL OR VLDL OR "connecting peptide" OR "diastolic pressure" OR (dysmetabolic NEXT syndrome*) OR (glucose NEXT intolerance*) OR "HOMA-IR" OR hyperglycemia* OR hypertension OR "insulin resistance" OR "insulin sensitivity" OR (metabolic NEXT syndrome*) OR ("metabolic cardiovascular" NEXT syndrome*) OR "pulse pressure" OR "reaven syndrome X" OR "systolic pressure"):ti,ab,kw | 181665 |
| #20 | ((glycated or glycosylated) NEAR/2 (haemoglobin* or hemoglobin*)):ti,ab,kw | 11461 |
| #21 | ("glycohemoglobin A" OR "Hb A1" OR HbA1 OR "Hb A1a-1" OR "Hb A1a-2" OR "Hb A1a+b" OR "Hb A1b" OR "Hb A1c" OR HbA1c OR "hemoglobin A(1)" OR "hemoglobin A1C"):ti,ab,kw | 23151 |
| #22 | ("Apo-B" OR ApoA OR "ApoA-II" OR "Apo A-V" OR "Apo A1" OR "Apo A2" OR "Apo A5" OR APOA5 OR "Apo AI" OR ApoB OR ApoB48 OR ApoC OR "Apo C" OR ApoC2 OR "Apo D" OR ApoD OR ApoE OR "Apo E" OR (APOE NEXT epsilon*) OR ApoE2 OR "Apo E2" OR "Apo E3" OR ApoE3 OR "Apo E4" OR ApoE4 OR ApoL OR ApoL1 OR apolipoprotein* OR apoprotein* OR enzactin OR "glycerol trioleate" OR proapolipoprotein* OR triacetin OR (triacetyl NEXT glycerol*) OR triacetylglycerol* OR triacylglycerol* OR trielaidin OR triglyceride* OR (trioleate NEXT glycerin) OR triolein OR trioleoylglycerol OR (trioleyl NEXT glycerol*)):ti,ab,kw | 30014 |
| #23 | #4 OR #5 OR #6 OR #7 OR #8 OR #9 OR #10 OR #11 OR #12 OR #13 OR #14 OR #15 OR #16 OR #17 OR #18 OR #19 OR #20 OR #21 OR #22 | 328905 |
| #24 | #3 AND #23 | 2458 |
| #25 | #24 in Trials | 2449 |

**Scopus (Elsevier)**

( ( TITLE-ABS-KEY ( nut OR nuts OR almond* OR pistachio* OR walnut* OR cashew* OR pecan* OR macadamia* OR hazelnut* OR peanut* OR chestnut* OR coconut* OR linseed* OR flaxseed* OR seed OR seeds OR ( pine W/1 ( nut* OR kernel* ) ) OR ( ( sunflower OR pumpkin ) W/1 ( seed* OR kernel* ) ) ) AND TITLE-ABS-KEY ( ( ( "ACA" OR "anterior cerebral artery" OR "anterior cerebral circulation" OR "anterior choroidal artery" OR "brain" OR "brain stem" OR "brainstem" OR "brain venous" OR "cerebral" OR "heart" OR "heubner* artery" OR "MCA" OR "middle cerebral artery" OR "myocardial" OR "PCA" OR "posterior cerebral artery" OR "posterior choroidal artery" OR "subcortical" ) W/1 "infarct*" ) OR ( ( "anterior cerebral artery" OR "basilar" OR "benedict" OR "claude" OR "coronary-subclavian steal" OR "dorsolateral medullary" OR "foville" OR "lateral bulbar" OR "lateral medullary" OR "middle cerebral artery" OR "millard-gublar" OR "posterior cerebral artery" OR "posterior inferior cerebellar artery" OR "wallenberg*" OR "weber" ) W/1 "syndrome*" ) OR ( ( "coronary artery" OR "aortocoronary" ) W/1 "bypass*" ) OR ( "coronary" W/2 ( "aneurysm*" OR "arterioscleros*" OR "artery anastomos" ) ) OR ( "apoplex*" OR "atherogenesis" OR "atheroscleros*" OR "cardiogenic shock" OR "heart attack*" OR "middle cerebral artery thrombosis" OR "stroke*" ) OR ( "diabet*" W/2 ( "2" OR "type II" OR "Adult-Onset" OR "Non Insulin" OR "NonInsulin" ) ) OR ( "DM2" OR "NIDDM" OR "IIDM" OR "MODY" OR "T2DM" ) OR ( "blood" W/1 ( "glucose" OR "sugar*" ) ) OR ( "lipid*" W/1 ( "blood" OR "level" OR "profile*" ) ) OR ( "blood pressure" OR "cardiometabolic syndrome*" OR "C-peptide" OR "cholesterol" OR "HDL" OR "LDL" OR "VLDL" OR "connecting peptide" OR "diastolic pressure" OR "dysmetabolic syndrome*" OR "glucose intolerance*" OR "HOMA-IR" OR "hyperglycemia*" OR "hypertension" OR "insulin resistance" OR "insulin sensitivity" OR "metabolic syndrome*" OR "metabolic cardiovascular syndrome*" OR "pulse pressure" OR "reaven syndrome X" OR "systolic pressure" ) OR ( ( "glycated" OR "glycosylated" ) W/1 ( "haemoglobin*" OR "hemoglobin*" ) ) OR ( "glycohemoglobin A" OR "Hb A1" OR "HbA1" OR "Hb A1a-1" OR "Hb A1a-2" OR "Hb A1a+b" OR "Hb A1b" OR "Hb A1c" OR "HbA1c" OR "hemoglobin A(1)" OR "hemoglobin A1C" ) OR ( "Apo-B" OR "ApoA" OR "ApoA-II" OR "Apo A-V" OR "Apo A1" OR "Apo A2" OR "Apo A5" OR "APOA5" OR "Apo AI" OR "ApoB" OR "ApoB48" OR "ApoC" OR "Apo C" OR "ApoC2" OR "Apo D" OR "ApoD" OR "ApoE" OR "Apo E" OR "APOE epsilon*" OR "ApoE2" OR "Apo E2" OR "Apo E3" OR "ApoE3" OR "Apo E4" OR "ApoE4" OR "ApoL" OR "ApoL1" OR "apolipoprotein*" OR "apoprotein*" OR "enzactin" OR "glycerol trioleate" OR "proapolipoprotein*" OR "triacetin" OR "triacetyl glycerol*" OR "triacetylglycerol*" OR "triacylglycerol*" OR "trielaidin" OR "triglyceride*" OR "trioleate-glycerin" OR "triolein" OR "trioleoylglycerol" OR "trioleyl glycerol*" ) ) ) OR ( TITLE-ABS-KEY ( ( nut OR nuts OR almond* OR pistachio* OR walnut* OR cashew* OR pecan* OR macadamia* OR hazelnut* OR peanut* OR chestnut* OR coconut* OR linseed* OR flaxseed* OR seed OR seeds OR ( pine W/1 ( nut* OR kernel* ) ) OR ( ( sunflower OR pumpkin ) W/1 ( seed* OR kernel* ) ) ) AND ( lipoprotein* OR apolipoprotein* OR cholesterol* OR triglyceride* OR triacylglyc* OR ( ( blood OR diastolic OR systolic OR pulse OR arterial OR aortic OR venous OR vascular OR portal ) W/1 ( pressure OR tension ) ) OR hypertension OR ( blood W/1 ( glucose OR sugar ) ) OR ( glyc* W/1 ( hemoglobin OR haemoglobin ) ) OR hba1c OR ( insulin W/1 ( resistan* OR sensitiv* OR response ) ) ) ) AND ( INDEXTERMS ( "clinical trials" OR "clinical trials as a topic" OR "randomized controlled trial" OR "Randomized Controlled Trials as Topic" OR "controlled clinical trial" OR "Controlled Clinical Trials" OR "random allocation" OR "Double-Blind Method" OR "Single-Blind Method" OR "Cross-Over Studies" OR "Placebos" OR "multicenter study" OR "double blind procedure" OR "single blind procedure" OR "crossover procedure" OR "clinical trial" OR "controlled study" OR "randomization" OR "placebo" ) ) OR ( TITLE-ABS-KEY ( ( "clinical trials" OR "clinical trials as a topic" OR "randomized controlled trial" OR "Randomized Controlled Trials as Topic" OR "controlled clinical trial" OR "Controlled Clinical Trials as Topic" OR "random allocation" OR "randomly allocated" OR "allocated randomly" OR "Double-Blind Method" OR "Single-Blind Method" OR "Cross-Over Studies" OR "Placebos" OR "cross-over trial" OR "single blind" OR "double blind" OR "factorial design" OR "factorial trial" ) ) ) OR ( TITLE-ABS ( clinical AND trial* OR trial* OR rct* OR random* OR blind* ) ) ) ) AND ( EXCLUDE ( DOCTYPE , "re" ) OR EXCLUDE ( DOCTYPE , "cp" ) OR EXCLUDE ( DOCTYPE , "ch" ) OR EXCLUDE ( DOCTYPE , "no" ) OR EXCLUDE ( DOCTYPE , "le" ) OR EXCLUDE ( DOCTYPE , "ed" ) OR EXCLUDE ( DOCTYPE , "sh" ) OR EXCLUDE ( DOCTYPE , "cr" ) OR EXCLUDE ( DOCTYPE , "bk" ) )

## Supplementary Tables

### Supplementary Table 1. Studies excluded after fulltext-screening

| Full reference | Reason for exclusion |
| --- | --- |
| Ahmadniay Motlagh, H., et al., Effect of flaxseed consumption on central obesity, serum lipids, and adiponectin level in overweight or obese women: A randomised controlled clinical trial. International Journal of Clinical Practice., 2021. 75(10):e14592. | Wrong intervention |
| Akbaraly, T.N., et al., Alternative healthy eating index and mortality over 18 y of follow-up: Results from the Whitehall II cohort. American Journal of Clinical Nutrition, 2011. 94: p. 247-253. | Wrong exposure |
| Alwosais, E.Z.M., et al., Chia seed (Salvia hispanica L.) supplementation to the diet of adults with type 2 diabetes improved systolic blood pressure: A randomized controlled trial. Nutrition and health, 2021. 27: p. 181-189. | Wrong population |
| Arab, L., et al., Association between walnut consumption and diabetes risk in NHANES. Diabetes/Metabolism Research and Reviews, 2018. 34. | Wrong study design |
| Babio, N., et al., Mediterranean diets and metabolic syndrome status in the PREDIMED randomized trial. CMAJ, 2014. 186(17): p. E649‐E657. | Wrong outcome  Wrong intervention |
| Badri, N.W., et al., Insulin Resistance Improves More in Women than In Men in Association with a Weight Loss Intervention. Journal of Obesity & Weight Loss Therapy, 2018. 8(1) | Wrong intervention |
| Baer, H.J., et al., Risk factors for mortality in the nurses' health study: A competing risks analysis. American Journal of Epidemiology, 2011. 173: p. 319-329. | Population included in more recent study |
| Bao, Y., et al., Association of nut consumption with total and cause-specific mortality. New England Journal of Medicine, 2013. 369: p. 2001-2011. | Population included in more recent study |
| Belin, R.J., et al., Diet quality and the risk of cardiovascular disease: The Women's Health Initiative (WHI). American Journal of Clinical Nutrition, 2011. 94: p. 49-57. | Wrong exposure |
| Casas, R., et al., Long-Term Immunomodulatory Effects of a Mediterranean Diet in Adults at High Risk of Cardiovascular Disease in the PREvención con DIeta MEDiterránea (PREDIMED) Randomized Controlled Trial. Journal of nutrition, 2016. 146(9): p. 1684‐1693. | Wrong intervention |
| Dilis, V., et al., Mediterranean diet and CHD: the Greek European Prospective Investigation into Cancer and Nutrition cohort. British Journal of Nutrition, 2012. 108(4): p. 699-709. | Wrong exposure |
| Dodin, S., et al., Flaxseed on cardiovascular disease markers in healthy menopausal women: a randomized, double-blind, placebo-controlled trial. Nutrition, 2008. 24(1): p. 23‐30. | Wrong intervention |
| Dodin, S., et al., The effects of flaxseed dietary supplement on lipid profile, bone mineral density, and symptoms in menopausal women: A randomized, double-blind, wheat germ placebo-controlled clinical trial. Journal of Clinical Endocrinology and Metabolism, 2005. 90: p. 1390-1397. | Wrong intervention |
| Domenech, M., et al., Effect of a walnut diet on office and 24-hour ambulatory blood pressure in elderly individuals: Findings from the WAHA randomized trial. Hypertension, 2019. 73: p. 1049-1057. | Population included in another larger included study |
| Doménech, M. et al., Mediterranean Diet Reduces 24-Hour Ambulatory Blood Pressure, Blood Glucose, and Lipids. Hypertension, 2014. 64(1): p. 69-76. | Wrong intervention |
| Ellsworth, J.L., L.H. Kushi, and A.R. Folsom, Frequent nut intake and risk of death from coronary heart disease and all causes in postmenopausal women: the Iowa Women's Health Study. Nutrition, metabolism, and cardiovascular diseases : NMCD, 2001. 11: p. 372-377. | Population included in more recent study |
| Estruch, R., et al., Effects of a Mediterranean-Style Diet on Cardiovascular Risk Factors a Randomized Trial. Annals of Internal Medicine, 2006. 145: p. 1-11. | Wrong intervention |
| Estruch, R., et al., Primary Prevention of Cardiovascular Disease with a Mediterranean Diet Supplemented with Extra-Virgin Olive Oil or Nuts. New England journal of medicine, 2018. 378(25): p. e34. | Wrong intervention |
| Fraser, G.E., K.D. Lindsted, and W.L. Beeson, Effect of risk factor values on lifetime risk of and age at first coronary event: The adventist health study. American Journal of Epidemiology, 1995. 142: p. 746-758. | Same study as Fraser 1992. |
| Fraser, G.E. and D.J. Shavlik, Risk factors for all-cause and coronary heart disease mortality in the oldest-old: The Adventist health study. Archives of Internal Medicine, 1997. 157: p. 2249-2258. | Subgroup of the population included in Fraser 1992. |
| Fraser et al. Associations between diet and cancer, ischemic heart disease, and all-cause mortality in non-Hispanic white California Seventh-day Adventists. Am J Clin Nutr, 1999. 70(Suppl): p. S532-38. | Review |
| Fung, T.T., et al., International food group-based diet quality and risk of coronary heart disease in men and women. American Journal of Clinical Nutrition, 2018. 107(1): p. 120-129. | Same population as Guasch-Ferré 2017 |
| He, K., et al., Dietary fat intake and risk of stroke in male US healthcare professionals: 14 Year prospective cohort study. British Medical Journal, 2003. 327: p. 777-781. | Population included in more recent study. |
| Hernáez, Á., et al., The Mediterranean Diet decreases LDL atherogenicity in high cardiovascular risk individuals: a randomized controlled trial. Molecular nutrition & food research, 2017. 61(9). | Wrong intervention |
| Hernandez-Alonso, P., et al., Effect of pistachio consumption on plasma lipoprotein subclasses in pre-diabetic subjects. Nutrition, Metabolism and Cardiovascular Diseases, 2015. 25: p. 396-402. | Wrong outcome  Same study as Hernandez-Alonso 2014. |
| Hu, F.B., et al., Frequent nut consumption and risk of coronary heart disease in women: Prospective cohort study. British Medical Journal, 1998. 317: p. 1341-1345. | Population included in more recent study. |
| Huguenin, G.V., et al., Improvement of antioxidant status after Brazil nut intake in hypertensive and dyslipidemic subjects. Nutrition journal, 2015. 14: p. 54. | Wrong intervention |
| Hutchins, A.M., et al., Daily flaxseed consumption improves glycemic control in obese men and women with pre-diabetes: A randomized study. Nutrition Research, 2013. 33: p. 367-375. | Wrong intervention |
| Jiang, R., et al., Nut and peanut butter consumption and risk of type 2 diabetes in women. Journal of the American Medical Association, 2002. 288: p. 2554-2560. | Population included in more recent study |
| Jones, J.B., et al., A randomized trial on the effects of flavorings on the health benefits of daily peanut consumption. American journal of clinical nutrition, 2014. 99(3): p. 490‐496. | Wrong intervention |
| Julibert, A., et al., Metabolic Syndrome Features and Excess Weight Were Inversely Associated with Nut Consumption after 1-Year Follow-Up in the PREDIMED-Plus Study. Journal of nutrition, 2020. 150(12): p. 3161‐3170. | Wrong outcome |
| Key, T.J., et al., Dietary habits and mortality in 11,000 vegetarians and health conscious people: results of a 17 year follow up. BMJ, 1996. 313(7060): p. 775-9. | Wrong exposure |
| Kushi., L.H. et al. Dietary Antioxidant Vitamins and Death from Coronary Heart Disease in Postmenopausal Women. N Engl J Med, 1996. 334(18): p. 1156-62. | Population included in more recent study |
| Larsson, S.C., A. Wolk, and M. Back, Dietary patterns, food groups, and incidence of aortic valve stenosis: A prospective cohort study. International Journal of Cardiology, 2019. 283: p. 184-188. | Wrong exposure |
| Liu et al. The effects of daily intake timing of almond on the body composition and blood lipid profile of healthy adults. 2017 | Same study as Liu et al. 2018, but shorter follow-up time |
| Liu, X., et al., Changes in nut consumption and subsequent cardiovascular disease risk among us men and women: 3 large prospective cohort studies. Journal of the American Heart Association, 2020. 9(7):e013877. | Wrong exposure (individual change in intake)  Same population as Guasch-Ferré, 2017. |
| Lucas, E.A., et al., Flaxseed improves lipid profile without altering biomarkers of bone metabolism in postmenopausal women. Journal of Clinical Endocrinology and Metabolism, 2002. 87: p. 1527-1532. | Wrong intervention |
| Meier, T., et al., Cardiovascular mortality attributable to dietary risk factors in 51 countries in the WHO European Region from 1990 to 2016: a systematic analysis of the Global Burden of Disease Study. European Journal of Epidemiology, 2019. 34: p. 37-55. | Wrong study design |
| Micha, R., et al., Association between dietary factors and mortality from heart disease, stroke, and type 2 diabetes in the United States. JAMA - Journal of the American Medical Association, 2017. 317: p. 912-924. | Wrong study design  Wrong outcome |
| Michels, A.J., et al., Daily consumption of Oregon hazelnuts affects alpha-tocopherol status in healthy older adults: A pre-post intervention study. Journal of Nutrition, 2018. 148: p. 1924-1930. | Wrong study design |
| Mitrou, P.N., et al., Mediterranean dietary pattern and prediction of all-cause mortality in a US population: Results from the NIH-AARP diet and health study. Archives of Internal Medicine, 2007. 167: p. 2461-2468. | Wrong exposure |
| Mohammadifard, N., et al., Long-term association of nut consumption and cardiometabolic risk factors. Nutrition, Metabolism and Cardiovascular Diseases, 2019. 29: p. 972-982 | Wrong exposure (individual change in intake) |
| Murie-Fernandez, M., et al., Carotid intima-media thickness changes with Mediterranean diet: a randomized trial (PREDIMED-Navarra). Atherosclerosis, 2011. 219(1): p. 158‐162. | Wrong outcome  Wrong intervention |
| Ndanuko, R.N., et al., Effect of individualised dietary advice for weight loss supplemented with walnuts on blood pressure: The HealthTrack study. European Journal of Clinical Nutrition, 2018. 72: p. 894-903. | Wrong intervention |
| Nettleton, J.A., et al., Dietary patterns and risk of incident type 2 diabetes in the multi-ethnic study of atherosclerosis (MESA). Diabetes Care, 2008. 31: p. 1777-1782. | Wrong exposure |
| Nettleton, J.A., et al., Incident Heart Failure Is Associated with Lower Whole-Grain Intake and Greater High-Fat Dairy and Egg Intake in the Atherosclerosis Risk in Communities (ARIC) Study. Journal of the American Dietetic Association, 2008. 108: p. 1881-1887. | Wrong outcome |
| O'Byrne, D.J., D.A. Knauft, and R.B. Shireman, Low fat-monounsaturated rich diets containing high-oleic peanuts improve serum lipoprotein profiles. Lipids, 1997. 32: p. 687-695. | Wrong study design |
| O’Connor, L.E., et al., Adherence to a Mediterranean-style eating pattern and risk of diabetes in a U.S. prospective cohort study. Nutrition and Diabetes, 2020. 10(1). | Wrong exposure |
| Pan, X.F., et al., Seventeen-year associations between diet quality defined by the health star rating and mortality in Australians: The Australian diabetes, obesity and lifestyle study (ausdiab). Current Developments in Nutrition, 2020. 4(11). | Wrong exposure |
| Papadaki, A., et al., Mediterranean diet and risk of heart failure: results from the PREDIMED randomized controlled trial. European journal of heart failure, 2017. 19(9): p. 1179‐1185. | Wrong outcome  Wrong intervention |
| Patade, A., et al., Flaxseed reduces total and LDL cholesterol concentrations in Native American postmenopausal women. Journal of Women's Health, 2008. 17: p. 355-366. | Wrong intervention |
| Qidwai, W., et al., Effectiveness, safety, and tolerability of powdered Nigella sativa (kalonji) seed in capsules on serum lipid levels, blood sugar, blood pressure, and body weight in adults: results of a randomized, double-blind controlled trial. Journal of alternative and complementary medicine, 2009. 15: p. 639-644. | Wrong intervention  Wrong duration |
| Rhee, Y. and A. Brunt, Flaxseed supplementation improved insulin resistance in obese glucose intolerant people: A randomized crossover design. Nutrition Journal, 2011. 10. | Wrong intervention |
| Riseberg, E., et al., Specific Dietary Protein Sources Are Associated with Cardiometabolic Risk Factors in the Boston Puerto Rican Health Study. Journal of the Academy of Nutrition and Dietetics., 2021. 15. | Wrong outcome |
| Ristic-Medic, D., et al., Effects of dietary milled seed mixture on fatty acid status and inflammatory markers in patients on hemodialysis. The Scientific World Journal, 2014. 2014. | Wrong population  Wrong study design |
| Rock, C.L., et al., Effects of pistachio consumption in a behavioral weight loss intervention on weight change, cardiometabolic factors, and dietary intake. Nutrients, 2020. 12: p. 1-14. | Wrong intervention |
| Salas-Salvadó, J., et al., Effect of a Mediterranean diet supplemented with nuts on metabolic syndrome status: one-year results of the PREDIMED randomized trial. Archives of internal medicine, 2008. 168(22): p. 2449‐2458. | Wrong outcome  Wrong intervention |
| Salas-Salvadó, J., et al., Prevention of diabetes with Mediterranean diets: A subgroup analysis of a randomized trial. Annals of Internal Medicine, 2014. 160(1): p. 1-10. | Wrong intervention |
| Schoufour, J.D., J.C. Kiefte-de Jong, and T. Voortman, Adherence to the 2015 Dutch dietary guidelines and risk of ten non-communicable diseases and mortality in the Rotterdam Study. Nederlands Tijdschrift voor Geneeskunde, 2018. 162(9): p. 19. | Wrong exposure |
| Shang, X., et al., Dietary protein from different food sources, incident metabolic syndrome and changes in its components: An 11-year longitudinal study in healthy community-dwelling adults. Clinical Nutrition, 2017. 36: p. 1540-1548. | Wrong outcome |
| Sisa, I., et al., Impact of diet on CVD and diabetes mortality in Latin America and the Caribbean: a comparative risk assessment analysis. Public health nutrition, 2021. 24: p. 2577-2591. | Wrong outcome |
| Sola, R., et al., Effect of a traditional Mediterranean diet on apolipoproteins B, A-I, and their ratio: a randomized, controlled trial. Atherosclerosis, 2011. 218(1): p. 174‐180. | Wrong intervention |
| Steffen, L.M., et al., A modified Mediterranean diet score is associated with a lower risk of incident metabolic syndrome over 25 years among young adults: the CARDIA (Coronary Artery Risk Development in Young Adults) study. The British journal of nutrition, 2014. 112: p. 1654-1661. | Wrong outcome |
| Steffen, L.M., et al., Associations of plant food, dairy product, and meat intakes with 15-y incidence of elevated blood pressure in young black and white adults: the Coronary Artery Risk Development in Young Adults (CARDIA) Study. The American journal of clinical nutrition, 2005. 82: p. 1169-1177. | Wrong outcome |
| Steffen, L.M., et al., Walnut consumption and cardiac phenotypes: The Coronary Artery Risk Development in Young Adults (CARDIA) study. Nutrition, Metabolism and Cardiovascular Diseases, 2021. 31: p. 95-101. | Wrong outcome |
| Tapsell, L.C., et al., Effect of interdisciplinary care on weight loss: A randomised controlled trial. BMJ Open, 2017. 7. | Wrong intervention |
| Tektonidis, T.G., et al., A Mediterranean diet and risk of myocardial infarction, heart failure and stroke: A population-based cohort study. Atherosclerosis, 2015. 243: p. 93-98. | Wrong exposure |
| Tharrey, M., et al., Patterns of plant and animal protein intake are strongly associated with cardiovascular mortality: The Adventist Health Study-2 cohort. International Journal of Epidemiology, 2018. 47: p. 1603-1612. | Wrong exposure |
| Toledo et al. Effect of the Mediterranean diet on blood pressure in the PREDIMED trial: results from a randomized controlled trial. BMC Medicine, 2013. 11:207. | Wrong intervention |
| Toulabi, T., et al., Effects of flaxseed on blood pressure, body mass index, and total cholesterol in hypertensive patients: A randomized clinical trial. Explore., 2021. | Wrong intervention |
| Trichopoulou, A., C. Bamia, and D. Trichopoulos, Anatomy of health effects of Mediterranean diet: Greek EPIC prospective cohort study. BMJ, 2009. 339: p. 26-28. | Wrong outcome  Wrong exposure |
| Tsaban, G., et al., The effect of green Mediterranean diet on cardiometabolic risk; A randomised controlled trial. Heart, 2021. 107: p. 1054-1061. | Wrong intervention |
| Voortman, T., et al., Adherence to the 2015 Dutch dietary guidelines and risk of non-communicable diseases and mortality in the Rotterdam Study. European Journal of Epidemiology, 2017. 32: p. 993-1005. | Wrong exposure |
| Wang, S., et al., Gut Microbiota Composition is Associated with Responses to Peanut Intervention in Multiple Parameters Among Adults with Metabolic Syndrome Risk. Molecular nutrition & food research, 2021: p. e2001051. | Wrong outcome  Same study as Wang D. et al |
| Watkins, T.R., et al., Improving atherogenic risk factors with flax seed bread. Vol. 37. 1995, Kenneth L. Jordan Heart Fund & Research Center, Montclair, NJ 07042, United States. 649-658. | Wrong exposure |
| Weng, L.C., et al., A diet pattern with more dairy and nuts, but less meat is related to lower risk of developing hypertension in middle-aged adults: The atherosclerosis risk in communities (ARIC) study. Nutrients, 2013. 5: p. 1719-1733. | Wrong outcome |
| Wien, M., et al., Almond consumption and cardiovascular risk factors in adults with prediabetes. Journal of the American College of Nutrition, 2010. 29: p. 189-197. | Wrong intervention |
| Wien, M., K. Oda, and J. Sabate, A randomized controlled trial to evaluate the effect of incorporating peanuts into an American Diabetes Association meal plan on the nutrient profile of the total diet and cardiometabolic parameters of adults with type 2 diabetes. Nutrition Journal, 2014. 13. | Wrong population |
| Wu, H., et al., Lifestyle counseling and supplementation with flaxseed or walnuts influence the management of metabolic syndrome. Journal of Nutrition, 2010. 140(11): p. 1937-1942. | Wrong intervention |
| Yari et al., Flaxseed Supplementation in Metabolic Syndrome Management: a Pilot Randomized, Open-labeled, Controlled Study. 2016 | Wrong intervention |
| 78. Yari, Z., et al., Combination therapy of flaxseed and hesperidin enhances the effectiveness of lifestyle modification in cardiovascular risk control in prediabetes: a randomized controlled trial. Diabetology and Metabolic Syndrome, 2021. 13. | Wrong intervention |
| Yari, Z., et al., Flaxseed Supplementation Improves Anthropometric measurements, Metabolic, and Inflammatory Biomarkers in Overweight and Obese Adults. International Journal for Vitamin and Nutrition Research., 2020. | Wrong intervention |
| Yari, Z., M. Cheraghpour, and A. Hekmatdoost, Flaxseed and/or hesperidin supplementation in metabolic syndrome: an open-labeled randomized controlled trial. European Journal of Nutrition, 2021. 60: p. 287-298. | Wrong intervention |
| Zare, H., The effects of consumption of walnut on serum lipids amount of normolipidemic and hyper lipidemic human. Biosciences Biotechnology Research Asia, 2010. 7: p. 1055-1058. | Wrong duration |
| Zaveri, S. and S. Drummond, The effect of including a conventional snack (cereal bar) and a nonconventional snack (almonds) on hunger, eating frequency, dietary intake and body weight. Journal of Human Nutrition and Dietetics, 2009. 22: p. 461-468. | Wrong study design |
| Zong, G., et al., Effects of flaxseed supplementation on erythrocyte fatty acids and multiple cardiometabolic biomarkers among Chinese with risk factors of metabolic syndrome. European Journal of Nutrition, 2013. 52: p. 1547-1551. | Wrong outcome |

### Supplementary Table 2. Characteristics of the included cohort studies^1^

| Reference | Country | Cohort name; population; study enrolment | Outcome(s) | Exposure  Consumption categories  Mean/median intake | Dietary assessment methods | Total sample size/person-years analysed | Follow-up time | Main covariates adjusted for | Funding |
| --- | --- | --- | --- | --- | --- | --- | --- | --- | --- |
| Al-Shaar et al. (2020) (73) | USA | HPFS; male; age 40-75 y; 1986- | CHD | Nuts (including tree nuts, peanuts, peanut butter) as substitute for red/processed meat (1 serving/day) | FFQ every 4 years | 43,272 (1,023,872 PY) | Up to 30 years | Age, calendar time, BMI, cigarette smoking, alcohol consumption, energy intake, family history of MI or stroke, multivitamin use, aspirin use, race or ethnicity, work status, profession, marital status, data on physical activity MET/week), other dietary variables. | Agency |
| Albert et al. (2002) (74) | USA | Physicians Health Study; male; age 40-84 y; 1982 | CHD | Nuts | FFQ | 21,454 (366,751 PY) | Mean 17 years | Previous CVD, BMI, smoking, history of diabetes, hypertension, hypercholesterolemia, alcohol consumption, vigorous exercise, aspirin use, beta-carotene use, vitamin E use, vitamin C use, multivitamin use, other dietary factors associated with nut intake. | Agency |
| Amba et al. (2019) (75) | USA | NIH-AARP; 50-71 y; 1995-96 | CVD mortality | Nuts (including peanuts, walnuts, seeds or other nuts, nut butters), g/1000 kcal  Mean total nut intake: 2.9±8.3 g/day  Mean peanut butter intake: 2.9±7.1 g/day | FFQ | 374,101 (5,453,103 PY) | Median 15.5 y | Energy intake, sex, age, BMI, race, smoking, alcohol consumption, education level, physical activity, white meat intake, red meat intake, vegetable intake, fruit intake, whole grain intake, use of vitamin supplements. | Agency |
| Asghari et al. (2017) (45) | Iran | Tehran Lipid and Glucose study; age ≥20 y; 2005-08 | T2D | Nuts, including peanuts, almonds, walnuts, pistachios, hazelnuts, sunflower seeds, watermelon seeds, pumpkin seeds, and compounds from them  Median intake: 1.19 (SEM 0.11) s/week | FFQ | 1984 | Mean 6.2 y | Age, total energy intake, gender, smoking status, years of education, BMI, waist circumference, blood pressure, fasting plasma glucose, serum cholesterol, TG, dietary intakes (foods, sugar, fibre, fats, carbohydrate, protein). | Agency |
| Bernstein et al. (2010) (76) | USA | NHS; female; age 30-55 y; 1980­­– | CHD | Nuts (including peanuts, walnuts and other nuts) | FFQ every 2-4 years | 84,136 | Up to 26 y | Energy intake, cereal fiber, alcohol, trans-unsaturated fatty acids, BMI, physical activity, cigarette smoking, menopausal status, parental history of early MI, years of multivitamin use, vitamin E use, aspirin use. | Mixed |
| Bernstein et al. (2012) (77) | USA | NHS, HPFS; age 30-75 y; 1980/1986– | Stroke | Nuts (including peanuts, walnuts and other nuts) | FFQ every 2-4 years | 127,160 | Up to 26 y (men 22 y, women 26 y) | Age, calendar time, dietary protein sources, energy intake, cereal fiber intake, alcohol intake, fruit/vegetables, trans unsaturated fatty acids, BMI, menopausal status, cigarette smoking, family history of MI, multivitamin use, vitamin E supplements, aspirin use. | Agency |
| Blomhoff et al. (2006) (78) | USA | Iowa Women’s Health Study; 1986 | CVD mortality, CHD mortality | Nuts/peanut butter  Mean intake: 2.37±3.44 s/week | FFQ | 31,788 | 15 y | Age, energy intake, BMI, waist/hip ratio, education, physical activity, use of estrogen, use of multivitamin supplements, alcohol, whole grain and refined grain intake, red meat, fish and seafood, total fruit and vegetables | Agency |
| Bonaccio et al. (2015) (79) | Italy | Moli-Sani; mean age ~54 y; 2005-10 | CVD mortality | Nuts (including walnuts, hazelnuts, almonds, peanuts) | FFQ | 19,386 (84,302 PY) | Median 4.3 y | Energy intake, physical activity, smoking, education, BMI, Mediterranean Diet Score, age, sex. | Agency |
| Buijsse et al. (2015) (101) | Multinational (European) | EPIC; mean age 52.2 y; 1991-99 | T2D | Nuts and seeds, g/day  Median intake: 0.6 g/day | FFQ | 14,939 | Median 12.3 y | Centre, sex, education, energy intake, smoking, alcohol intake, physical activity, BMI. | Agency |
| de Souza et al. (2020) (96) | Multinational | PURE; age 35-70 y; 2003-19 | CVD, MI, stroke | Nuts (tree nuts and ground nuts)  Mean 6.4±13.0 g/day | FFQ | 85,713 | Median 9.5 y | Location (urban vs. rural), age, sex, education, smoking, BMI, waist-to-hip ratio, physical activity, family history of CVD, diabetes or cancer, other dietary factors, total energy intake. | Mixed |
| di Giuseppe et al. (2015) (82) | Germany | EPIC-Potsdam; mean age 51 y; 1994-98 | Stroke | Nuts  Median intake (IQR) 0.82 (0.41-4.11) g/day | FFQ | 26,285 | Median 8.3 y | Age, sex, sports activity, alcohol consumption, smoking status, educational level, BMI, prevalent hypertension, hyperlipidemia, diabetes, dietary factors, total energy intake. | Agency |
| Djousse et al. (2010) | USA | Physicians’ Health Study; male; 40-84 y; 1982 | Stroke | Nuts; peanut butter | FFQ | 21,078 | Mean 21.1 y | Age, smoking, alcohol intake, prevalence of hypertension, diabetes, atrial fibrillation and CHD, aspirin use, BMI, exercise, consumption of breakfast cereal, dairy products, fruits and vegetables, fish, red meat. | Agency |
| Eslamparast et al. (2017) (81) | Iran | Golestan Cohort Study; age ≥40 y; 2004 | CVD mortality | Nuts, tree nuts, peanuts  Mean intake: 3.5 ±31.8 g/day in men, 2.6±9.5 g/day in women | FFQ | 49,112 | Median 7 y | Age, sex, BMI, level of education, place of residence, smoking status, opium and alcohol consumption, physical activity, wealth score, diabetes, hypertension, energy intake, intake of fish, red meat, chicken, fruit, vegetables, dairy products, egg and total fibre, magnesium, zinc and copper. | Agency |
| Fraser et al. (1992) (10) | USA | Adventist Health Study; age ≥25 y; non-Hispanic white; 1974-76 | CHD | Nuts | FFQ | 26,473 | 6 y | Age, sex, smoking, exercise, relative weight, hypertension, other foods and nondietary risk factors | Agency |
| Gopinath et al. (2015) (80) | Australia | Blue Mountains Eye study; age ≥49 y; 1992-94 | CVD mortality  IHD mortality  Stroke mortality | Nuts | FFQ | 2893 | 15 y | Age, sex, educational status, current smoking, BMI, total diet score, walking disability, doctor-diagnosed history of cancer, angina, acute MI, stroke, diabetes mellitus and hypertension, low-self-rated health. | Agency |
| Guasch-Ferré (2013) (84) | Spain | PREDIMED; age ≥55-80 y; high CVD risk; 2003-09 | CVD mortality | Nuts (including almonds, peanuts, hazelnuts pistachio, pine nuts, macadamia, Brazil nuts, walnuts) | FFQ, yearly | 7216 | Median 4.8 y | Age, sex, BMI, smoking status, educational level, physical activity, energy intake, history of diabetes, hypercholesterolemia, use of oral antidiabetic, antihypertensive drugs or statins, alcohol intake, food groups (vegetables, fruits, red meat, eggs, fish), adherence to Mediterranean diet. | Mixed |
| Guasch-Ferré et al. (2017) (42) | USA | NHS, HPFS; age 25-75 y; 1980/86– | CVD, CHD stroke | Nuts, peanuts, walnuts, peanut butter | FFQ, every 2-4 y | 210,836 (5,063,439 PY) | 32 y | Age, Caucasian (yes/no), BMI, physical activity, smoking status, multivitamin use, aspirin use, family history of diabetes, MI, cancer, history of diabetes, hypertension, hypercholesterolemia, energy intake, alcohol intake, red or processed meat, fruits and vegetable intake, menopausal status/hormone use in women; for peanut butter, also adjusted for glycemic load, white bread and soda intake. | Agency |
| Haring et al. (2014) (35) | USA | ARIC; 45-64 y; 1987-89 | CHD | Nuts, peanut butter  Median intake: 0.2 s/day | FFQ assessed 2 times | 12,066 | Median 22 y | Age, sex, race, study center, energy intake, smoking, education, systolic blood pressure, use of antihypertensive medication, HDL-C, total cholesterol, use of lipid lowering medication, BMI, waist-to-hip ratio, alcohol intake, physical activity, carbohydrate intake, fiber intake, magnesium intake. | Agency |
| Haring et al. (2015) (85) | USA | ARIC; 45-64 y; 1987-89 | Stroke | Nuts and peanut butter  Median intake: 0.21 s/day | FFQ | 11,601 | Median 22.7 y | Age, sex, race, study center, energy intake, smoking, cigarette years, education, systolic blood pressure, use of antihypertensive medication, HDL-C, total cholesterol, use of lipid lowering medication, BMI, waist-to-hip ratio, alcohol intake, physical activity, carbohydrate intake, fiber intake, fat intake, magnesium intake. | Agency |
| Hshieh et al. (2015) (86) | USA | Physicians’ Health Study; male; mean age 66.9 y; 1999-02 | CVD/CHD/Stroke mortality | Nuts and peanut butter  Median intake: 1 s/week. | FFQ | 20,742 | 9.6 y | Age, BMI, smoking, alcohol consumption, exercise, prevalent diabetes, prevalent hypertension, energy intake, intake of red meat, fruit & vegetables, saturated fat, magnesium and fiber. | Agency |
| Ibsen et al. (2020) (102) | Multinational (European) | EPIC; 1991– | T2D | Nuts (including tree nuts, peanuts, seeds, coconuts, chestnuts), nuts vs. red/processed meat.  Median intake: 1 g/day | Mostly FFQs | 26,460 | Median 12.3 y | Age, study center, energy intake, sex, education, physical activity, smoking status, alcohol intake, other dietary variables, BMI, history of hypertension, self-reported history of dyslipidemia, waist circumference. | Agency |
| Ikehara et al. (2021) (87) | Japan | Japan Public Health Center; age 45-74 y; 1990 | CVD, IHD, stroke | Peanuts | FFQ | 74,793 (994,604 PY) | Median 14.8 y | Age, sex, health centre, smoking status, alcohol consumption, perceived stress, physical activity, vegetables, fruit, fish soy, sodium, total energy intake, BMI, history of hypertension, diabetes, use of cholesterol-lowering drugs. | Agency |
| Imran et al. (2021) (88) | USA | Women’s Health Study; female; age >45 y; 1992-95 | CVD mortality | Nuts (peanuts and tree nuts) | FFQ | 39,167 | Mean 19 y | Age, smoking, BMI, alcohol, physical activity, postmenopausal status, family history of MI, marital status, AHEI score at baseline (excluding nuts). | Agency |
| Ivey et al. (2021) (89) | USA | Million Veterans Program; veterans (90 % men), mean age 64 y; 2011-18 | CVD mortality, CAD, stroke | Nuts, peanut butter | FFQ | 149,827 | Median 3.5 y | Age, sex, race, BMI, smoking status, alcohol intake, physical activity, education, modified DASH score. | Agency |
| Kochar et al. (2010) (90) | USA | Physician’s Health Study; male; age 40-84 y; 1982 | T2D | Nuts | FFQ | 20,224 | Mean 19.2 y | Age, exercise, hypertension, BMI, smoking, aspirin assignment, breakfast cereals, dairy, red meat consumption. | Agency |
| Larsson et al. (2018) (91) | Sweden | Cohort of Swedish Men, Swedish Mammography Cohort; middle-aged/ older; 1997 | MI, ischaemic stroke | Nuts (excluding coconuts or chestnuts) | FFQ | 61,364 | Mean 17 y | Age, sex, education, family history of MI, smoking, physical activity, aspirin use, consumption of alcohol, fruits, vegetables, total energy intake, BMI, history of diabetes or hypertension, hypercholesterolaemia. | Agency |
| Liu et al. (2021) (92) | USA | NHS, HPFS; age 30-75 y; 1980/1986– | CVD mortality | Walnuts | FFQ every 2-4 y | 93,340 (1628559 PY) | 20 y (mean 17.6 for women, 17.1 y for men) | Age, sex, race, smoking status, alcohol consumption, physical activity, multivitamin use, aspirin use, family history of diabetes, MI or cancer, menopausal status, hormone use, BMI, history of diabetes, hypertension or hypercholesterolemia, consumption of other nuts, consumption of other foods, total energy intake. | Agency |
| Luu et al. (2015) (43) | USA, China | Southern Community Cohort Study (US), Shanghai Women’s Health Study, Shanghai Men’s Health Study (China); age 40-79 y; 1996/2002 | CVD/  IHD/  Stroke/  Diabetes mortality | Nuts, peanuts, peanut butter  Mean intake:  SCCS:12.25 g/day  SWHS/SMHS (peanuts only): 2 g/day | FFQ | 206,029 (SCCS: 71,764, SMHS: 61,123, SWHS: 73,142) | Median   - SCCS: 5.4 y - SWHS: 12.2 y - SMHS: 6.5 y | Age, education, income, occupation, regular use of vitamin supplements, smoking status, alcohol consumption, BMI, comorbidity, physical activity, metabolic conditions, total energy intake, red meat, chicken, seafood, vegetable and fruit intake (tea drinking in SWHS/SMHS). | Agency |
| Mohammadifard et al. (2020) (93) | Iran | Isfahan Cohort Study; age ≥35 y; 2001 | CVD mortality | Nuts (including walnuts, almonds, pistachio, hazelnuts) | FFQ, repeated twice | 5432 (52,704,3 PY) | Median 13 y | BMI, age, sex, education, residence area, smoking status, physical activity, family history of CVD, diabetes, hypertension, hypercholesterolaemia, aspirin use, menopausal status, dietary factors. | Agency |
| Pan et al. (2013) | USA | NHS; female; age 35-77 y; 1980 | T2D | Nuts, walnuts, peanuts  Mean intake:   - Total nuts: 3.36 g/d - Walnuts: 0.56 g/d - Peanuts: 1.4 g/d | FFQ every 4 y | 137,956 | 10 y | Age, race, family history of diabetes, smoking status, alcohol intake, physical activity, postmenopausal status and menopausal hormone use, use of multivitamins, total energy intake, other dietary variables, BMI | Mixed |
| Parker et al. (2003) (68) | USA | Iowa Women’s health study; female; postmenopausal; 1986 | T2D | Nuts and peanut butter | FFQ | 35,988 | 12 y | Age, BMI, WHr, physical activity, current smoking status, alcohol consumption, total energy intake, education, current estrogen use, dietary factors | NI |
| Perez-Cornago et al. (2021) (95) | Multinational (European) | EPIC; age 35-70 y; 1992-2000 | IHD | Nuts and seeds (including nut and seed products, nut butter)  Median intake: 0.775 g/day | Mostly FFQs | 490,311 | 12.6 y | Age, smoking status, history of diabetes, previous hypertension, previous hyperlipidaemia, physical activity, employment status, level of education, alcohol consumption, BMI, total energy intake, red and processed meat, cheese intake. | Agency |
| Sun et al. (2021) (37) | USA | Women’s Health Initiative; female, postmenopausal; age 50-79 y; 1993-98 | CVD mortality | Nuts and seeds  Median intake: 0.2 oz-eq | FFQ | 102,521 | 18 y | Age, ethnicity, socioeconomic status, hormone use history, lifestyle, baseline health status, family history of heart attack/stroke, dietary factors including energy intake, fatty acids etc., mutual adjustment from other protein sources, BMI. | Agency |
| Tong et al. (2020) (36) | Multinational (European) | EPIC; mean age 52 y in men, 50.4 y in women; 1992-2000 | Stroke | Nuts and seeds  Median intake: 0.8 g/day | Mostly FFQs | 418,329 | Mean 12.7 y | Age, smoking status, prevalence of diabetes, hypertension or hyperlipidaemia, physical activity, employment status, level of education, alcohol consumption, BMI, energy intake, dietary fibre, all foods, mean systolic blood pressure, non-HDL-cholesterol. | Agency |
| Van den Brandt et al. (2019) (98) | Netherlands | The Netherlands cohort study; age 55-69 y; 1986-96 | CVD mortality | Nuts  Mean intake: 6.4 g/day | FFQ | 12,025 (30320 PY) | 10 y | Age, sex, cigarette smoking, years of smoking, history of hypertension and diabetes, height, BMI, physical activity, level of education, intake of alcohol, vegetables and fruit, energy intake, use of nutritional supplements, postmenopausal hormone replacement therapy. | NI |
| van den Brandt & Schouten (2015) (97) | Netherlands | The Netherlands cohort study; age 55-69 y; 1986-96 | CVD/  IHD/  Stroke mortality | Nuts, tree nuts, peanuts, peanut butter  Mean intake:  Nuts:   - Men: 8.1 g/day - Women: 4.4 g/day   Peanut butter:   - Men: 1.4 g/day - Women: 1.2 g/day | FFQ | 3693 | 10 y | Age, sex, cigarette smoking, years of smoking, history of hypertension and diabetes, body height, BMI, physical activity, highest level of education, intake of alcohol, vegetables and fruit, energy intake, use of nutritional supplements, postmenopausal hormone replacement therapy. | Agency |
| Villegas et al. (2008) (70) | China | Shanghai Women’s Health study; female; age 40-70 y; 1997-2000 | T2D | Peanuts  Mean intake: 1.5 g/day, median 0.7 g/day | FFQ at baseline and first follow-up | 64,191 (297,744,3 PY) | Mean 4.6 y | Age, BMI, WHr, total energy intake, fiber intake, vegetable intake, income level, education, occupation, physical activity, smoking status, alcohol consumption status, presence of hypertension at baseline, menopausal status. | Agency |
| Von Ruesten et al. (2013) (71) | Germany | EPIC-Potsdam; age 35-65 y; 1994-98 | CVD, T2D | Nuts (including peanuts, walnuts, paranuts etc.)  Median intake: 0.8 g/day. | FFQ | 23,531 | 8 y | Age, sex, smoking status, pack-years of smoking, alcohol consumption, WHr, BMI, physical activity, education, use of vitamin supplements, non-consumption of the respective food group, total energy intake, prevalent hypertension and history of high blood lipids. | Agency |
| Wang et al. (2016) (99) | China | Linxian Nutrition Intervention Trial; age 40-69 y; 1984-91 | CHD/  Stroke mortality | Nuts (including peanuts, chestnuts, walnuts)  Median intake: 0.3 s/month | FFQ | 2445 | 26 y | Age, sex, commune, smoking, drinking, season of interview, BMI. | Agency |
| Wurtz et al. (2021) (100) | USA | NHS, HPFS; age 25-75 y; 1980/1986– | T2D | Nuts, peanuts, peanut butter, walnuts (replacing red meat), | FFQ every 4 y | 148,853 (2,113,245 PY) | 4 to 8 8-year periods | Age, calendar time, total energy intake, marital status, race, family history of diabetes, hypertension and hypercholesterolaemia, BMI, alcohol intake, modifed aHEI score, smoking status, physical activity, menopausal status and use of postmenopausal hormones, intake of red meat and other protein foods, weight change. | Agency |
| Yaemsiri et al. (2012) (72) | USA | Women’s Health Initiative; female, postmenopausal; age 50-79 y; 1994-98 | Ischaemic stroke | Nuts | FFQ at baseline and 3-y follow-up | 87,025 (663,041 PY) | Mean 7.6 y | Energy intake, other dietary factors, age, race, education, income, hormone replacement therapy use, smoking, physical activity, alcohol intake, history of CHD, Afib, diabetes, aspirin use, use of antihypertensive medications, use of cholesterol-lowering medications, BMI, blood pressure. | Agency |
| Yamakawa et al. (2021) (44) | Japan | Takayama study; age ≥35 y; 1992 | CVD mortality | Nuts, peanuts  Mean intake, total nuts:   - Men: 1.8 g/d - Women: 1.5 g d   Mean intake, peanuts:   - Men: 1.4 g/d - Women: 1.2 g/d | FFQ | 410353 PY | Mean 13.7 y in men, 14.4 y in women | Age, sex, marital status, education, BMI, history of diabetes, history of hypertension, smoking status, alcohol intake, physical activity, use of vitamin supplements, total energy intake, intake of vegetables, fruits and red meat, menopausal status. | Agency |

1 Abbreviations: ARIC: Atherosclerosis Risk in Communities, CAD: Coronary artery disease, CHD: Coronary heart disease, CVD: cardiovascular disease, EPIC: European Prospective Investigation into Cancer and Nutrition. FFQ: Food frequency questionnaire; HPFS: Health Professionals’ Follow-up Study, IHD: Ischaemic heart disease, MI: myocardial infarction, NHS: Nurses’ Health Study, PREDIMED: Prevención con Dieta Mediterránea, PURE: Prospective Urban and Rural Epidemiology study, PY: person-years, RoB: risk of bias; SCCS: Southern Southern Community Cohort Study. SMHS: Shanghai Men’s Health Study, SWHS: Shanghai Women’s Health Study, T2D: Type 2 diabetes.

### Supplementary Table 3: Associations between nuts and seeds consumption and risk of cardiovascular disease in cohort studies^1^.

| Reference | Outcome(s) | Outcome assessment | Number of events | Effect measure | Effect estimates (95 % CI) | RoB |
| --- | --- | --- | --- | --- | --- | --- |
| Amba et al. (2019) (75) | CVD mortality | Registry | Nuts: 17,262  Peanut butter: 17,684 | HR | Nuts:  Never: 1  T1: 0.78 (0.73-0.82)  T2: 0.72 (0.68-0.76)  T3: 0.70 (0.66-0.74)  p trend <0.001  Per 2.15 g/day = 0.99 (0.98-1.00)  Peanut butter:  Never: 1  T1: 0.90 (0.90-0.98)  T2: 0.96 (0.91-1.01)  T3: 0.99 (0.95-1.04)  p trend = 0.021  Per 1.7 g/day = 1.01 (1.00-1.01) |  |
| Blomhoff et al. (2006) (78) | CVD mortality | Linkage with state registry | CVD deaths: 1675 | HR | 0: 1  <1 s/week: 1.00 (0.86, 1.17)  1-4 s/week: 0.84 (0.73, 0.96)  ≥5 s/week: 0.72 (0.60, 0.88) |  |
| Bonaccio et al. (2015) | CVD mortality | National death registry | CVD deaths: 104 | HR | ≥2 s/month: 0.87 (0.57-1.32) |  |
| de Souza et al. (2020) | CVD | Physician-adjudicated | CVD: 5979 (multivariable-adjusted: 4487)  CVD deaths: 2039 (multivariable-adjusted: 1253) | HR | CVD events:  <30 g/month: 1.00  30 g/month - <30 g/week: 1.02 (0.94, 1.11)  30 g/week - <120 g/week: 0.98 (0.90, 1.06)  ≥120 g/week: 0.91 (0.81, 1.02)  p for trend = 0.14  CVD mortality:  <30 g/month: 1.00  30 g/month - <30 g/week: 1.01 (0.87, 1.19)  30 g/week - <120 g/week: 0.96 (0.82, 1.13)  ≥120 g/week: 0.72 (0.56, 0.92)  p for trend = 0.048 |  |
| Eslamparast et al. (2017) | CVD mortality | Autopsy reports, medical records | 2016 | HR | Never: 1  <1 s/week: 0.87 (0.79-0.97)  1-<3 s/week: 0.75 (0.63-0.89)  ≥3 s/week: 0.77 (0.58-1.01)  p for trend = 0.018 |  |
| Gopinath et al. (2015) | CVD mortality | National death registry or information from family members | CVD deaths: 546 | HR | T1: 1  T2: 0.76 (0.61-0.94)  T3: 0.90 (0.73-1.12)  p for trend = 0.43 |  |
| Guasch-Ferré (2013) | CVD mortality | Self-reported, family physicians, medical records and national death registry | CVD deaths: 81 | HR | Total nuts:  Never: 1  1-3 s/week: 0.42 (0.24-0.74)  >3 s/week: 0.45 (0.25-0.81)  p for trend = 0.091  Walnuts:  Never: 1  1-3 s/week: 0.41 (0.23-0.73)  >3 s/week: 0.53 (0.29-0.98)  p for trend = 0.047  Nuts ex walnuts:  Never: 1  1-3 s/week: 0.74 (0.45-1.23)  >3 s/week: 0.42 (0.20-0.89)  p for trend = 0.031 |  |
| Guasch-Ferré et al. (2017) | CVD | Self-reported, confirmed by medical records, national death registry | 14,136 | HR | Total nuts:  Never: 1  <1 s/week: 0.91 (0.86-0.95)  1 s/week: 0.90 (0.85-0.95)  2-4 s/week 0.86 (0.81-0.91)  ≥5 s/week 0.86 (0.79-0.93)  p for trend = 0.0002  Per 28 g/day: 0.94 (0.89-0.99)  Peanuts:  Never: 1  <1 s/week 0.92 (0.88-0.95)  1 s/week: 0.94 (0.88-1.00)  ≥2 s/week: 0.87 (0.82-0.93)  P for trend: 0.0002  Per 28 g/day: 0.91 (0.83-1.00)  Walnuts:  Never: 1  <1 s/week 0.95 (0.89-1.02)  ≥1 s/week: 0.81 (0.71-0.92)  P for trend: <0.001  Per 28 g/day: 0.71 (0.52-0.97)  Peanut butter:  Never: 1  <1 s/week: 0.98 (0.93-1.02)  1 s/week: 1.01 (0.96-1.07)  ≥2 s/week: 0.99 (0.94-1.04)  P for trend: 0.74  Per 28 g/day: 0.99 (0.94-1.05) |  |
| Hshieh et al. (2015) | CVD mortality | Self-reported, confirmed by medical records | CVD deaths: 760 | HR | <1 s/month = 1  1-3 s/month = 0.98 (0.82, 1.17)  1 s/week = 0.89 (0.72, 1.11)  2-4 s/week = 0.80 (0.62, 1.03)  ≥5 s/week = 0.74 (0.55, 1.02)  p for trend = 0.015 |  |
| Ikehara et al. (2021) | CVD | Medical records reviewed by physicians | Combined stroke and IHD: 4448 | HR | Q1: 1.00  Q2: 0.89 (0.81-0.98)  Q3: 0.93 (0.86-1.01)  Q4: 0.87 (0.80-0.94)  p for trend = 0.004 |  |
| Imran et al. (2021) | CVD mortality | Hospital records, death certificates, autopsy reports | CVD deaths: 959 | HR | Never: 1.00  1-3 s/month: 0.93 (0.76-1.14)  1 s/week: 0.84 (0.69-1.01)  ≥2 s/week: 0.73 (0.61-0.87)  p for trend = 0.0004 |  |
| Ivey et al. (2021) | CVD mortality | Health records, national death registry | CVD deaths: 1311 | HR | <1/Month: 1  1-3/Month: 0.95 (0.82, 1.10)  1/Week: 0.79 (0.66, 0.94)  2-4/Week: 0.74 (0.62, 0.89)  ≥5/Week: 0.76 (0.63, 0.93)  p for trend: 0.04 |  |
| Liu et al. (2021) | CVD mortality | Self-reported, vital records, national death registry, adjudicated by physician | Females: 3219  Males: 2263 | HR | Never/almost never: 1  <1 s/week: 0.97 (0.89, 1.04)  1 s/week: 0.98 (0.86, 1.11)  2-4 s/week: 0.86 (0.73, 1.00)  ≥5 s/week: 0.75 (0.62, 0.92)  p for trend = 0.001  Per 0,5 serving increase: 0.86 (0.79, 0.94) |  |
| Luu et al. (2015) | CVD | Registry, home visits (in SWHS/SMHS) | CVD deaths: 4444 | HR | African-Americans:  Q1: 1  Q2: 0.85 (0.72-1.00)  Q3: 0.82 (0.68-0.99)  Q4: 0.81 (0.68-0.97)  Q5: 0.77 (0.63-0.92)  p for trend = 0.03  European-Americans  Q1: 1  Q2: 0.80 (0.61-1.06)  Q3: 0.74 (0.56-0.97)  Q4: 0.66 (0.49-0.87)  Q5: 0.62 (0.46-0.84)  p for trend = 0.02  Shanghai:  Q1: 1  Q2: 0.82 (0.72--0.93)  Q3: 0.75 (0.67-0.84)  Q4: 0.69 (0.62-0.77)  Q5: 0.76 (0.67-0.85)  p < 0.001 |  |
| Mohammadifard et al. (2020) | CVD mortality | Hospital records, mortality database, adjudicated by specialist panel | CVD deaths: 179 | HR | Q1: 1  Q2: 1.04 (0.67-1.60)  Q3: 0.91 (0.58-1.44)  Q4: 0.55 (0.30-0.98)  p-trend = 0.08 |  |
| Sun et al. (2021) | CVD mortality | Death certificates, medical reecords, autopsy reports, national death registry | CVD deaths: 6993 | HR | Q1: 1  Q2: 0.97 (0.90, 1.04)  Q3: 0.98 (0.91, 1.06)  Q4: 0.94 (0.87, 1.01)  Q5: 0.94 (0.98, 1.01)  p for trend = 0.06  Per 1 oz equivalent/day = 0.98 (0.94, 1.02) |  |
| Van den Brandt & Schouten (2015) | CVD | Linkage wit Dutch Central Bureau of Statistics | CVD deaths: 2985 | HR | Total nuts:  0 g: 1  0.1-<5 g: 0.89 (0.76-1.03)  5-<10 g: 0.74 (0.59-0.91)  ≥10 g: 0.83 (0.69-1.00)  p for trend = 0.013  Tree nuts:  0 g: 1  0.1-<5 g: 0.91 (0.78-1.07)  ≥5 g: 0.91 (0.68-1.23)  p for trend = 0.252  Peanuts:  0 g: 1  0.1-<5 g: 0.86 (0.74-0.99)  ≥5 g: 0.78 (0.66-0.93)  p for trend = 0.003  Peanut butter:  0 g: 1  0.1-<5 g: 1.00 (0.85-1.17)  ≥5 g: 0.99 (0.80-1.23)  p for trend = 0.956 |  |
| Van den Brandt et al. (2019) | CVD mortality | Linkage with Dutch Central Bureau of Statistics | CVD deaths:2985 | HR | 0: 1  <10g: 0.85 (0.74-0.98)  <20g: 0.83 (0.66-1.05)  ≥20g: 0.84 (0.66-1.07)  p for trend = 0.202  Per 50 g/day increase: 0.75 (0.57-1.00)  Per 50 g/day replacing processed meat: 0.62 (0.44-0.88) |  |
| Von Ruesten et al. (2013) | CVD | Self-reported, confirmed by physicians, cancer registries or death certificates | CVD: 363 | HR | Per 5 g/day: 1.00 (0.93-1.07) |  |
| Yamakawa et al. (2021) | CVD mortality | Death certificates | Males: 775  Females: 903 | HR | Total nuts:  Males:  Q1: 1  Q2: HR 4 (0.66-1.06)  Q3: 0.95 (0.74-1.22)  Q4: 0,87 (0.68-1.11)  p for trend = 0.457  Females:  Q1: 1  Q2: 0.84 (0.66-1.05)  Q3: 0.88 (0.69-1.13)  Q4: 0.79 (0.62-1.01)  p for trend = 0.122  Peanuts:  Males:  Q1: 1  Q2: 0.88 (0.70-1.11)  Q3: 0.94 (0.73-1.21)  Q4: 0.88 (0.69-1.12)  p for trend = 0.422  Females:  Q1: 1  Q2: 0.75 (0.60-0.95)  Q3: 0.81 (0.63-1.04)  Q4: 0.72 (0.56-0.92)  p for trend = 0.036 |  |

^1^ Abbreviations: CVD: cardiovascular disease, HR: hazard ratio, RoB: risk of bias.

### Supplementary Table 4: Associations between nuts and seeds consumption and risk of coronary heart disease in cohort studies^1^

| Reference | Outcome(s) | Outcome assessment | Number of events | Effect measure | Effect estimates with 95 % CI | RoB |
| --- | --- | --- | --- | --- | --- | --- |
| Al-Shaar et al. (2020) | CHD | Self-reported, confirmed by medical records or death records. | 4456 | HR | 1 s/day replacing total red meat: 0.89 (0.82-0.96)  1 s/day replacing unprocessed red meat: 0.89 (0.81-0.98)  1 s/day replacing processed red meat: 0.85 (0.77-0.94) | Serious |
| Albert et al. (2002) | CHD | Self-reported, confirmed by medical records | CHD deaths: 566  Nonfatal MI: 1037 | RR | CHD deaths:  <1/month: 1  1-3/month: 0.89 (0.67-1.16)  1/week: 0.90 (0.67-1.22)  ≥2/week: 0.70 (0.50-0.98)  p trend = 0.06  Nonfatal MI:  <1/month: 1  1-3/month: 1.22 (1.00-1.51)  1/week: 1.20 (0.96-1.50)  ≥2/week: 1.04 (0.82-1.33)  p trend = 0.87 | Serious |
| Bernstein et al. (2010) | CHD | Self-reported, confirmed by medical records or death records | CHD: 3162  Nonfatal MI: 2210  Deaths: 952 | RR | Q1: 1  Q2: 0.73 (0.65, 0.82)  Q3: 0.91 (0.82, 1.00)  Q4: 0.76 (0.67, 0.84)  Q5: 0.68 (0.60, 0.76)  p for trend: <0.0001  Per 1 s/day: 0.78 (0.66, 0.93)  Per 1 s/day replacing red meat: 0.70 (0.58-0.23) | Moderate |
| Blomhoff et al. (2006) | CHD mortality | Linkage with state registry | CHD deaths: 948 | HR | 0: 1  <1 s/week: 1.03 (0.84, 1.26)  1-4 s/week: 0.82 (0.68, 0.98)  ≥5 s/week: 0.71 (0.55, 0.91 | Serious |
| Bonaccio (2015) | CHD mortality | National death registry | CHD deaths: 39 | HR | ≥2 s/month:  0.74 (0.38, 1.45) | Serious |
| de Souza et al. (2020) | MI | Physician-adjudicated | 2559 (multivariable-adjusted: 1788) | HR | <30 g/month: 1  30 g/month - <30 g/week: 0.97 (0.85, 1.12)  30 g - <120 g/week: (0.99 (0.87, 1.13)  ≥120 g/week: 0.86 (0.72, 1.04)  Per 30 g/day: 0.92 (0.81, 1.07) | Moderate |
| Fraser et al. (1992) | CHD | Self-reported, confirmed by medical records, church records, state and national death index | Definite fatal CHD: 260  Definite nonfatal MI: 134 | RR | Fatal CHD:  <1 s/week = 1  1-4 s/week = 0.76 (0.56-1.04)  ≥5 s/week = 0.52 (0.36-0.76)  Nonfatal MI:  <1 s/week = 1  1-4 s/week = 0,78 (0,51-1,18)  ≥5 s/week = 0,49 (0,28-0,85) | Serious |
| Guasch-Ferré et al. (2017) | CHD | Self-reported, confirmed by medical records, national death registry | 8390 | HR | Total nuts:  Never: 1  <1 s/week 0.88 (0.83-0.94)  1 s/week: 0.83 (0.78-0.90)  2-4 s/week: 0.82 (0.76-0.88)  ≥5 s/week: 0.80 (0.72-0.89)  p for trend: <0.0001  Per 28 g/day: 0.87 (0.81-0.94) | Serious |
| Haring et al. (2014) | CHD | Self-reported, interviews, hospital records, death certificates, physician-completed questionnaire | 1147 | HR | Q1: 1  Q2: 0.89 (0.75, 1.06)  Q3: 0.86 (0.71, 1.05)  Q4: 0.83 (0.68, 1.01)  Q5: 0.91 (0.74, 1.12)  p for trend = 0.67  Per 1 s/day replacing red meat: 0.81 (0.65-1.02)  Per 1 s/day replacing processed meat: 0.94 (0.79-1.10) | Moderate |
| Hshieh et al. (2015) | CAD mortality | Self-reported, confirmed by medical records | CAD deaths: 405 | HR | <1 s/month = 1  1-3 s/month = 1.02 (0.80, 1.30)  1 s/week = 0.88 (0.66, 1.19)  2-4 s/week = 0.72 (0.50, 1.04)  ≥5 s/week = 0.85 (0.56, 1.28)  p for trend = 0.083 | Serious |
| Ikehara et al. (2021) | IHD | Medical records reviewed by physicians | 849 | HR | Q1: 1.00  Q2: 0.98 (0.79-1.21)  Q3: 0.93 (0.77-1.11)  Q4: 0.97 (0.80-1.17)  p for trend = 0.81 | Serious |
| Ivey et al. (2021) | CAD | Health records, national death registry | 9908 | HR | <1/month: 1  1-3/Month: 0.93 (0.89, 0.99)  1/Week: 0.89 (0.84, 0.95)  2-4/Week: 0.83 (0.78, 0.89)  ≥5/Week: 0.78 (0.72, 0.84)  P for trend < 0,0001 | Serious |
| Larsson et al. (2018) | MI | National patient registry and cause-of-death registry | MI: 4983  Fatal MI: 917  Ischaemic stroke: 3782 | HR | MI  None: 1  1-3 s/month: 0.98 (0.92-1.04)  1-2 s/week: 0.91 (0.79-1.05)  ≥3 s/week: 0.88 (0.70-1.11)  p for trend: 0.12  Per serving: 0.98 (0.95-1.02)  Fatal MI:  None: 1  1-3 x/month: 0.85 (0.73-0.99)  1-2 x/week: 0.75 (0.52-1.09)  ≥3 x/week: 1.34 (0.88-2.05)  p for trend: 0.12  Per serving: 1.02 (0.96-1.09)  Nonfatal MI:  None: 1.0  1-3 s/month: 1.01 (0.95-1.08)  1-2 s/week: 0.95 (0.82-1.11)  ≥3 s/week: 0.76 (0.58-1.01)  Per serving: 0.97 (0.93-1.01) | Moderate |
| Luu et al. (2015) | IHD | Registry, home visits (in SWHS/SMHS) | IHD deaths: 1424 | HR | African-Americans:  Q1: 1  Q2: 0.67 (0.51-0.88)  Q3: 0.95 (0.72-1.25)  Q4: 0.74 (0.55-0.98)  Q5: 0.62 (0.45-0.85)  p for trend = 0.01  European-Americans:  Q1: 1  Q2: 0.85 (0.59-1.24)  Q3: 0.73 (0.50-1.06)  Q4: 0.65 (0.44-0.97)  Q5: 0.60 (0.39-0.92)  p = 0.007  Shanghai:  Q1: 1  Q2: 0.93 (0.72-1.00)  Q3: 0.76 (0.60-0.97)  Q4: 0.75 (0.60-0.94)  Q5: 0.70 (0.54-0.89)  p = 0.001 | Moderate |
| Perez-Comago et al. (2021) | IHD | Combination of self-reported and record linkage with registries | 7880 | HR | Observed intake:  Q1: 1  Q2: 0.99 (0.90-1.08)  Q3: 0.97 (0.91-1.04)  Q4: 0.96 (0.89-1.03)  Q5: 0.93 (0.86-1.01)  Per 10 g/day calibrated intake:  0.90 (0.82-0.98), p-trend = 0.02 | Serious |
| Van den Brandt & Schouten (2015) | IHD | Linkage with Dutch Central Bureau of Statistics | IHD deaths: 1488 | HR | Total nuts:  0 g: 1  0.1-<5 g: 0.90 (0.76-1.07)  5-<10 g: 0.67 (0.52-0.88)  10+ g: 0.83 (0.67-1.04)  p for trend = 0.026  Tree nuts:  0 g: 1  0.1-<5 g: 0.88 (0.74-1.06)  ≥5 g: 1.03 (0.72-1.46)  p for trend = 0.440  Peanuts:  0 g: 1  0.1-<5 g: 0.86 (0.73-1.03)  ≥5 g: 0.79 (0.64-0.96)  p for trend = 0.014  Peanut butter:  0 g: 1  0.1-<5 g: 1.04 (0.86-1.26)  ≥5 g: 0.97 (0.75-1.24)  p for trend = 0.968 | Moderate |
| Wang et al. (2016) | CHD | Doctor visits, hospital records | CHD deaths: 355 | HR | Per 3 s/month: 0.89 (0.82-0.98) | Serious |

^1^ Abbreviations: CAD: coronary artery disease, CHD: coronary heart disease, IHD: ischaemic heart disease, MI: myocardial infarction, HR: hazard ratio, RR: risk ratio, RoB: risk of bias.

### Supplementary Table 5: Associations between nuts and seeds consumption and risk of stroke in cohort studies^1^

| Reference | Outcome(s) | Outcome assessment | Number of events | Effect measure | Effect estimates with 95 % CI | RoB |
| --- | --- | --- | --- | --- | --- | --- |
| Bernstein et al. (2012) | Stroke | Self-reported, confirmed by medical records or death records | Stroke: 4030  Ischaemic stroke: 2212 | RR | Total stroke:  Q1: 1  Q2: 0.94 (0.85, 1.04)  Q3: 0.93 (0.83, 1.03)  Q4: 0.99 (0.89, 1.09)  Q5: 0.88 (0.79, 0.98)  Per 1 s/day: 0.82 (0.66, 1.01)  Ischaemic stroke:  Q1: 1  Q2: 0.97 (0.84, 1.11)  Q3: 1.00 (0.86, 1.15)  Q4: 1.03 (0.89, 1.18)  Q5: 0.97 (0.84, 1.12)  Per 1 s/day: 0.93 (0.70, 1.22)  Per 1 s/day replacing red meat: 0.83 (0.73, 0.96) | Serious |
| Bonaccio et al. (2015) | Stroke mortality | National death registry | Stroke deaths: 19 | HR | ≥2 s/month:  0.98 (0.36-2.66) | Serious |
| de Souza et al. (2020) | Stroke | Physician-adjudicated | 2915 (multivariable-adjusted: 2385) | HR | <30 g/month: 1  30 g/month - <30 g/week: 1.03 (0.91, 1.16)  30 g - <120 g/week: 0.99 (0.88, 1.11)  ≥120 g/week: 0.98 (0.84, 1.14)  Per 30 g/day: 0.93 (0.84, 1.04) | Moderate |
| di Giuseppe et al. (2015) | Stroke | Self-reported, confirmed by physicians and hospital records | Total: 288  Nonfatal: 252  Fatal stroke: 36 | HR | Total stroke:  Non-consumers: 1.56 (1.17-2.08)  1/2 s/week: 1 (ref)  1/2-1 s/week: 1.06 (0.75-1.52)  >1 x/week: 1.37 (0.92-2.05)  p for tend linear = 0.9  Nonfatal:  Non-consumers: 1.48 (1.09-2.01)  1/2 s/week: 1 (ref)  1/2-1 s/week: 1.04 (0.71-1.53)  >1 s/week: 1.47 (0.97-2.24)  p for trend = 0.71  Fatal:  Non-consumers: 2.18 (1.00-4.78)  1/2 s/week: 1 (ref)  1/2-1 s/week: 1.22 (0.47-3.18)  >1 s/week: 0.67 (0.15-2.97)  p linear = 0.43  Ischemic stroke:  Non-consumers: 1.50 (1.09-2.07)  1/2 x/week: 1 (ref)  1/2-1 s/week: 1.16 (0.79-1.72)  >1 s/week: 1.62 (1.05-2.49)  p for trend = 0.57 | Serious |
| Djousse et al. (2010) | Stroke | Self-reported, confirmed by medical records, death certificates | Total stroke: 1424  Ischaemic stroke: 1189 | HR | Total stroke:  0 : 1  <1 s/week = 0.91 (0.79-1.05)  1 s/week = 0.95 (0.81-1.11)  2-4 s/week = 0.90 (0.75-1.08)  5-6 s/week = 1.11 (0.85-1.46)  ≥7 s/week = 1.07 (0.79-1.46)  p for trend = 0.12  Ischaemic stroke:  0 = 1  <1 s/week = 0.86 (0.74-1.01)  1 s/week = 0.94 (0.79-1.11)  2-4 s/week = 0.97 (0.80-1.18)  5-6 s/week = 1.06 (0.79-1.43)  ≥7 s/week = 0.93 (0.65-1.34)  p for trend = 0.30 | Serious |
| Guasch-Ferré et al. (2017) | Stroke | Self-reported, confirmed by medical records, national death registry | 5910 | HR | Stroke:  Total nuts  Never: 1  <1 s/week: 0.95 (0.88-1.03)  1 s/week: 1.01 (0.92-1.10)  2-4 s/week: 0.95 (0.87-1.04)  ≥5 s/week: 0.98 (0.86-1.13)  P for trend: 0.88  Per 28 g/day: 1.02 (0.96-1.08) | Serious |
| Haring et al. (2015) | Stroke | Hospital records, adjudicated by physician reviewers | Total stroke: 699  Ischaemic stroke: 596 | HR | Total stroke:  Q1: 1  Q2: 0.83 (0.65, 1.05)  Q3: 1.03 (0.81, 1.32)  Q4: 1.04 (0.81, 1.33)  Q5: 1.00 (0.77, 1.31)  p for trend = 0.42  Ischaemic stroke:  Q1: 1  Q2: 0.75 (0.58, 0.98)  Q3: 1.09 (0.84, 1.42)  Q4: 1.00 (0.76, 1.31)  Q5: 1.01 (0.76, 1.34)  p for trend = 0.36 | Moderate |
| Hshieh et al. (2015) | Stroke mortality | Self-reported, confirmed by medical records | Stroke deaths: 142 | HR | Stroke deaths:  <1 s/month = 1  1-3 s/month = 0.91 (0.60, 1.39)  1 s/week = 0.82 (0.50, 1.36)  2-4 s/week = 0.84 (0.48, 1.47)  ≥5 s/week = 0.64 (0.32, 1.30)  p for trend = 0.205 | Moderate |
| Ikehara et al. (2021) | Stroke | Medical records reviewed by physicians | Stroke: 3599  Ischaemic stroke: 2223 | HR | Stroke:  Q1: 1.00  Q2: HR 0.87 (0.79-0.96)  Q3: 0.93 (0.85-1.01)  Q4: 0.84 (0.77-0.93)  p for trend = 0.002  Ischemic stroke:  Q1: 1.00  Q2: HR 0.82 (0.72-0.93)  Q3: 0.86 (0.77-0.96)  Q4: 0.80 (0.71-0.90)  p for trend = 0.002 | Serious |
| Ivey et al. (2021) | Stroke | Health records, national death registry | Stroke: 3570  Ischemic stroke: 3570 | HR | Stroke:  <1/month: 1.00  1-3/month: 0.90 (0.83, 0.99)  1/week: 0.85 (0.77, 0.95)  2-4/week: 0.80 (0.72, 0.89)  ≥5/week: 0.81 (0.72, 0.92)  p for trend = 0.002  Ischaemic stroke:  <1 s/month: 1  1-3 s/month: 0.91 (0.83, 1.00)  1 s/week: 0.84 (0.76, 0.93)  2-4 s/week: 0.80 (0.71, 0.89)  ≥5 s/week: 0.81 (0.71, 0.92)  p for trend: 0.005 | Serious |
| Larsson et al. (2018) | Ischaemic stroke | National patient registry and cause-of-death registry | 3782 | HR | None: 1.00  1-3 x/month: 1.04 (0.97-1.11)  1-2 x/week:1.12 (0.96-1.31)  ≥3 x/week: 1.03 (0.80-1.32))  p for trend: 0.29  Per serving: 1.02 (0.99-1.06) | Moderate |
| Luu et al. (2015) | Stroke mortality | Registry, home visits (in SWHS/SMHS) | Ischaemic stroke deaths: 709 | HR | African-Americans:  Q1: 1  Q2: 0.89 (0.49-1.62)  Q3: 0.72 (0.35-1.46)  Q4: 0.85 (0.44-1.64)  Q5: 0.89 (0.45-1.74)  p = 0.35  European-Americans:  Q1: 1  Q2: 0.39 (0.10-1.55)  Q3: 0.38 (0.19-1.46)  Q4: 0.43 (0.12-1.54)  Q5: 0.47 (0.12-1.76)  p = 0.38  Shanghai:  Q1: 1  Q2: 0.89 (0.68-1.15)  Q3: 0.79 (0.62-1.01)  Q4: 0.67 (0.52-0.85)  Q5: 0.77 (0.60-0.100)  p = 0.003 | Moderate |
| Tong et al. (2020) | Stroke | Combination of self-reported or record linkage with registries | Total stroke: 6748  Ischaemic stroke: 3772 | HR | Total stroke:  Q1: 1  Q2: 0.86 (0.78-0.96)  Q3: 0.89 (0.82-0.95)  Q4: 0.91 (0.84-0.99)  Q5: 0.87 (0.80-0.95)  p for trend = 0.83  Per unit increase (10 g/day), observed intake: 1.00 (0.97-1.03)  Per unit increase, calibrated intake: 0.93 (0.85-1.02)  Ischaemic stroke:  Q1: 1  Q2: 0.89 (0.77-1.03)  Q3: 0.90 (0.82-0.99)  Q4: 0.92 (0.82-1.03)  Q5: 0.94 (0.84-1.06)  p for trend = 0.44  Per unit increase (10 g/day), observed intake: 1.02 (0.97-1.06)  Per unit increase, calibrated intake: 0.97 (0.86-1.10) | Serious |
| Van den Brandt & Schouten (2015) | Stroke mortality | Linkage with Dutch Central Bureau of Statistics | Stroke deaths: 565 | HR | Total nuts:  0 g: 1  0.1-<5 g: 0.80 (0.63-1.01)  5-<10 g: 0.68 (0.48-0.97)  ≥10 g: 0.876 (0.56-1.02)  p for trend = 0.029  Tree nuts:  0 g: 1  0.1-<5 g: 0.90 (0.70-1.15)  ≥5 g: 0.74 (0.44-1.24)  p for trend = 0.186  Peanuts:  0 g: 1  0.1-<5 g: 0.79 (0.62-0.99)  ≥5 g: 0.71 (0.54-0.94)  p for trend = 0.010  Peanut butter:  0 g: 1  0.1-<5 g: 0.84 (0.64-1.11)  ≥5 g: 0.86 (0.60-1.23)  p for trend = 0.222 | Moderate |
| Wang et al. (2016) | Stroke mortality | Doctor visits, hospital records | Stroke deaths: 193 | HR | Per 3 s/month: 0.99 (0.93, 1.05) | Serious |
| Yaemsiri et al. (2012) | Ischaemic stroke | Self-reported and adjudicated locally and centrally | 1049 | HR | Per 1 s/day: 0.89 (0.66-1.20) | Moderate |

^1^ Abbreviations: HR: hazard ratio, RR: risk ratio, RoB: risk of bias.

### Supplementary Table 6: Associations between nuts and seeds consumption and risk of type 2 diabetes in cohort studies^1^

| Reference | Outcome(s) | Outcome assessment | Number of events | Effect measure | Effect estimate with 95 % CI | RoB |
| --- | --- | --- | --- | --- | --- | --- |
| Asghari et al. (2017) | T2D | ADA criteria (fasting glucose ≥126 mg/dl, or 2h plasma glucose ≥200 mg/dl during OGTT, or receiving treatment/diabetic medications | 150 | OR | <1 s/week: 1  1-1.99 s/week: 1.11 (0.72-1.71)  2-3.99 s/week: 0.51 (0.26-0.97)  ≥4 s/week: 0.47 (0.25-0.90)  p for trend = 0.01 | Serious |
| Amba et al (2019) | T2D mortality | Registry | T2D deaths: 661 | HR | Never: 1  T1: 1.01 (0.73-1.38)  T2: 0.92 (0.67-1.27)  T3: 0.95 (0.69-1.31)  p for trend = 0.74  Per 2.15 g/day: 0.96 (0.90-1.02) | Serious |
| Buijsse et al. (2015) | T2D | Self-reported, linkage to registers and hospital data | 11,059 | HR | Non-consumers: 1.11 (1.01, 1.22)  T1: 1.01 (0.92, 1.11)  T2: 1 (ref)  T3: 0.93 (0.79, 1.10)  p for trend = 0.51 | Serious |
| Ibsen et al. (2020) | T2D | Self-reported, medical records, registries | 11,741 | HR | Per 1 s/day (10 g) replacing red/processed meat: 0.90 (0.84-0.96) | Serious |
| Kochar et al. (2010) | T2D | Self-reported | 1828 | HR | Rarely/never: 1  <1 s/week: 1.06 (0.93-1.20)  1 s/week: 1.10 (0.95-1.26)  2-4 s/week: 0.97 (0.82-1.14)  5-6 s/week: 0.99 (0.76-1.30)  ≥7 s/week: 0.87 (0.61-1.24)  p for trend = 0.99 | Serious |
| Luu et al. (2015) | T2D mortality | Registry, home visits (in SWHS/SMHS) | T2D deaths: 777 | HR | African-Americans:  Q1: 1  Q2: 0.81 (0.56-1.19)  Q3: 0.84 (0.56-1.26)  Q4: 0.78 (0.52-1.17)  Q5: 0.55 (0.35-0.88)  p = 0.11  European-Americans:  Q1: 1  Q2: 1.00 (0.48-2.08)  Q3: 0.65 (0.29-1.46)  Q4: 1.35 (0.68-2.67)  Q5: 0.76 (0.34-1.70)  p = 0.80  Shanghai:  Q1: 1  Q2: 0.83 (0.62-1.13)  Q3: 0.60 (0.43-0.82)  Q4: 0.91 (0.69-1.18)  Q5: 0.98 (0.74-1.30)  p = 0.73 | Moderate |
| Pan et al. (2013) | T2D | Self-reported | 5930 | HR | Total nuts:  Never/rarely: 1  <1/week: 0.99 (0.94-1.05)  1/week: 1.03 (0.96-1.10)  2-4/week: 0.99 (0.90-1.09)  ≥5week: 1.01 (0.90)  P for trend= 0.95  Per 2 servings/week: 1.00 (0.97-1.03)  Peanuts:  Never/rarely: 1  <1/week: 1.02 (0.97-1.06)  1/week: 1.07 (0.99-1.17)  ≥2/week: 1.04 (0.93-1.16)  p -trend = 0.42  Per 2 servings/week: 1.02 (0.97-1.07)  Tree nuts:  Never/rarely: 1  <1/week: 1.02 (0.97-1.07)  1/week: 1.05 (0.96-1.15)  ≥2week: 0.98 (0.87-1.10)  P for trend= 0.44  Per 2 servings/week: 1.02 (0.97-1.07)  Walnuts:  Never/rarely: 1  <1/week:0.96 (0.90-1.02)  1/week: 0.87 (0.75-1.01)  ≥2/week: 0.76 (0.62-0.94)  p-trend = 0.002  Per 2 servings/week:  0.85 (0.77-0.94)  Other tree nuts:  Never/rarely: 1  <1/week: 1.01 (0.95-1.08)  1/week:1.01 (0.90-1.13)  ≥2/week: 1.04 (0.92-1.18)  p-trend= 0.49  Per 2 servings/week:  1.02 (0.97-1.07) | Serious |
| Parker et al. (2003) | T2D | Self-reported | 1831 | RR | Nuts:  <1/month: 1  <1/week: 0.98 (0.87- 1.10)  1-4/week: 1.06 (0.93-1.22)  ≥5/week: 57 (1.51 (1.13-2.04)  p for trend = 0.004  Peanut butter:  <1/month: 1  <1/week: 0.96 (0.84-1.09)  1-4/week: 0.99 (0.88-1.12)  ≥5/week: 0.97 (0.80-1.18)  p for trend 0.86 | Serious |
| Von Ruesten et al. (2013) | T2D | Self-reported, confirmed by physicians, cancer registries or death certificates | 837 | HR | Per 5 g/day: 0.95 (0.90-1.00) | Serious |
| Wurtz et al. (2021) | T2D | Self-reported | 8763 | HR | Per 1 s/day increase replacing red meat: 0.85 (0.80, 0.91)  Per 1 s/day increase replacing unprocessed red meat: 0.86 (0.79, 0.93)  Per 1 s/day increase replacing processed red meat: 0.79 (0.71, 0.87) | Serious |

^1^ Abbreviations: HR: hazard ratio, OR: odds ratio, RR: risk ratio, RoB: risk of bias, T2D: type 2 diabetes

### Supplementary Table 7: Results from randomized controlled trials on blood lipids^1^

| Reference | Outcome(s) | Baseline values | Endpoint values | Mean change |
| --- | --- | --- | --- | --- |
| Al Abdrabalnabi et al. (2020) | TG  HDL-C | TG: 102.6 ± 46.5 mg/dl  HDL-C: 56.4 ± 14.9 mg/dl | TG:  I: 91.1 (87.1, 95.3)  C: 89.4 (85.3, 93.7)  p = 0.484  HDL-C:  I: 50.0 (48.8, 51.3)  C: 52.4 (51.0, 53.8)  p = 0.836 | TG:  I: -0.94 (-0.91, -0.98) mg/dl  C: -0.96 (-0.93, -0.99) mg/dl  HDL-C:  I: -0.95 (-0.93, -0.96)  C: -0.95 (-0.93, -0.97) |
| Barbour et al. (2015) | Total-C  LDL-C  HDL-C  TG | Total-C: 5.1 ± 0.8 mmol/l  LDL-C: 3.3 ± 0.7 mmol/l  HDL-C: 1.4 ± 0.3 mmol/l  TG: 1.2 ± 0.6 mmol/l | Total-C:  I: 5.2 ± 0.8 mmol/l  C: 5.1 ± 0.8 mmol/l  p = 0.662  LDL-C:  I: 3.6 ± 0.8 mmol/l  C: 3.5 ± 0.8 mmol/l  p = 0.421  HDL-C:  I: 1.4 ± 0.3 mmol/l  C: 1.4 ± 0.4 mmol/l  p = 0.190  TG:  I: 1.2 ± 0.7 mmol/l  C: 1.3 ± 0.6 mmol/l  p = 0.129 |  |
| Bashan (2018) | Total-C  LDL-C  HDL-C  TG | Total-C:  I: 249.2 ± 39.16 mg/dl  C: 251.5 ± 27.54 mg/dl  LDL-C:  I: 152.2 ± 15.92 mg/dl  C: 152.2 ± 16.31 mg/dl  HDL-C:  I: 47.0 ± 10.16 mg/dl  C: 48.5 ± 9.57 mg/dl  TG:  I: 249.6 ± 72.61 mg/dl  C: 247.5 ± 68.16 mg/dl | Total-C:  I: 218.5 ± 32.98 mg/dl  C: 236.5 ± 22.03 mg/dl  p < 0.001  LDL-C:  I: 129.9 ± 13.27 mg/dl  C: 145.1 ± 15.21 mg/dl  p < 0.001  HDL-C:  I: 51.6 ± 10.27 mg/dl  C: 48.6 ± 9.30 mg/dl  p = 0.125  TG:  I: 185.1 ± 53.46 mg/dl  C: 213.0 ± 58.43 mg/dl  p < 0.001 | Total-C:  I: -12.31 %  C: -5.96 %  LDL-C:  I: -14.65 %  C: -4.66 %  HDL-C:  I: +9.78 %  C: +2.08 %  TG:  I: -25.84 %  C: -13.93 % |
| Casas-Agustench (2011) | Total-C  LDL-C  HDL-C  TG | Total-C:  I: 5.38 ± 0.79 mmol/l  C: 5.82 ± 1.30 mmol/l  LDL-C:  I: 3.45 ± 0.71 mmol/l  C: 3.79 ± 1.03 mmol/l  HDL-C:  I: 1.17 ± 0.29 mmol/l  C: 1.12 ± 0.26 mmol/l  TG:  I: 1.53 ± 0.70 mmol/l  C: 1.69 ± 0.95 mmol/l | Total-C:  I vs. C: +0.38 (-0.03, 0.79) mmol/l  p = 0.071  LDL-C:  I vs. C: +0.29 (-0.01, 0.11) mmol/l  p = 0.058  HDL-C:  I vs. C: -0.01 (-0.12, 0.11) mmol/l  p = 0.923  TG:  I vs. C: +0.06 (-0.37, 0.05)  p = 0.785 | Total-C:  I: -0.16 (-0.42, 0.11) mmol/l  C: -0.48 (-0.83, -0.14) mmol/l  LDL-C:  I: -0.13 (-0.34, 0.08) mmol/l  C: -0.36 (-0.62, -0.10)  HDL-C:  I: -0.02 (-0.1, 0.06)  C: -0.02 (-0.11, 0.06)  TG:  I: -0.02 (-0.23, 0.18)  C: -0.07 (-0.45, 0.32) |
| Coates et al. (2020) | Total-C  LDL-C  HDL-C  TG | Total-C:  I: 5.10 (s.e. 0.13) mmol/l  C: 5.25 (s.e. 0.11) mmol/l  LDL-C:  I: 3.08 (SEM 0.12) mmol/l  C: 3.27 (SEM 0.11) mmol/l  HDL-C:  I: 1.23 (SEM 0.04) mmol/l  C: 1.46 (SEM 0.04) mmol/l  TG:  I: 1.29 (SEM 0.07) mmol/l  C: 1.16 (SEM 0.07) mmol/l | Total-C:  I: 4.91 (SEM 0.11) mmol/l  C: 5.21 (SEM 0.11) mmol/l  p = 0.15  LDL-C:  I: 2.94 (SEM 0.10) mmol/l  C: 3.19 (SEM 0.10) mmol/l  p = 0.13  HDL-C:  I: 1.44 (SEM 0.04) mmol/l  C: 1.47 (SEM 0.04) mmol/l  p = 0.571  TG:  I: 1.15 (SEM 0.06) mmol/l  C: 1.18 (SEM 0.06) mmol/l  p = 0.548 |  |
| Gulati et al. (2014) | Total-C  LDL-C  HDL-C  TG | Total-C:  I: 189.4 ± 34.5 mg/dl  C: 195.4 ± 36.1 mg/dl  LDL-C:  I: 108.3 ± 23.7 mg/dl  C: 111.4 ± 27.7 mg/dl  HDL-C:  I: 33.5 ± 5.1 mg/dl  C: 36.3 ± 5.9 mg/dl  TG:  I: 196.8 ± 93.6 mg/dl  C: 171.1 ± 67.4 mg/dl | Total-C:  I: 174.5 ± 19 mg/dl  C: 185.1 ± 18.9 mg/dl  *P =* 0.02  LDL-C:  I: 98.8 ± 13.2 mg/dl  C: 107.7 ± 13.2 mg/dl  p = 0.006  HDL-C:  I: 38.1 ± 5.4 mg/dl  C: 37.9 ± 5.4 mg/dl  p = 0.9  TG:  I: 153.5 ± 36.5 mg/dl  C: 159.7 ± 36.5 mg/dl  p = 0.5 |  |
| Hernandez-Alonso (2014) | Total-C  LDL-C  HDL-C  TG | Total-C:  I: 217.44 (208.10, 226.79) mg/dl  C: 213.83 (205.48, 222.19) mg/dl  LDL-C:  I: 137.93 (128.18, 147.67) mg/dl  C: 136.77 (128.85, 144.70) mg/dl  HDL-C:  I: 54.28 (50.43, 58.13) mg/dl  C: 54.42 (51.00, 57.83) mg/dl  TG:  I: 125.81 (111.07, 140.56) mg/dl  C: 113.89 (101.70, 126.07) mg/dl |  | Total-C:  I: -3.74 (-9.20, 1.72) mg/dl  C: +2.11 (-4.41, 8.63) mg/dl  LDL-C:  I: -4.00 (-9.03, 1.03) mg/dl  C: +1.20 (-4.35, 6,75) mg/dl  HDL-C:  I: +1.33 (-1.65, 4.32)  C: +1.34 (-0.71, 3.39)  TG:  I: -4.96 (-15.72, 5.79)  C: +7.47 (-7.87, 22.81) |
| Hunter et al. (2021) | Total-C  LDL-C  HDL-C  TG | Total-C:  I: 154 (SEM 3) mg/dl  C: 159 (SEM 3) mg/dl  LDL-C:  I: 93 (SEM 3) mg/dl  C: 97 (SEM 3) mg/dl  HDL-C:  I: 42 (SEM 1) mg/dl  C: 43 (SEM 1) mg/dl  TG:  I: 96 (SEM 5) mg/dl  C: 91 (SEM 5) mg/dl | Total-C:  I: 150 (SEM 3) mg/dl  C: 158 (SEM 3) mg/dl  p = 0.33  LDL-C:  I: 89 (SEM 3) mg/dl  C: 96 (SEM 3) mg/dl  p = 0.51  HDL-C:  I: 41 (SEM 1) mg/dl  C: 45 (SEM 1) mg/dl  p = 0.04  TG:  I: 98 (SEM 5) mg/dl  C: 89 (SEM 5) mg/dl  p = 0.53 |  |
| Hwang et al. (2019) | Total-C  LDL-C  HDL-C  TG | Total-C:  I: 185.17 ± 30.63 mg/dl  C: 191.78 ± 34.61 mg/dl  LDL-C:  I: 121.74 ± 28.09 mg/dl  C: 116.38 ± 28.51 mg/dl  HDL-C:  I: 42.36 ± 9.32 mg/dl  C: 41.61 ± 9.37  TG:  I: 137.93 ± 64.83 mg/dl  C: 124.11 ± 56.47 mg/dl | Total-C:  I: 178.28 ± 30.34  C: 187.15 ± 32.01  p = 0.633  LDL-C:  I: 113.07 ± 23.63  C: 118.76 ± 29.71  p = 0.866  HDL-C:  I: 43.86 ± 10.03  C: 41.76 ± 9.55  p = 0.028  TG:  I: 117.74 ± 51.78  C: 135.21 ± 58.23  p = 0.219 |  |
| Kasliwal et al. (2015) | Total-C  LDL-C  HDL-C  TG | Total-C:  I: 198.9 ± 35.8 mg/dl  C: 209.1 ± 28.6 mg/dl  LDL-C:  I: 137.2 ± 32.6  C: 141.1 ± 21.1  HDL-C:  I: 35.7 ± 8.8  C: 43.4 ± 11.1  TG:  I: 151.9 ± 68.3  C: 155.1 ± 61.6 | Total-C:  I: 193.7 ± 41.5  C: 208.9 ± 34.4 mg/dl  p = 0.12  LDL-C:  I: 127.6 ± 34.0 mg/dl  C: 137.0 ± 26.2 mg/dl  p = 0.36  HDL-C:  I: 37.8 ± 10.1 mg/dl  C: 43.1 ± 8.6 mg/dl  p = 0.23  TG:  I: 140.8 ± 63.1 mg/dl  C: 143.5 ± 64.3 mg/dl p = 0.2 |  |
| Liu et al. (2018) | Total-C  LDL-C  HDL-C  TG | Total-C:  I: 178.09 ± 29.87 mg/dl  C: 163.48 ± 31.37 mg/dl  LDL-C:  I: 112.65 ± 27.05 mg/dl  C: 96.37 ± 29.37 mg/dl  HDL-C:  I: 57.48 ± 12.38 mg/dl  C: 62.22 ± 16.2 mg/dl  TG:  I: 94.56 ± 50.73 mg/dl  C: 65.0 ± 21.85 mg/dl | Total-C:  I: 160.33 ± 27.92 mg/dl  C: 155.48 ± 28.78 mg/dl p = 0.009  LDL-C:  I: 100.33 ± 26.54 mg/dl  C: 91.22 ± 26.73 mg/dl p = 0.06  HDL-C:  I: 53.15 ± 11.85 mg/dl  C: 60.74 ± 12.06 mg/dl p = 0.359  TG:  I: 72.0 ± 40.29 mg/dl  C: 56.96 ± 21.1 mg/dl p = 0.08 | Total-C:  I: -17.65 ± 21.36 mg/dl  C: -8 ± 17.57 mg/dl  LDL-C:  I: -12.31 ±18.59 mg/dl  C: -5.15 ± 14.38 mg/dl  HDL-C:  I: -4.33 ± 8.83 mg/dl  C: -1.48 ± 12.31 mg/dl  TG:  I: -22.56 ± 43.94 mg/dl  C: -8.04 ± 25.89 mg/dl |
| Madan et al. (2021) | Total-C  LDL-C  HDL-C  TG | Total-C:  I: 151.4 ± 31.19 mg/dl  C: 138.46 ± 23.73  LDL-C:  I: 93.33 ± 28.04 mg/dl  C: 80.68 ± 21.10 mg/dl  HDL-C:  I: 43.6 ± 14.39 mg/dl  C: 42.66 ± 9.58 mg/dl  TG:  I: 75.54 ± 37.99 mg/dl  C: 75.12 ± 35.53 mg/dl |  | Total-C:  I: -5.7 (-10.42, -0.98) mg/dl  C: -13.35 (-4.38, 31.09) mg/dl p = 0.04  LDL-C:  I: -4.27 (-9.05, 0.51) mg/dl  C: +5.93 (1.95, 9.91) mg/dl p = 0.01  HDL-C:  I: -1.07 (-3.61, 1.46) mg/dl  C: -0.55 (-2.25, 1.13) mg/dl p = 0.73  TG:  I: -2.74 (-9.42, 3.94)  C: +1.7 (-4.25, 7.65) p = 0.32 |
| Njike et al. (2015), calorie-adjusted | Total-C  LDL-C  HDL-C  TG | Total-C:  I: 212.7 ± 33.8 mg/dl  C: NI  LDL-C:  I: 129.2 ± 31.2 mg/dl  C: NI  HDL-C:  I: 60.8 ± 16.7 mg/dl  C: NI  TG:  I: 115 ± 46.7 mg/dl  C: NI |  | Total-C:  I: -16.04 ± 27.34 mg/dl  C: -9.42 ± 19.85 mg/dl p = 0.15  LDL-C:  I: -14.52 ± 24.11 mg/dl  C: -9.72 ± 15.87 mg/dl p = 0.22  HDL-C:  I: -1.33 ± 7.95 mg/dl  C: -0.12 ± 8.35 mg/dl p = 0.45  TG:  I: -1.15 ± 34.34 mg/dl  C: +2.44 ± 39.60 mg/dl  p = 0.69 |
| Njike et al. (2015), ad libitum | Total-C  LDL-C  HDL-C  TG | Total-C:  I: 206.2 ± 34.0 mg/dl  C: NI  LDL-C:  I: 126.1 ± 31.1 mg/dl  C: NI  HDL-C:  I: 58.5 ± 14.9 mg/dl  C: NI  TG:  I: 110.2 ± 53.3  C :NI |  | Total-C:  I: -12.51 ± 22.49 mg/dl  C: -11.14 ± 21.78 mg/dl p = 0.77  LDL-C:  I: -12.39 ± 17.82 mg/dl  C: -11.84 ± 19.10 mg/dl p = 0.89  HDL-C:  I: -1.08 ±6.83 mg/dl  C: -0.24 ± 8.96 mg/dl p = 0.99  TG:  I: +4.53 ± 53.69 mg/dl  C: +4.57 ± 38.89 mg/dl p = 0.99 |
| Tey et al. (2013) | Total-C  LDL-C  HDL-C  TG | Total-C:  I (30g): 4.92 ± 0.17 mmol/l  C: 4.93 ± 0.17 mmol/l  LDL-C:  I (30g): 3.07 ± 0.15 mmol/l  C: 3.03 ± 0.14 mmol/l  HDL-C:  I (30 g): 1.26 ± 0.04 mmol/l  C: 1.32 ± 0.05 mmol/l  TG:  I (30 g): 1.29 ± 0.08 mmol/l  C: 1.27 ± 0.07 mmol/l | Total-C:  I (30g): 4.78 ± 0.16 mmol/l  C: 4.91 ± 0.16 mmol/l  LDL-C:  I (30g): 2.93 ± 0.15 mmol/l  C: 3.05 ± 0.15 mmol/l  HDL-C:  I (30 g): 1.30 ± 0.04 mmol/l  C: 1.34 ± 0.06 mmol/l  TG:  I (30 g): 1.19 ± 0.07 mmol/l  C: 1.13 ± 0.05 mmol/l |  |
| Torabian et al. (2010) | Total-C  LDL-C  HDL-C  TG | Total-C: 5.72 ± 0.83 mmol/l  LDL-C: 3.35 ± 0.88 mmol/l  HDL-C: 1.53 ± 0.39 mmol/l  TG: 1.39 ± 0.77 mmol/l | Total-C:  I: 5.41 ± 0.09 mmol/l  C: 5.54 + 0.09 mmol/l p = 0.01  LDL-C:  I: 3.29 ± 0.09 mmol/l  C: 3.38 + 0.09 mmol/l  p = 0.06  HDL-C:  I: 1.5 ± 0.04 mmol/l  C: 1.51 ± 0.04 mmol/l p = 0.72  TG:  I: 1.35 ± 0.05 mmol/l  C: 1.44 ± 0.07 mmol/l p = 0.01 |  |
| Wang, D. et al. (2021) | Total-C  LDL-C  HDL-C  TG | Total-C:  I: 4.68 ± 0.84 mmol/l  C: 4.72 ± 0.97 mmol/l  LDL-C:  I: 3.54 ± 088 mmol/l  C: 3.53 ± 0.97 mmol/l  HDL-C:  I: 1.10 ± 0.24 mmol/l  C: 1.14 ± 0.25 mmol/l  TG:  I: 1.58 (1.46, 1.72) mmol/l  C: 1.57 (1.43, 1.72) mmol/l | Total-C:  I: 4.70 ± 0.86 mmol/l  C: 4.75 ± 0.96 mmol/l  p = 0.966  LDL-C:  I: 3.59 ± 0.91 mmol/l  C: 3.54 ± 0.91 mmol/l p = 0.689  HDL-C:  I: 1.15 ± 0.25 mmol/l  C: 1.19 ± 0.27 mmol/l p = 0.917  TG:  I: 1.51 (1.38, 1.65) mmol/l  C: 1.59 (1.45, 1.75) mmol/l p = 0.150 |  |
| Wang, J et al. (2021) | Total-C  HDL-C  TG | Total-C:  I: 169 (SEM 6.2) mg/dl  C: 173 (SEM 7.1) mg/dl  HDL-C:  I: 34 (SEM 1.2) mg/dl  C: 32 (SEM 1.5) mg/dl  TG:  I: 94 (SEM 5.9) mg/dl  C: 84 (SEM 5.3) mg/dl | Total-C:  I: 163 ± 5.6 mg/dl  C: 169 ± 7.8 mg/dl  HDL-C:  I: 33 ± 1.1 mg/dl  C: 32 ± 1.8 mg/dl  TG:  I: 85 ± 4.8 mg/dl  C: 84 ± 5.8 mg/dl |  |
| Wang, X et al. (2012) | Total-C  LDL-C  TG | Total-C:  I: 5.29 ± 1.01) mmol/l  C: 5.01 ± 0.99 mmol/l  LDL-C:  I: 3.08 ± 0.70 mmol/l  C: 2.70 ± 0.90 mmol/l  TG:  I: 2.47 ± 1.28 mmol/l  C: 2.09 ± 0.78 mmol/l | Total-C:  I: 5.20 ± 0.94 mmol/l  C: 5.15 ± 1.04 mmol/l  LDL-C:  I: 3.10 ± 0.69 mmol/l  C: 2.99 ± 0.86 mmol/l  TG:  I: 2.14 ± 1.03 mmol/l  C: 1.88 ± 0.89 mmol/l | Total-C:  I: -0.01 ± 0.67 mmol/l  C: +0.14 ± 0.56 mmol/l  LDL-C:  I: +0.12 ± 0.50 mmol/l  C: +0.29 ± 0.74 mmol/l  TG:  I: -0.38 ± 0.79 mmol/l  C: -0.21 ± 0.53 mmol/l |

^1^Abbreviations: HDL-C: HDL-cholesterol, LDL-C: LDL-cholesterol, TG: triglycerides, Total C: total cholesterol, C: control group, I: intervention group.

### Supplementary Table 8: Results from randomized controlled trials on blood pressure^1^.

| Reference | Baseline values, mmHg | Endpoint values, mmHg | Mean change, mmHg |
| --- | --- | --- | --- |
| Al-Abdrabalnabi (2020) | SBP:  I: 126.9 (125.3, 128.5)  C: 128.0 (126.4, 129.7)  DBP:  I: 77.8 (76.9, 78.8)  C: 78.2 (77.3, 79.2) | SBP:  I: 125.6 (123.9, 127.3)  C: 128 (126.3, 129.8) p = 0.265  DBP:  I: 77.1 (76.1, 78.1)  C: 78.2 (77.1, 79.2) p = 0.369 | SBP:  I: -1.3 (-2.9, 0.31)  C: +0.01 (-1.7, 1.67)  DBP:  I: -0.71 (-1.68, 0.26)  C: -0.08 (-1.07, 0.92) |
| Casas-Agustench (2011) | SBP:  I: 145 ± 15  C: 137 ± 19  DBP:  I: 86 ± 8  C: 82 ± 10 | SBP:  I vs. C: +4.5 (-2.6, 11.6) p = 0.208  DBP:  I vs. C: +2.0 (-2.2, 6.3)  P = 0.337 | SBP:  I: -6 (-11, -2)  C: -3 (-5, -1)  DBP:  I: -10 (-16, -5)  C: -4 (-8, -0.7) |
| Coates et al. (2020) | SBP:  I: 133 (SEM 1)  C: 132 (SEM 1)  DBP:  I: 77 (SEM 1)  C: 76 (SEM 1) | SBP:  I: 128 (SEM 2)  C: 131 (SEM 2) p = 0.55  DBP:  I: 75 (SEM 1)  C: 76 (SEM 1) p = 0.965 |  |
| Hernandez-Alonso et al. (2014) | SBP:  I: 133.89 (129.75, 138.03)  C: 80.48 (78.33, 82.63)  DBP:  I: 132.17 (128.70, 135.64)  C: 79.94 (77.8, 82.07) |  | SBP:  I: -3.64 (-6.23, 1.06)  C: -1.47 (-4.40, 1.46)  DBP:  I: +0.19 (-1.25, 1.61)  C: -0.25 (-1.59, 1.10) |
| Hwang et al. (2019) | SBP:  I: 134.92 ± 15.11  C: 133.93 ± 15.47  DBP:  I: 81.32 ± 11.44  C: 83.46 ± 10.40 | SBP:  I: 131.85 ± 14.82  C: 129.16 ± 14.72 p = 0.054  DBP:  I: 81.25 ± 11.47  C: 79.49 ± 11.36 p = 0.813 |  |
| Kasliwal et al. (2015) | SBP:  I: 127 ± 13  C: 124 ± 10  DBP:  I: 74 ± 9  C: 75 ± 8 | SBP:  I: 124 ± 10  C: 125 ± 14 p = 0.24  DBP:  I: 72 ± 6  C: 77 ± 9 p = 1.0 |  |
| Liu et al. (2018) | SBP:  I: 124.58 ± 13.7  C: 126.29 ± 16.53  DBP:  I: 74.65 ± 8.59  C: 74.61 ± 14.16 | SBP:  I: 124.79 ± 16.03  C: 128.68 ± 13.33 p = 0.887  DBP:  I: 73.26 ± 9.02  C: 75.14 ± 10.8  p = 0.774 | SBP:  I: +0.21 ± 12.11  C: +2.39 ± 12.27 p = 0.121  DBP:  I: -1.39 ± 7.84  C: +0.54 ±10.04 p = 0.077 |
| Njike et al. (2015), calorie-adusted | SBP:  I: 126.6 ± 16.1  C: NI  DBP:  I: 122.4 ± 11.6  C: NI |  | SBP:  I: -0.46 ± 11.20  C: +2.38 ± 13.33 p = 0.31  DBP:  I: +0.46 ± 6.42  C: +0.60 ± 7.36 p = 0.93 |
| Njike et al. (2015), ad libitum | SBP:  I: 122.4 ± 11.6  C: NI  DBP:  I: 72.9 ± 8.2  C: NI |  | SBP:  I: +0.51 ± 17.86  C: +1.98 ± 12.09 p = 0.60  DBP:  I: +0.82 ± 7.77  C: +1.80 ± 8.41 p = 0.52 |
| Tey et al. (2013) | SBP:  I (30g): 126 ± 3.69  C: 128 ± 2.42  DBP:  I (30g): 73.2 ± 2.32  C: 75.3 ± 1.76 | SBP:  I (30g): 124 ± 3.17  C: 123 ± 1.88  DBP:  I (30g): 72.6 ± 2.12  C: 72.9 ± 1.46 |  |
| Wang, D et al. (2021) | SBP:  I: 131.4 ± 16.0  C: 129.4 ± 13.9  DBP:  I: 87.4 ± 9.7  C: 88.1 ± 11.2 | SBP:  I: 129.4 ± 13.9  C: 13.5 ± 15.0 p = 0.718  DBP:  I: 87.0 ± 9.8  C: 86.7 ± 9.5 p = 0.347 |  |
| Wang, J et al. (2021) | SBP:  I: 121 (SEM 1.8)  C: 120 SEM 2.3)  DBP:  I: 76 (SEM 1.2)  C: 74 (SEM 1.4) | SBP:  I: 121 (SEM 1.8)  C: 118 (SEM 1.9)  DBP:  I: 77 (SEM 1.3)  C: 74 (SEM 1.5) |  |

^1^Abbreviations: DBP: diastolic blood pressure, SBP: systolic blood pressure. C: control group, I: intervention group, SEM: standard error

### Supplementary Table 9: Results from randomized controlled trials on blood glucose^1^

| Reference | Baseline values | Endpoint values | Mean change |
| --- | --- | --- | --- |
| Al-Abdrabalnabi et al. (2020) | I: 97.7 (96.4, 99.0) mg/dl  C: 96.8 (95.5, 98.1) mg/dl | I: 97.8 (96.4, 99.2) mg/dl  C: 95.7 (94.3, 97.2) mg/dl | I: +1 (0.99, 1.0) mg/dl  C: +0.99 (0.98, 1.0) p = 0.194 |
| Barbour et al. (2015) | I: 5.5 ± 0.7 mmol/l | I: 5.5 ± 0.6 mmol/l  C: 5.5 ± 0.5 mmol/l p = 0.614 |  |
| Casas-Augustench et al. (2011) | I: 5.82 ± 0.52 mmol/l  C: 5.82 ± 0.58 mmol/l | I vs. C: 0.01 (-0.25, 0.28) mmol/l p = 0.92 | I: -0.06 (-0.22, 0.11) mmol/l  C: -0.04 (-0.26, 0.18) mmol/l |
| Coates et al. (2020) | I: 5.6 ± 0.1 mmol/l  C: 5.6 ± 0.1 mmol/l | I: 5.6 ± 0.1 mmol/l  C: 5.5 ± 0.1 mmol/l p = 0.958 |  |
| Gulati et al. (2014) | I: 94.4 ± 9.6 mg/dl  C: 93.2 ± 7.8 mg/dl | I: 92.4 ± 7.9 mg/dl  C: 96.3 ± 7.9 mg/dl |  |
| Hernandez-Alonso et al. (2014) | I: 116.24 (112.37, 120.11) mg/dl  C: 108.06 (104.27, 111.84) mg/dl |  | I: -5.17 (-8.14, -2.14) mg/dl  C: +6.72 (4.38, 9.07) |
| Hunter et al. (2021) | I: 87 (SEM 2) mg/dl  C: 86.9 (SEM 2) mg/dl | I: 89.5 (SEM 2) mg/dl  C: 87.8 (SEM 2.1) mg/dl |  |
| Hwang et al. (2019) | I: 103.04 ± 27.44 mg/dl  C: 106.27 ± 24.62 mg/dl | I: 101.37 ± 16.46  C: 111.58 ± 30.20 p = 0.000 |  |
| Kasliwal et al. (2015) | I: 88.8 ± 7.1 mg/dl  C: 88.4 ± 8.6 mg/dl | I: 86.6 ± 6.3 mg/dl  C: 87.4 ± 8.6 mg/dl p = 0.83 |  |
| Madan et al. (2021) | I: 80.62 ± 7.14 mg/dl  C: 84.90 ± 11.6 mg/dl |  | I: +0.02 (-2.27, 2.31) mg/dl  C: -5.5 (-8.24, -2.75) mg/dl p = 0.01 |
| Njike et al. (2015), calorie-adjusted | I: 93.3 ± 8.2  C: NI |  | I: -1.75 (7.29) mg/dl  C: -0.33 (5.42) mg/dl p = 0.35 |
| Njike et al. (2015), ad libitum | I: 95.4 ± 10.3 mg/dl  C: NI |  | I: +0.02 ± 9.67 mg/dl  C: -1.08 ± 7.27 mg/dl p = 0.47 |
| Wang, D et al. (2021) | I: 5.55 (5.43, 5.57) mmol/l  C: 5.46 (5.35, 5.57) mmol/l | I: 5.22 (5.10, 5.35) mmol/l  C: 5.16 (5.04, 5.28) p = 0.802 |  |
| Wang, X et al. (2012) | I: 5.30 ± 0.85 mmol/l  C: 5.27 ± 0.79 mmol/l | I: 5.41 ± 0.63 mmol/l  C: 5.57 ± 0.90 mmol/l | I: +0.14 ± 0.68 mmol/l  C: +0.32 ± 0.65 mmol/l |

^1^Abbreviations: C: control group, I: intervention group, SEM: standard error

### Supplementary Table 10: Results from randomized controlled trials on HbA1c

| Reference | Baseline values | Endpoint values | Mean change |
| --- | --- | --- | --- |
| Gulati et al. (2014) | I: 5.9 ± 0.4 %  C: 5.8 ± 0.5 % | I: 5.7 ± 0.3 %  C: 5.8 ± 0.3 % p = 0.17 |  |
| Hernandez-Alonso et al. (2014) | I: 5.92 (5.82, 6.02) %  C: 5.87 (5.75, 5.99) % |  | I: -0.03 (-0.12, 0.05) %  C: 0.03 (-0.03, 0.10) % |
| Hunter et al. (2021) | I: 5.4 (SEM 0.04) %  C: 5.5 (SEM 0.04) % | I: 5.4 (SEM 0.04) %  C: 5.4 (SEM 0.04) % p = 0.98 |  |
| Hwang et al. (2019) | I: 5.39 ± 0.34 %  C: 5.38 ± 0.37 % | I: 5.38 ± 0.35 %  C: 5.43 ± 0.37 % p = 0.021 |  |
| Madan et al. (2021) | I: 5.38 ± 0.35 %  C: 5.33 ± 0.27 % |  | I: -0.04 (-0.12, 0.04) %  C: +0.09 (0.01, 0.16) % p = 0.02 |
| Njike et al. (2015), calorie-adjusted | I: 5.7 ± 0.4 %  C: NI |  | I: +0.05 ± 0.14 %  C: +0.06 ± 0.14 % p = 0.64 |
| Njike et al. (2015), ad libitum | I: 5.8 ± 0.4 %  C: NI |  | I: +0.10 ± 0.21 %  C: +0.04 ± 0.17 % p = 0.07 |

^1^Abbreviations: C: control group, I: intervention group, SEM: standard error

### Supplementary Table 11: Results from randomized controlled trials on insulin resistance (HOMA-IR)^1^

| Reference | Baseline values | Endpoint values | Mean change |
| --- | --- | --- | --- |
| Barbour et al. (2015) | 1.1 ± 0.6 | I: 1.1 ± 0.7  C: 1.1 ± 0.6  p = 0.769 |  |
| Casas-Agustench et al. (2011) | I: 2.10 ± 1.01  C: 1.56 ± 1.14 | I vs. C: -0.67 (-1.25, -0.11) p = 0.021 | I: -0.58 (-0.98, -0.18)  C: +0.14 (-0.28, 0.55) |
| Coates et al. (2020) | I: 0.96 ± 0.06  C: 1.0 ± 0.06 | I: 0.99 ± 0.06  C: 0.95 ± 0.06 p = 0.963 |  |
| Hernandez-Alonso et al. (2014) | I: 4.22 (3.66, 4.77)  C: 3.10 (2.64, 3.56) |  | I: -0.69 (-1.07, -0.31)  C: +0.97 (0.49, 1.44) |
| Hunter et al. (2021) | I: 3.2 (SEM 0.4)  C: 2.6 (SEM 0.4) | I: 3.4 (SEM 0.4)  C: 2.4 (SEM 0.4) p = 0.56 |  |
| Madan et al. (2021) | I: 2.25 ± 1.34  C: 2.45 ± 1.25 |  | I: +0.61 (-0.44, 1.67)  C: -0.19 (-0.43, 0.04)  p = 0.13 |

^1^Abbreviations: C: control group, I: intervention group, SEM: standard error

### Supplementary Table 12: Subgroup analysis of high vs. low nuts and seeds consumption and risk of total cardiovascular disease

| Subgroup | N studies | Relative risk (95 % CI) | *p* for group differences |
| --- | --- | --- | --- |
| Region | | | 0.35 |
| Europe | 2 | 0.65 (0.36, 1.17) |  |
| USA | 8 | 0.78 (0.71, 0.85) |  |
| Asia | 3 | 0.87 (0.75, 1.01) |  |
| Australia | 1 | 0.90 (0.73, 1.11) |  |
| Multinational | 1 | 0.87 (0.79, 0.97) |  |
| Follow-up time | | | 0.35 |
| <10 years | 6 | 0.77 (0.69, 0.86) |  |
| ≥10 years | 9 | 0.82 (0.76, 0.90) |  |
| Adjusted for hypercholesterolaemia | | | 0.34 |
| No | 10 | 0.78 (0.73, 0.84) |  |
| Yes | 5 | 0.84 (0.73, 0.98) |  |
| Adjusted for hypertension | | | 0.48 |
| No | 6 | 0.79 (0.71, 0.88) |  |
| Yes | 9 | 0.83 (0.77, 0.89) |  |
| Sex | | | 0.40 |
| Male | 6 | 0.87 (0.81, 0.93) |  |
| Female | 8 | 0.82 (0.74, 0.91) |  |
| Risk of bias | | | 0.10 |
| Moderate | 7 | 0.85 (0.78, 0.93) |  |
| Serious | 8 | 0.77 (0.71, 0.83) |  |
| **Overall** | **15** | **0.81 (0.75, 0.86)** |  |

### Supplementary Table 13: Subgroup analysis of high vs. low nuts and seeds consumption and risk of cardiovascular disease mortality

| Subgroup | N studies | Relative risk (95 % CI) | p for group differences |
| --- | --- | --- | --- |
| Region | | | 0.60 |
| Europe | 2 | 0.65 (0.36, 1.17) |  |
| USA | 8 | 0.76 (0.69, 0.84) |  |
| Asia | 3 | 0.79 (0.69, 0.92) |  |
| Australia | 1 | 0.90 (0.73, 1.11) |  |
| Multinational | 1 | 0.72 (0.56, 0.92) |  |
| Follow-up time | | | 0.27 |
| <10 years | 6 | 0.73 (0.66, 0.80) |  |
| ≥10 years | 9 | 0.79 (0.71, 0.87) |  |
| Adjusted for hypercholesterolaemia | | | 0.87 |
| No | 10 | 0.74 (0.70, 0.78) |  |
| Yes | 5 | 0.75 (0.63, 0.90) |  |
| Adjusted for hypertension | | | 0.87 |
| No | 6 | 0.76 (0.66, 0.88) |  |
| Yes | 9 | 0.77 (0.72, 0.83) |  |
| Sex | | | 0.70 |
| Male | 6 | 0.80 (0.73, 0.89) |  |
| Female | 8 | 0.78 (0.70, 0.87) |  |
| Risk of bias | | | 0.42 |
| Moderate | 8 | 0.78 |  |
| Serious | 7 | 0.74 |  |
| **Overall** | **15** | **0.77 (0.72, 0.82)** |  |

### Supplementary Table 14: Subgroup analysis of high vs. low nuts and seeds consumption and risk of coronary heart disease

| Subgroup | N studies | Relative risk (95 % CI) | p for group differences |
| --- | --- | --- | --- |
| Region | | | 0.08 |
| Europe | 3 | 0.91 (0.85, 0.98) |  |
| USA | 7 | 0.76 (0.67, 0.85) |  |
| Asia | 1 | 0.98 (0.76, 1.27) |  |
| Australia | 1 | 0.95 (0.74, 1.21) |  |
| Multinational | 1 | 0.95 (0.72, 1.03) |  |
| Follow-up time | | | 0.04 |
| <10 years | 4 | 0.71 (0.59, 0.85) |  |
| ≥10 years | 9 | 0.87 (0.82, 0.93) |  |
| Adjusted for hypercholesterolaemia | | | 0.21 |
| No | 6 | 0.78 (0.69, 0.88) |  |
| Yes | 7 | 0.86 (0.78, 0.94) |  |
| Adjusted for hypertension | | | 0.36 |
| No | 3 | 0.78 (0.73, 0.84) |  |
| Yes | 10 | 0.83 (0.75, 0.92) |  |
| Sex |  | | 0.66 |
| Male | 4 | 0.88 (0.79, 0.96) |  |
| Female | 4 | 0.83 (0.65, 1.05) |  |
| Risk of bias |  | | 0.64 |
| Moderate | 6 | 0.84 (0.75, 0.94) |  |
| Serious | 7 | 0.81 (0.72, 0.91) |  |
| **Overall** | **13** | **0.82 (0.76, 0.89)** |  |

### Supplementary Table 15: Subgroup analysis of high vs. low nuts and seeds consumption and risk of stroke

| Subgroup | N studies | Relative risk (95 % CI) | p for group differences |
| --- | --- | --- | --- |
| Region | | | 0.50 |
| Europe | 2 | 0.86 (0.79, 0.94) |  |
| USA | 5 | 0.93 (0.82, 1.05) |  |
| Asia | 1 | 1.13 (0.68, 1.87) |  |
| Australia | 1 | 0.98 (0.84, 1.14) |  |
| Multinational | 1 | 0.88 (0.60, 1.29) |  |
| Follow-up time | | | 0.74 |
| <10 years | 3 | 0.89 (0.76, 1.04) |  |
| ≥10 years | 7 | 0.92 (0.85, 1.00) |  |
| Adjusted for hypercholesterolaemia | | | 0.57 |
| No | 5 | 0.89 (0.78, 1.00) |  |
| Yes | 5 | 0.93 (0.84, 1.02) |  |
| Adjusted for hypertension | | | 0.73 |
| No | 2 | 0.89 (0.74, 1.07) |  |
| Yes | 8 | 0.92 (0.85, 0.99) |  |
| Sex |  | | 0.76 |
| Male | 5 | 0.98 (0.83, 1.16) |  |
| Female | 3 | 0.94 (0.76, 1.17) |  |
| Risk of bias |  | | 0.36 |
| Moderate | 5 | 0.95 (0.85, 1.07) |  |
| Serious | 5 | 0.89 (0.82, 0.97) |  |
| **Overall** | **10** | **0.91 (0.85, 0.97)** |  |

### Supplementary Table 16: Subgroup analysis of effects of nut consumption on total cholesterol

| Subgroup | N comparisons | Mean difference, mmol/l (95 % CI) | p for group differences |
| --- | --- | --- | --- |
| Intervention | | | 0.43 |
| Mixed | 2 | 0.13 (-0.40, 0.65) |  |
| Almonds | 4 | -0.19 (-0.40, 0.02) |  |
| Peanuts | 2 | -0.02 (-0.17, 0.13) |  |
| Pistachio nuts | 4 | -0.20 (-0.34, -0.05) |  |
| Walnuts | 5 | -0.20 (-0.33, -0.07) |  |
| Hazelnuts | 1 | -0.13 (-0.57, 0.31) |  |
| Dose (median = 50 g) | | | 0.57 |
| ≥ median | 9 | -0.12 (-0.21, -0.03) |  |
| < median | 9 | -0.17 (-0.30, -0.04) |  |
| Duration | | | 0.82 |
| 12 weeks | 10 | -0.15 (-0.32, 0.01) |  |
| >12 weeks | 8 | -0.13 (-0.15, -0.11) |  |
| Population | | | 0.03 |
| Generally healthy | 7 | -0.13 (-0.15, -0.11) |  |
| Dyslipidaemia | 2 | -0.45 (-0.67, -0.23) |  |
| Metabolic syndrome | 7 | -0.10 (-0.23, 0.04) |  |
| Prediabetes | 2 | -0.25 (-0.56, 0.05) |  |
| Region | | | 0.63 |
| Europe | 2 | 0.08 (-0.43, 0.60) |  |
| USA | 5 | -0.13 (-0.15, -0.11) |  |
| Asia | 8 | -0.21 (-0.37, -0.06) |  |
| Australia/New Zealand | 3 | -0.12 (-032, 0.09) |  |
| Risk of bias | | | 0.46 |
| Low | 2 | -0.16 (0.40, 0.09) |  |
| Some concerns | 10 | -0.13 (-0.15, -0.11) |  |
| High | 6 | -0.26 (-0.46, -0.05) |  |
| **Overall** | **18** | **-0.15 (-0.22, -0.08)** |  |

### Supplementary Table 17: Subgroup analysis of effects of nut consumption on LDL cholesterol

| Subgroup | N comparisons | Mean difference, mmol/l (95 % CI) | p for group differences |
| --- | --- | --- | --- |
| Intervention | | | 0.01 |
| Mixed | 1 | 0.29 (-0.01, 0.59 |  |
| Almonds | 4 | -0.22 (-0.33, -0.12) |  |
| Peanuts | 2 | 0.02 (-0.13, 0.17) |  |
| Pistachio nuts | 4 | -0.17 (-0.28, -0.05) |  |
| Walnuts | 5 | -0.15 (-0.30, -0.00) |  |
| Hazelnuts | 1 | -0.12 (-0.54, 0.30) |  |
| Dose (median = 54 g) | | | 0.89 |
| ≥ median | 9 | -0.13 (-0.22, -0.05) |  |
| < median | 8 | -0.12 (-0.27, 0.03) |  |
| Duration | | | 0.94 |
| 12 weeks | 8 | -0.11 (-0.27, 0.06) |  |
| >12 weeks | 9 | -0.11 (-0.16, -0.07) |  |
| Population |  |  | <0.01 |
| Generally healthy | 6 | -0.09 (-0.12, -0.07) |  |
| Dyslipidaemia | 2 | -0.38 (-0.50, -0.27) |  |
| Metabolic syndrome | 7 | -0.03 (-0.17, 0.10) |  |
| Prediabetes | 2 | -0.21 (-0.33, -0.08) |  |
| Region | | | 0.22 |
| Europe | 2 | 0.06 (-0.35, 0.48) |  |
| USA | 4 | -0.09 (-0.11, -0.07) |  |
| Asia | 8 | -0.20 (-0.31, -0.09) |  |
| Australia/New Zealand | 3 | -0.10 (-0.28, 0.08) |  |
| Risk of bias | | | 0.02 |
| Low | 2 | -0.09 (-0.39, 0.20) |  |
| Some concerns | 10 | -0.09 (-0.11, -0.07) |  |
| High | 5 | -0.26 (-0.37, -0.15) |  |
| **Overall** | **17** | **-0.13 (-0.21, -0.05)** |  |

### Supplementary Table 18: Subgroup analysis of effects of nut consumption on systolic blood pressure

| Subgroup | N comparisons | Mean difference, mmHg (95 % CI) | p for group differences |
| --- | --- | --- | --- |
| Intervention | | | 0.29 |
| Mixed | 2 | 3.55 (-0.63, 7.73) |  |
| Almonds | 2 | -2.61 (-6.42, 1.21) |  |
| Peanuts | 1 | -2.10 (-5.89, 1.69) |  |
| Pistachio nuts | 2 | -1.94 (-5.20, 1.31) |  |
| Walnuts | 4 | -0.67 (-2.86, 1.52) |  |
| Hazelnuts | 1 | 1.00 (-6.03, 8.03) |  |
| Dose (median = 54 g) | | | 0.05 |
| ≥ median | 6 | -2.29 (-4.29, -0.29) |  |
| < median | 6 | 0.84 (-1.51, 3.19) |  |
| Duration | | | 0.47 |
| 12 weeks | 6 | -0.11 (-2.58, 2.36) |  |
| >12 weeks | 6 | -1.16 (-2.61, 0.28) |  |
| Population | | | 0.81 |
| Generally healthy | 5 | -1.03 (-2.59, 0.52) |  |
| Dyslipidaemia | 1 | -1.00 (-8.36, 6.36) |  |
| Metabolic syndrome | 5 | 0.10 (-2.79, 3.00) |  |
| Prediabetes | 1 | -2.17 (-5.80, 1.46) |  |
| Region | | | 0.94 |
| Europe | 2 | 0.49 (-5.91, 6.89) |  |
| USA | 3 | 0.21 (-3.94, 4.35) |  |
| Asia | 4 | -0.58 (-3.28, 2.12) |  |
| Australia/New Zealand | 2 | -1.55 (-5.78, 2.67) |  |
| Spain & USA | 1 | -1.31 (-3.18, 0.56) |  |
| Risk of bias | | | 0.20 |
| Low | 2 | -2.40 (-5.48, 0.67) |  |
| Some concerns | 6 | -1.16 (-2.67, 0.35) |  |
| High | 4 | 1.11 (-1.58, 3.80) |  |
| **Overall** | **12** | **-0.89 (-2.10, 0.32)** |  |

### Supplementary Table 19: Subgroup analysis of effects of nut consumption on diastolic blood pressure

| Subgroup | N comparisons | Mean difference, mmHg (95 % CI) | p for group differences |
| --- | --- | --- | --- |
| Intervention | | | 0.35 |
| Mixed | 2 | 2.53 (-0.32, 5.37) |  |
| Almonds | 2 | -1.25 (-3.59, 1.08) |  |
| Peanuts | 1 | 0.30 (-2.32, 2.92) |  |
| Pistachio nuts | 2 | -1.85 (-7.11, 3.42) |  |
| Walnuts | 4 | -0.79 (-2.03, 0.45) |  |
| Hazelnuts | 1 | -0.30 (-5.26, 4.66) |  |
| Dose (median = 54 g) | | | 0.92 |
| ≥ median | 6 | -0.52 (-1.30, 0.99) |  |
| < median | 6 | -0.04 (-2.13, 2.06) |  |
| Duration |  | | 0.69 |
| 12 weeks | 6 | -0.05 (-1.86, 1.76) |  |
| >12 weeks | 6 | -0.48 (-1.48, 0.53) |  |
| Population | | | 0.11 |
| Generally healthy | 5 | -0.74 (-1.89, 0.40) |  |
| Dyslipidaemia | 1 | -5.00 (-9.63, -0.37) |  |
| Metabolic syndrome | 5 | 0.45 (-1.18, 2.07) |  |
| Prediabetes | 1 | 0.44 (-1.38, 2.26) |  |
| Region | | | 0.45 |
| Europe | 2 | 0.69 (-0.98, 2.36) |  |
| USA | 3 | 0.66 (-1.64, 2.96) |  |
| Asia | 4 | -1.00 (-3.56, 1.56) |  |
| Australia/New Zealand | 2 | -0.83 (-3.25, 1.58) |  |
| Spain & USA | 1 | -1.10 (-2.55, 0.35) |  |
| Risk of bias | | | 1.00 |
| Low | 2 | -0.32 (-2.22, 1.59) |  |
| Some concerns | 6 | -0.32 (-1.37, 0.73) |  |
| High | 4 | -0.46 (-4.04, 3.12) |  |
| **Overall** | **12** | **-0.33 (-1.16, 0.50)** |  |


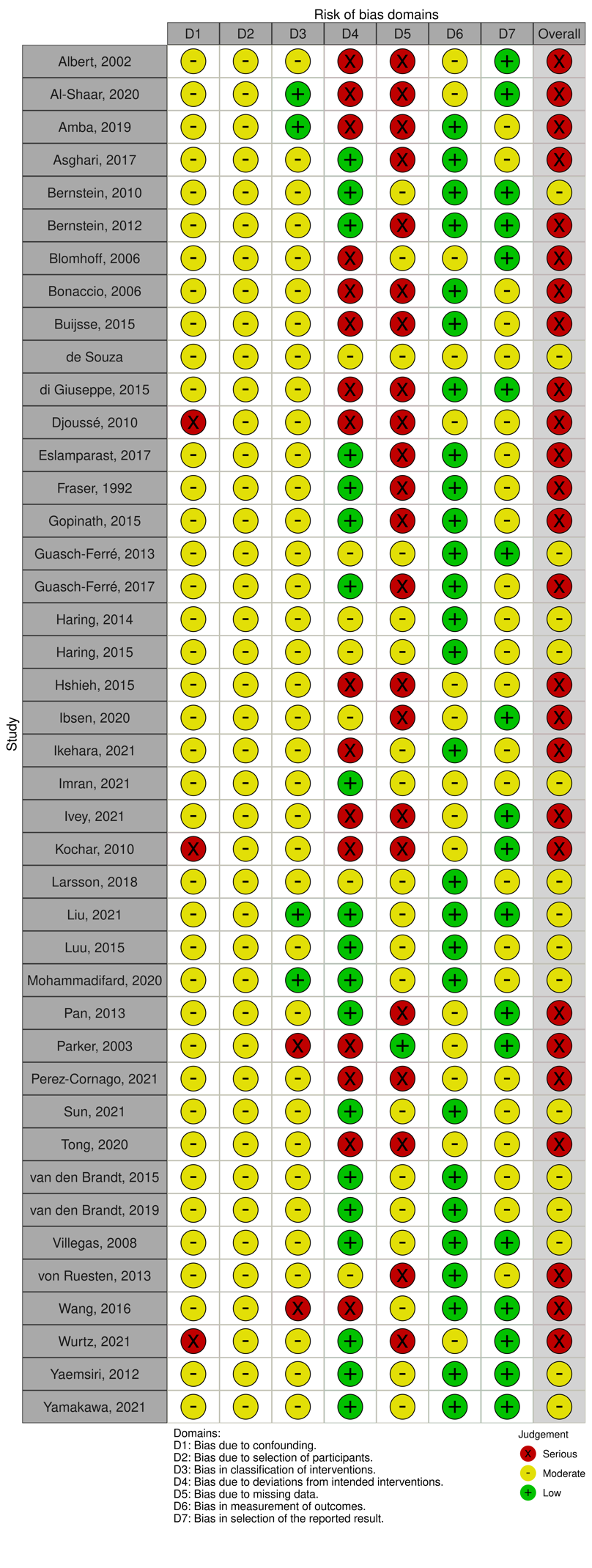


## Supplementary figures

### Supplementary figure 1. Risk of bias assessment of cohort studies

### Suppl. Figure 2


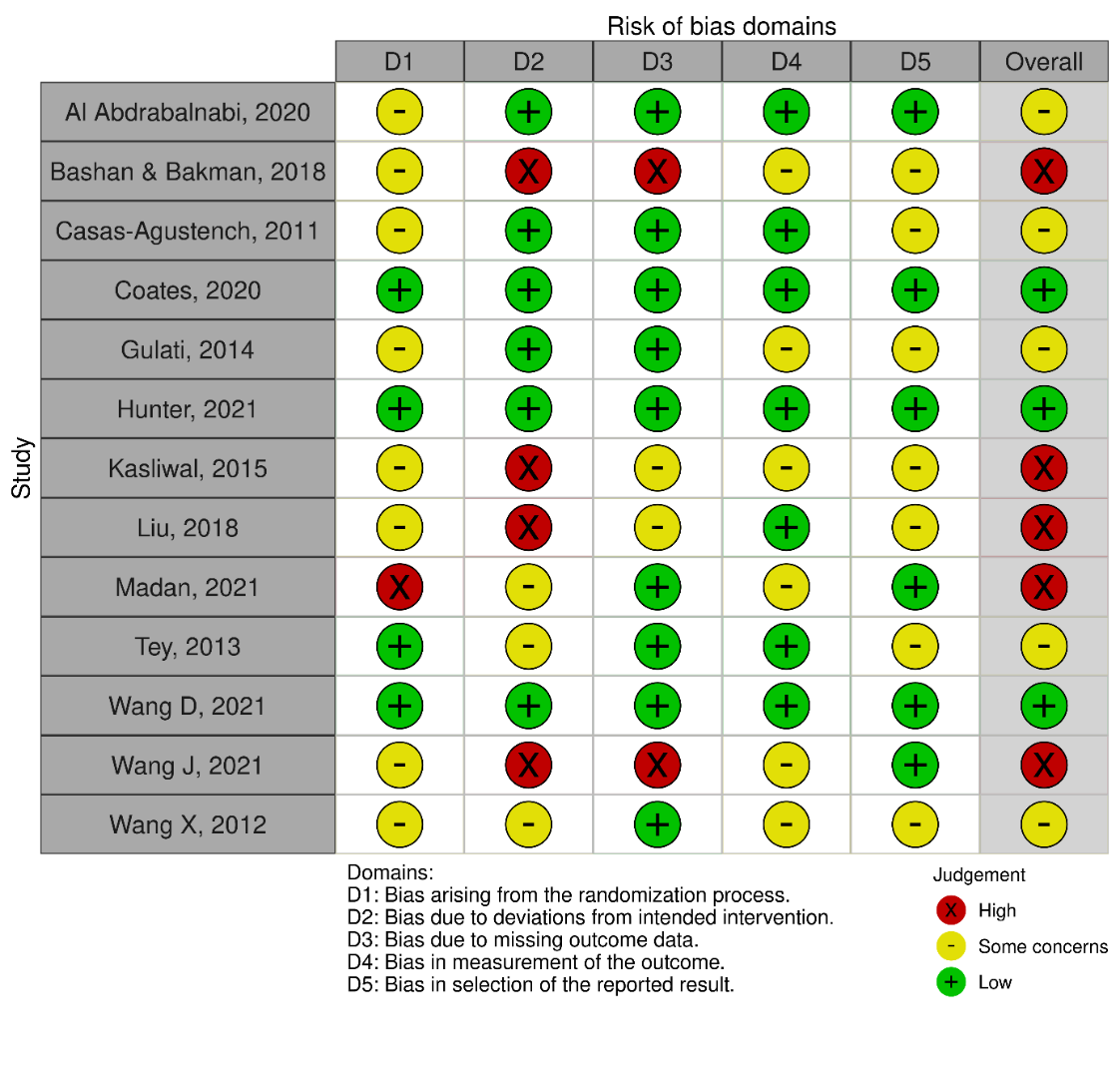


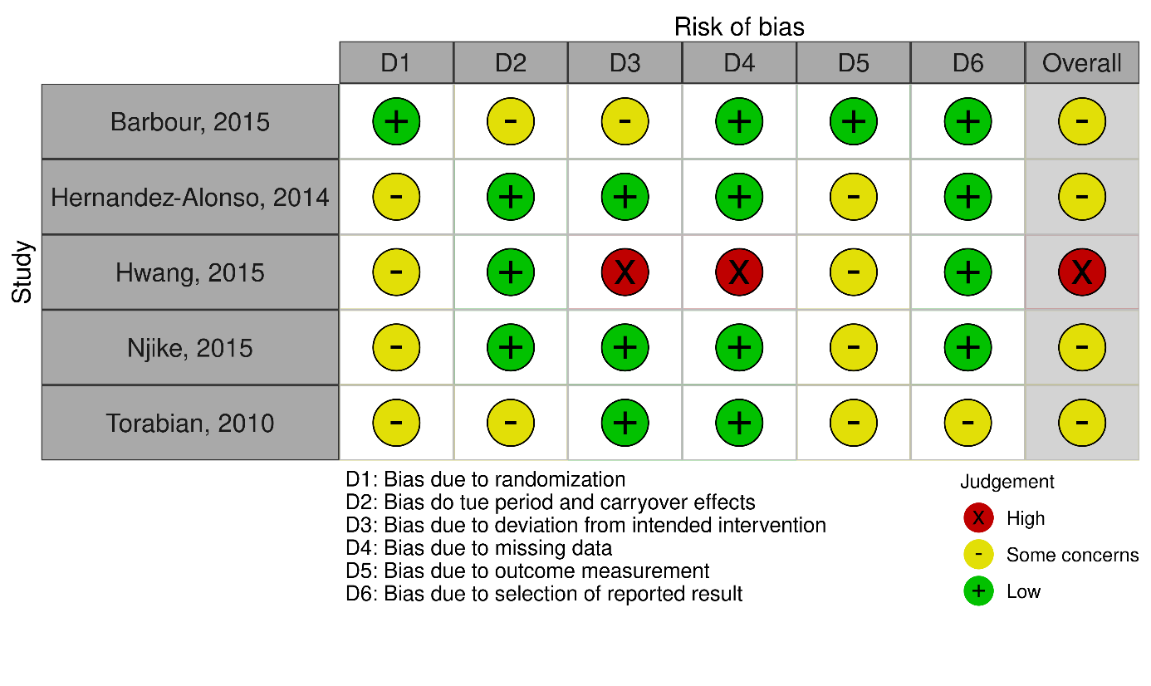


Supplementary Figure 2. Risk of bias assessment of randomized controlled trials. Top: parallel group RCTs, bottom: crossover RCTs.

### Supplementary Figure 3A-I


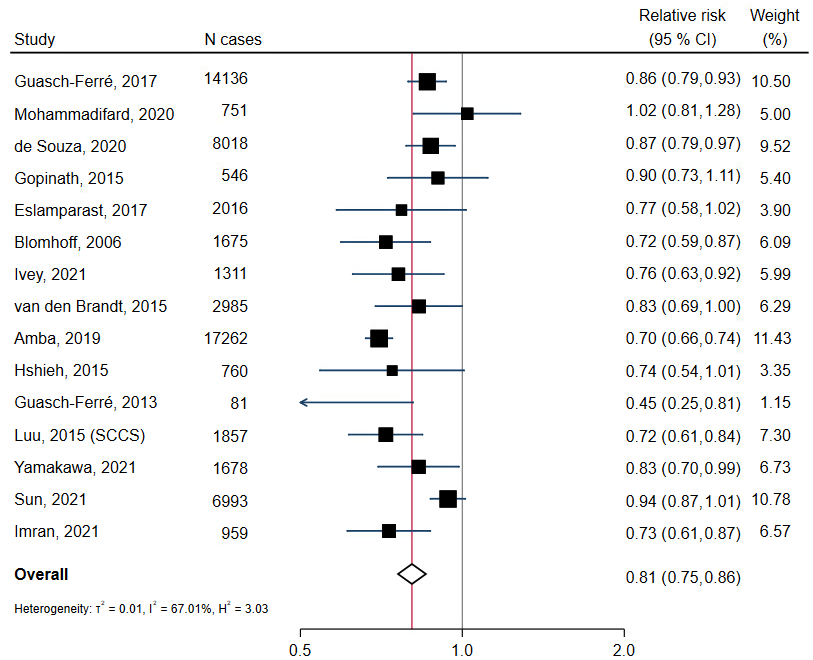


Supplementary figure 3A: Total nuts and seeds consumption and risk of cardiovascular disease, high vs. low consumption.


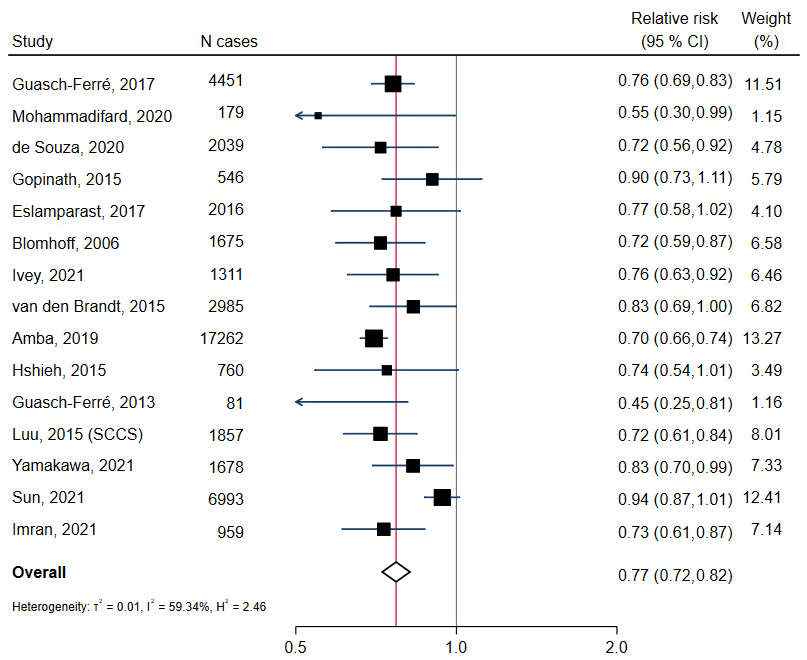


Supplementary figure 3B: Total nuts and seeds consumption and risk of cardiovascular disease mortality, high vs. low consumption.


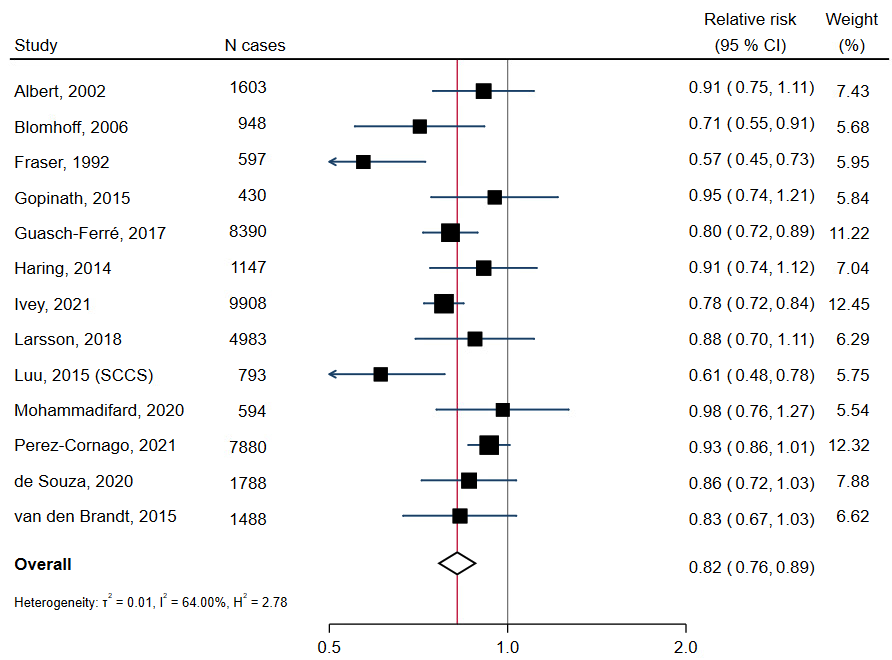


Supplementary figure 3C: Total nuts and seeds consumption and risk of coronary heart disease, high vs. low consumption.


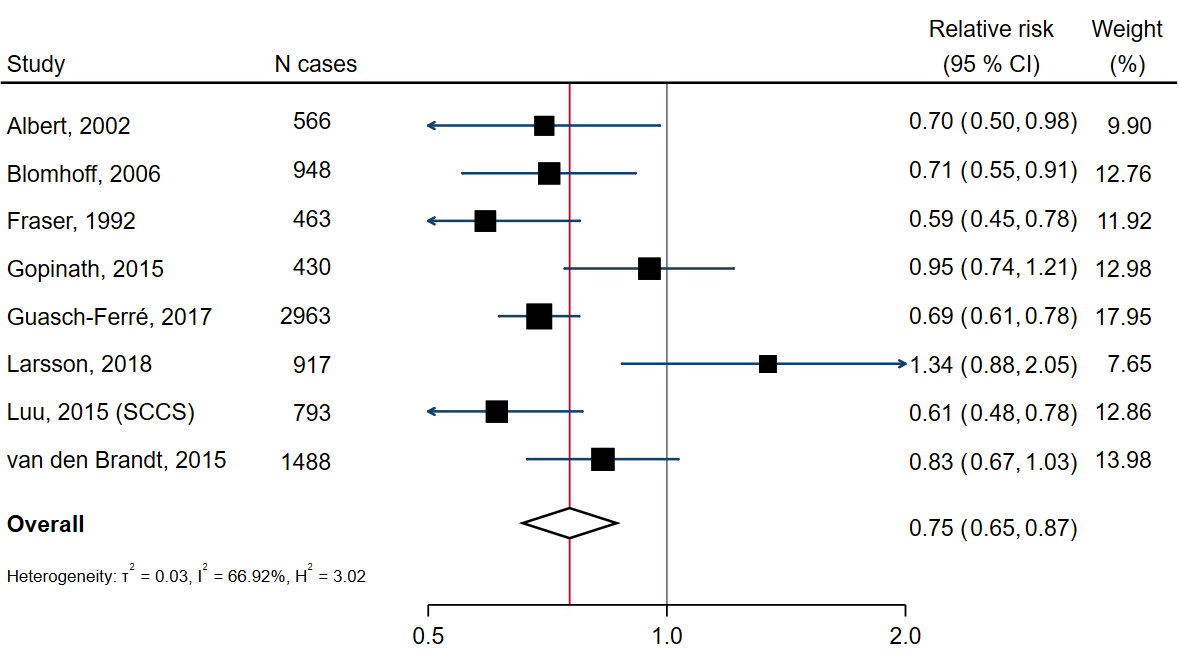


Supplementary figure 3D: Total nuts and seeds consumption and risk of coronary heart disease mortality, high vs. low consumption.


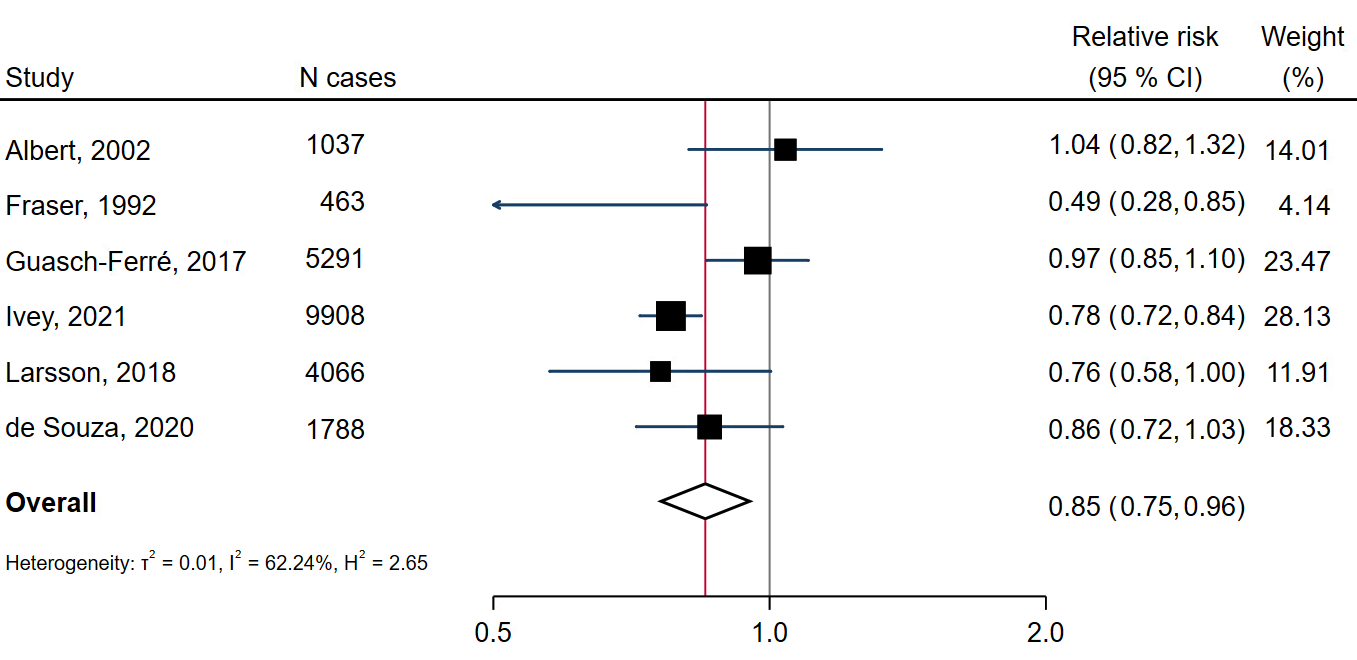


Supplementary figure 3E: Total nuts and seeds consumption and risk of nonfatal coronary heart disease, high vs. low consumption.


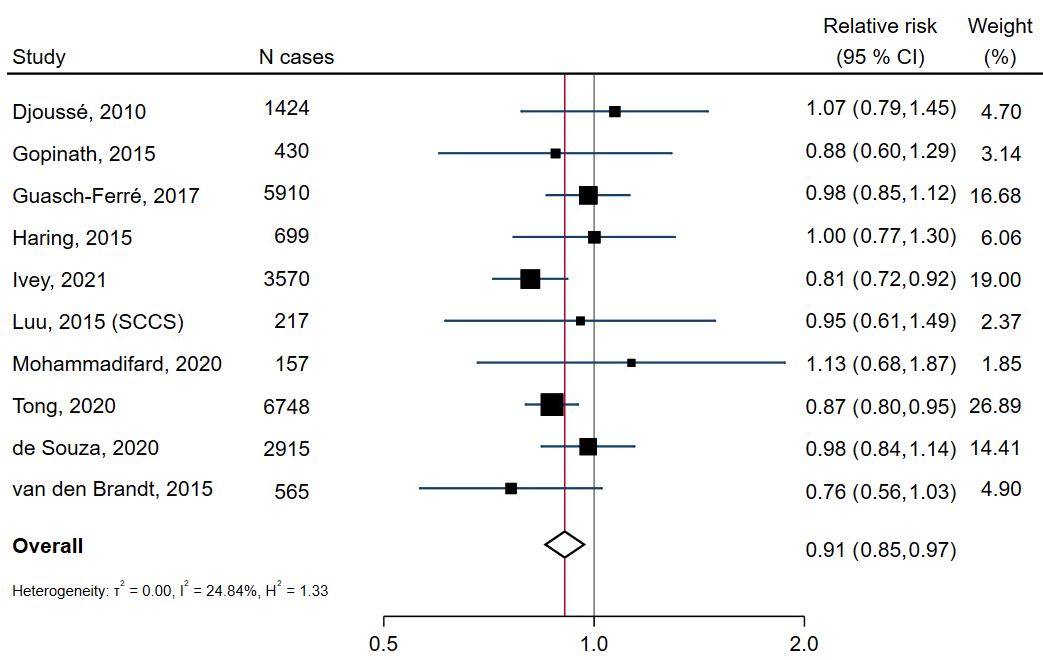


Supplementary figure 3F: Total nuts and seeds consumption and risk of total stroke, high vs. low consumption.


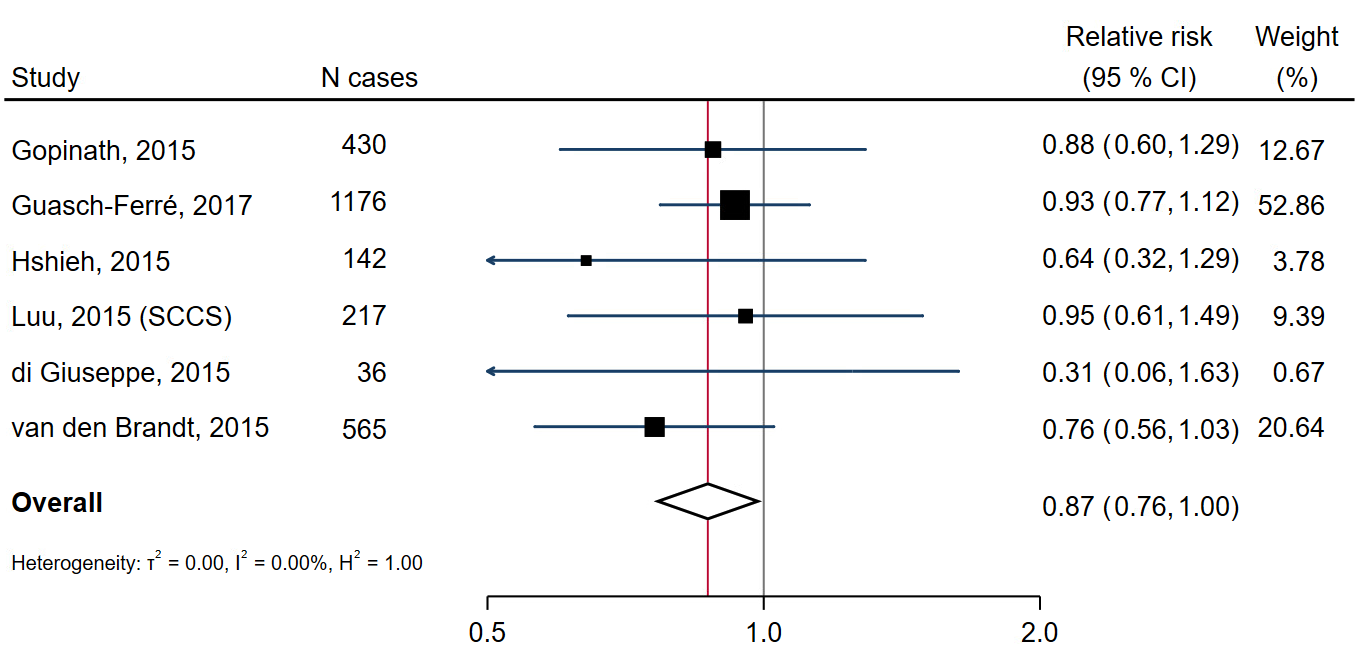


Supplementary figure 3G: Total nuts and seeds consumption and risk of stroke mortality, high vs. low consumption.


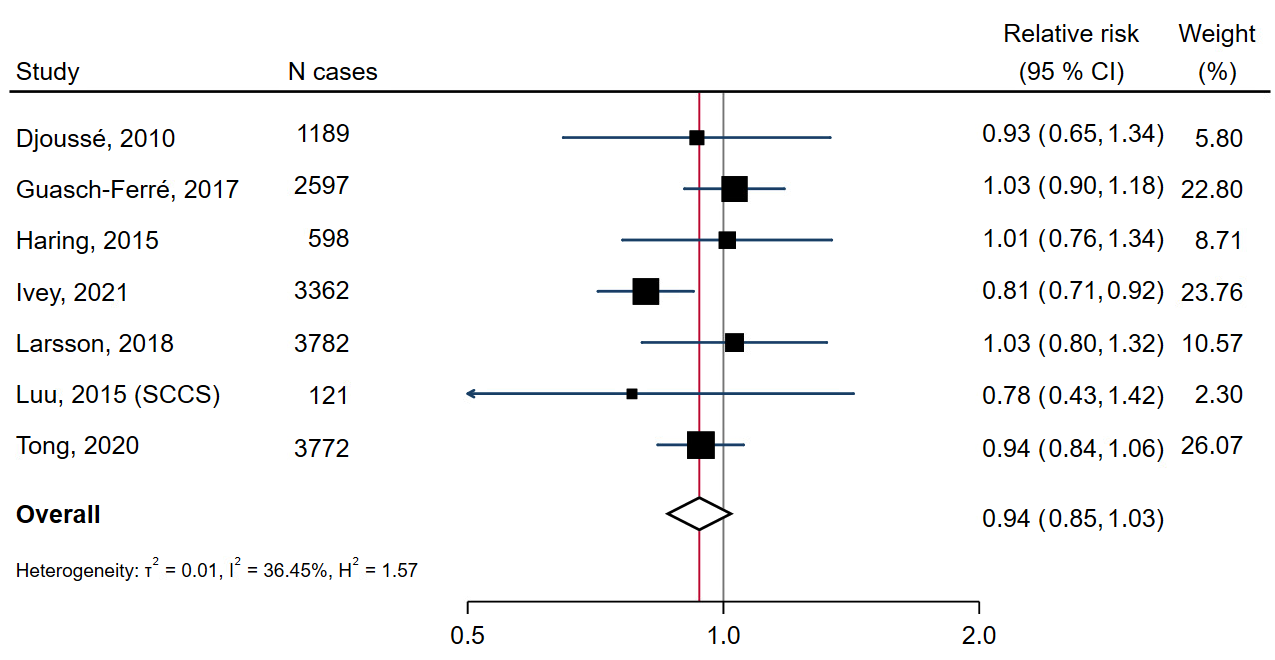


Supplementary figure 3H: Total nuts and seeds consumption and risk of ischaemic stroke, high vs. low consumption.

Supplementary figure 3I: Total nuts and seeds consumption and risk of type 2 diabetes, high vs. low consumption.

### Supplementary figures 4A-M


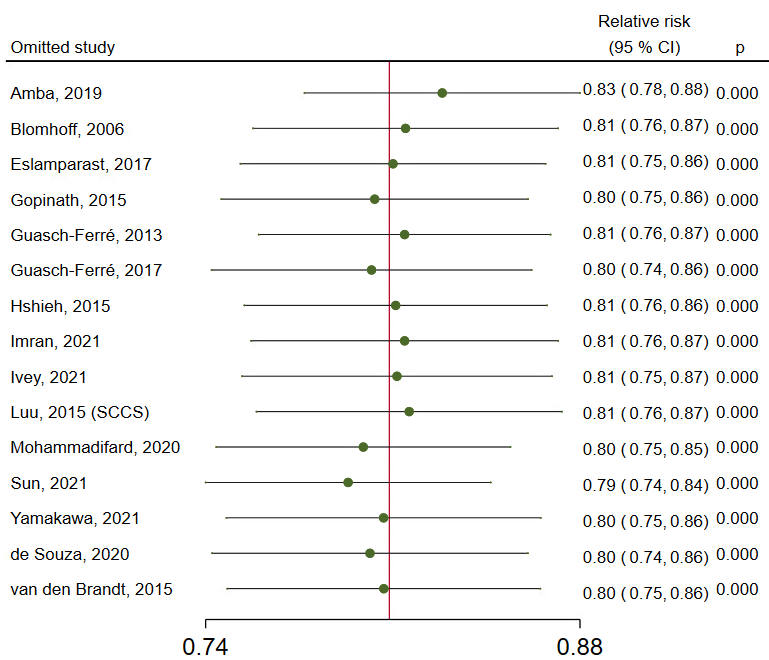


Supplementary figure 4A: Pooled relative risk of total cardiovascular disease after omitting one study at the time.


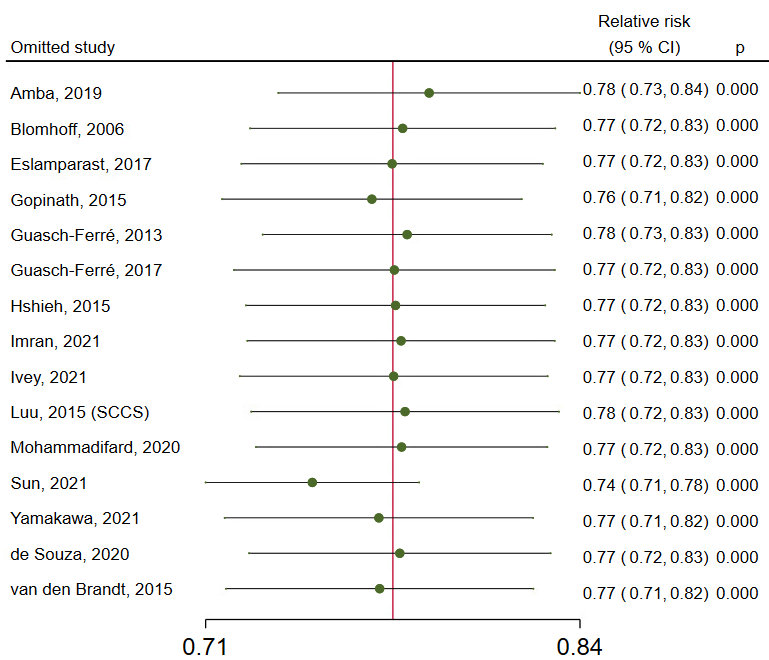


Supplementary figure 4B: Pooled relative risk of cardiovascular disease mortality after omitting one study at the time.


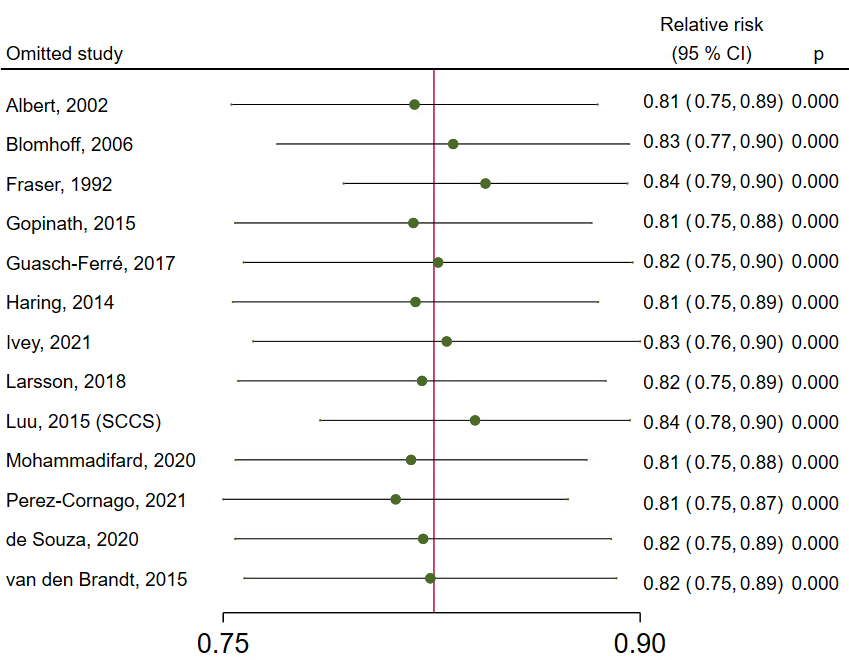


Supplementary figure 4C: Pooled relative risk of total coronary heart disease after omitting one study at the time.


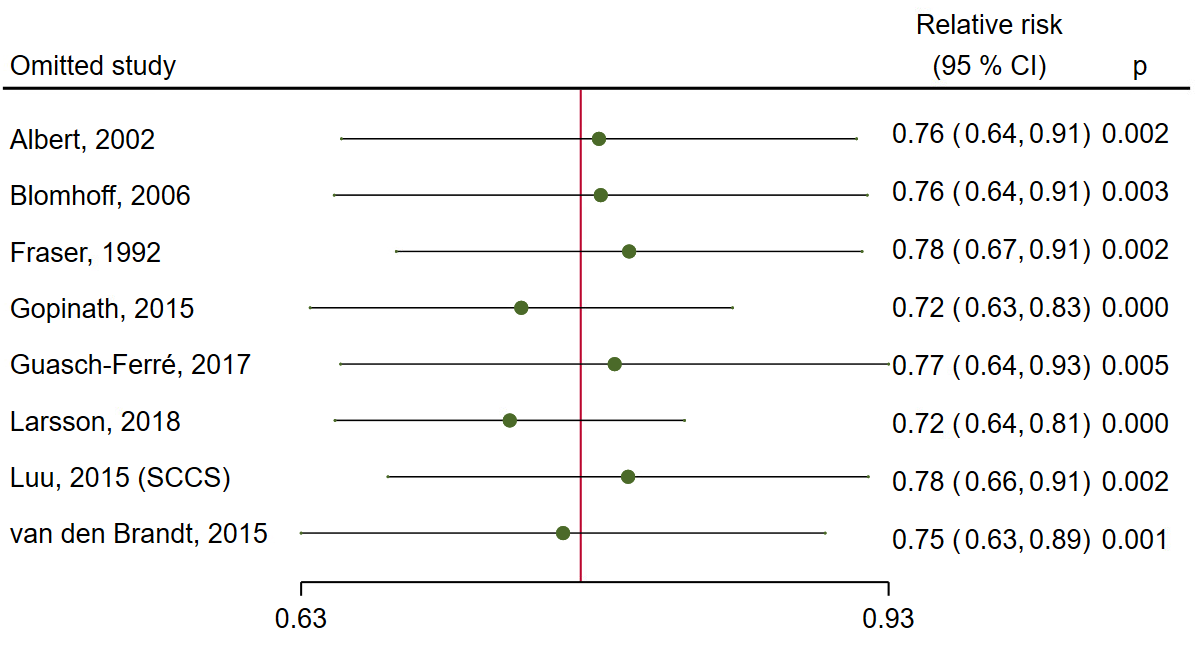


Supplementary figure 4D: Pooled relative risk of coronary heart disease mortality after omitting one study at the time.


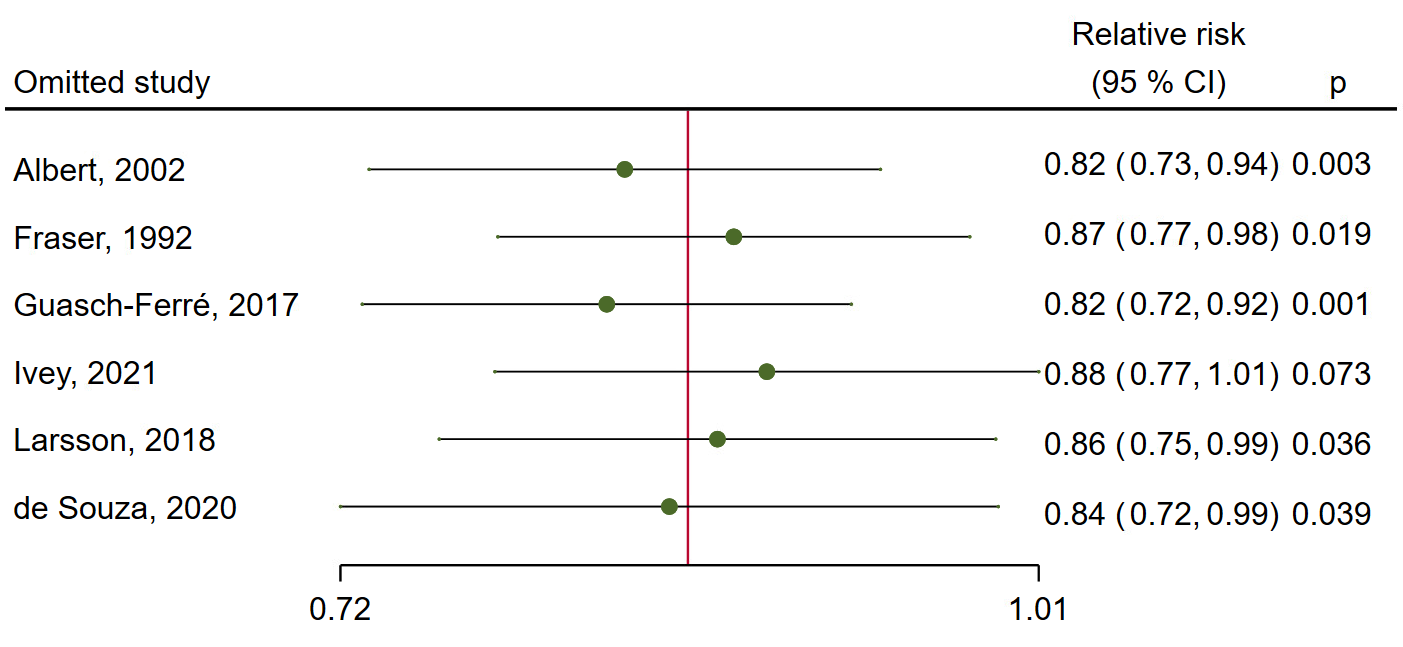


Supplementary figure 4E: Pooled relative risk of nonfatal coronary heart disease after omitting one study at the time.


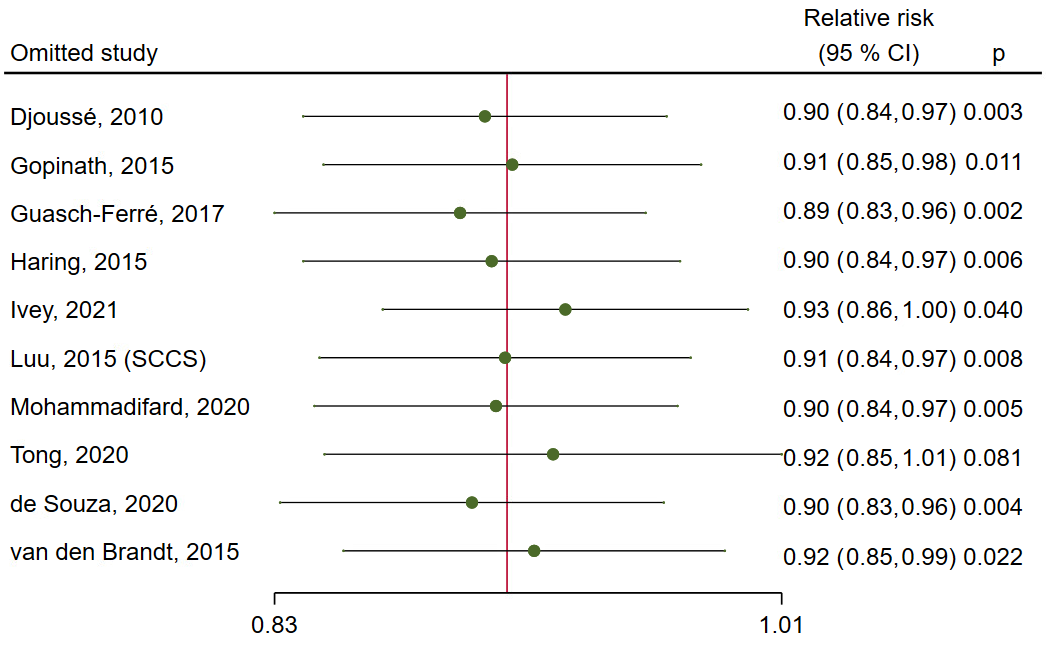


Supplementary figure 4F: Pooled relative risk of total stroke after omitting one study at the time.


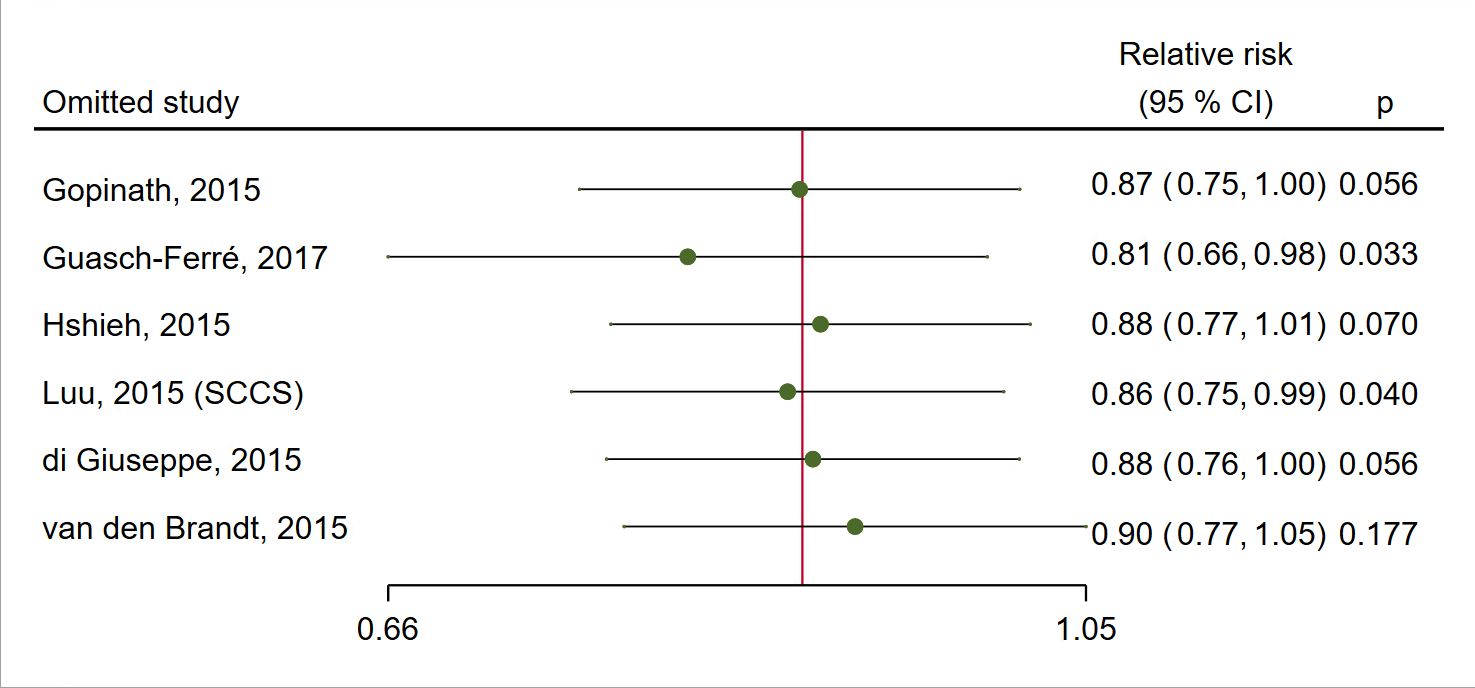


Supplementary figure 4G: Pooled relative risk of stroke mortality after omitting one study at the time.


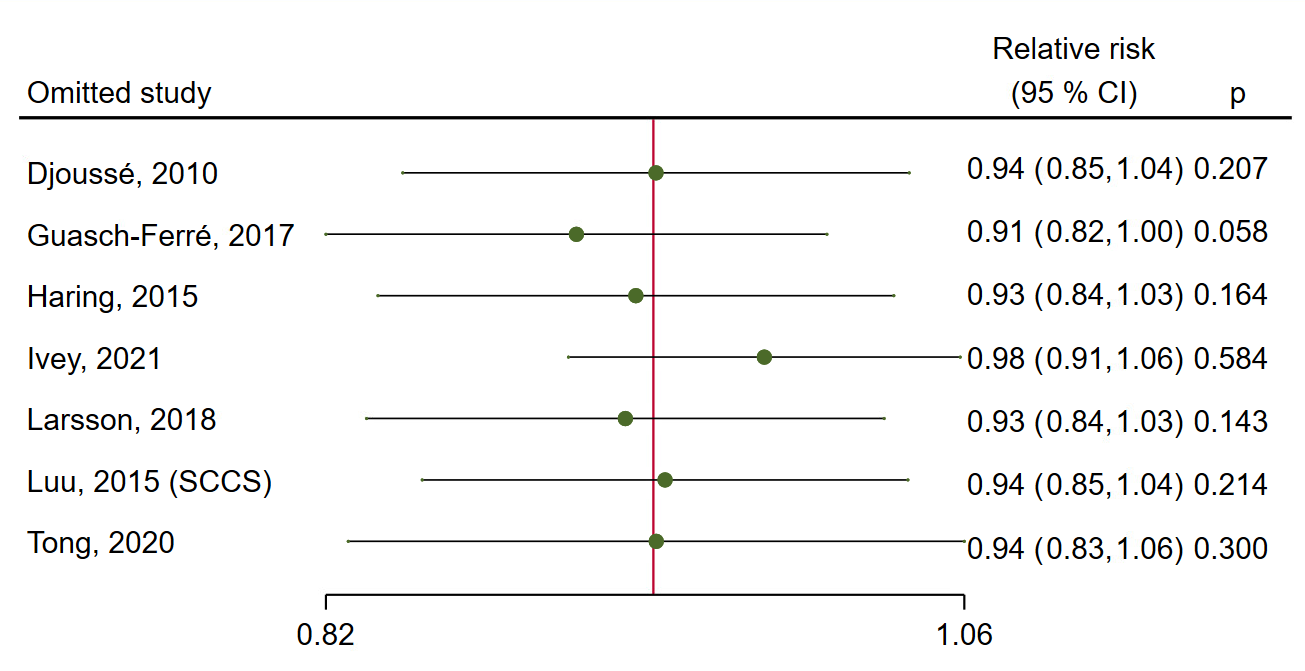


Supplementary figure 4H: Pooled relative risk of ischaemic stroke after omitting one study at the time.

Supplementary figure 4I: Pooled relative risk of type 2 diabetes after omitting one study at the time.

Supplementary figure 4J: Pooled effect estimate for total cholesterol after omitting one study at the time.

Supplementary figure 4K: Pooled effect estimate for LDL cholesterol after omitting one study at the time.

Supplementary figure 4L: Pooled effect estimate for systolic blood pressure after omitting one study at the time.

Supplementary figure 4M: Pooled effect estimate for diastolic blood pressure after omitting one study at the time.

### Supplementary figure 5

A

**B**

Supplementary figure 5. Peanut consumption and risk of total cardiovascular disease. A: Forest plot showing relative risk (95 % confidence interval) with high vs. low consumption. B: Linear (red, dashed line) and non-linear dose-response (black lines with confidence intervals) association between peanut consumption and risk of total cardiovascular disease in cohort studies (5 studies), with 0 grams/day as reference. Circles show the effect estimates for each level of intake in the individual studies, weighted by the inverse of the standard errors. Vertical axis is log scaled.

### Supplementary figure 6A-H

A

B

C

D

E

F

G

H

Supplementary figure 6A-H: Contour-enhanced funnel plots for small-study effects estimated with random-effects restricted maximum likelihood models. Red vertical lines correspond to the estimated overall effect size. A: Total cardiovascular disease, B: cardiovascular disease mortality, C: total coronary heart disease, D: Stroke, E: Total cholesterol, F: LDL cholesterol, G: systolic blood pressure, H: diastolic blood pressure
